# Formulary Restrictions and Relapse Episodes in Persons With Relapsing-Remitting Multiple Sclerosis

**DOI:** 10.1001/jamanetworkopen.2025.25155

**Published:** 2025-08-01

**Authors:** Barbara Blaylock, Karen Van Nuys, Geoffrey Joyce

**Affiliations:** 1 Blaylock Health Economics LLC, Reno, Nevada; 2 Leonard D. Schaeffer Center for Health Policy and Economics, University of Southern California, Los Angeles; 3 Department of Pharmaceutical and Health Economics, Alfred E. Mann School of Pharmacy and Pharmaceutical Sciences, University of Southern California, Los Angeles

## Abstract

**Importance:**

Narrow formularies can be used to increase rebates and manage the use of costly drug therapies in the US.

**Objective:**

To examine the association between the breadth of formulary coverage for multiple sclerosis (MS) disease-modifying therapies (DMTs) and MS relapse.

**Design, Setting, and Participants:**

This retrospective cohort study analyzed 100% Medicare administrative data from 2018 to 2022. The data analysis was conducted from August 1, 2024, to January 30, 2025. Beneficiaries with stand-alone prescription drug plans (PDPs) and Medicare Advantage Prescription Drug plans (MA-PDs) were followed for at least 5 quarters (baseline, quarters 1-4; follow-up, quarter 5). Calendar periods reflected formulary decision-making. Beneficiaries in the same Medicare Part D plan during baseline and follow-up, with relapsing-remitting MS, and with MS DMT use during baseline were included.

**Exposures:**

Formulary breadth was low coverage if the 4-quarter moving average of MS DMT drug or class coverage was below the median by plan type and quarter; it was considered high coverage if the average was above the median.

**Main Outcome and Measures:**

The primary outcome was MS relapse, including inpatient or outpatient treatment for MS. Multivariable logistic regressions were estimated separately for PDPs and MA-PDs, controlling for patient and plan characteristics and clustering for repeated observations by beneficiary.

**Results:**

The claims analysis included 50 162 unique beneficiaries in PDPs (mean [SD] age, 58.5 [12.1] years; 74.9% female) and 34 708 in MA-PDs (mean [SD] age, 58.2 [10.3] years; 77.2% female). Oral or injectable MS DMTs were frequently excluded from coverage (>50% excluded in 2022: PDPs, 11 of 15 DMTs; MA-PD, 9 of 15 DMTs). The MS relapse rate was greater for low- vs high-coverage PDPs (10.6% vs 9.5%; odds ratio [OR], 0.88 [95% CI, 0.84-0.92]) and MA-PDs (7.8% vs 6.9; OR, 0.88 [95% CI, 0.85-0.91]). In multivariable analyses, broader formulary coverage during baseline was associated with less MS relapse during follow-up (PDP: adjusted OR, 0.93 [95% CI, 0.90-0.96] for drugs and 0.94 [95% CI, 0.91-0.97] for classes; MA-PD: adjusted OR, 0.88 [95% CI, 0.83-0.94] for drugs and 0.92 [95% CI, 0.86-0.98] for classes).

**Conclusions and Relevance:**

In this cohort study of Medicare data, broader formulary coverage was associated with an 8% to 12% lower odds of MS relapse in MA-PDs and a 6% to 9% lower odds in PDPs. Formulary coverage and restrictions should be tailored for patient need, not just management of costs and use.

## Key Points

**Question:**

Are greater Medicare Part D formulary restrictions associated with worse health outcomes for beneficiaries with relapsing-remitting multiple sclerosis (MS)?

**Findings:**

In this cohort study of 84 870 Medicare beneficiaries with either stand-alone or Medicare Advantage prescription drug plans, broader coverage of disease-modifying therapies in the previous year was significantly associated with fewer MS relapse episodes in the current quarter.

**Meaning:**

These findings suggest that beneficiaries with conditions such as relapsing-remitting MS for which disease-modifying therapies have heterogeneous treatment effects may benefit from increased formulary coverage of pharmaceutical treatments.

## Teaser

This cohort study examines the association between formulary coverage for multiple sclerosis (MS) disease-modifying therapies and MS relapse among Medicare beneficiaries with stand-alone or Medicare Advantage prescription drug plans.

## Introduction

In the US, pharmacy benefit managers use a variety of tools to leverage discounts and rebates from pharmaceutical manufacturers.^1^ These measures include placing medications on preferred or nonpreferred cost-sharing tiers, restricting access with use management requirements, and excluding medicines from formularies altogether. Health plans and pharmacy benefit managers contend that these measures steer beneficiaries to more cost-effective drugs, thereby reducing spending and unnecessary use. However, physician surveys and other evidence suggest that these policies are overused, impose an administrative burden, and undermine clinical decision-making.^2,3^

The steady growth in drug rebates and discounts in recent years is partly due to an increasing number of competing drugs in most therapeutic classes. Within stand-alone prescription drug plans (PDPs) in Medicare Part D, the outpatient prescription drug program for Medicare beneficiaries, manufacturer rebates increased from $27.0 billion in 2016 to $48.6 billion in 2021.^4^ The threat of formulary exclusion gives pharmacy benefit managers leverage to demand lower prices and deeper rebates, as manufacturers want to avoid losing coverage and market access. A recent analysis of Part D found that beneficiaries’ access was restricted through exclusions on an average of 45% of brand-only compounds and 22% of compounds with a generic available.^5^ Formulary exclusions prevent all but the few who successfully appeal or can afford to pay out of pocket from getting the medication if it is prescribed by their physician.

When exclusions are applied to brand drugs with generic equivalents or within classes in which multiple drugs have similar treatment effects, the impact may be as intended (ie, use of more cost-effective or lower-net-cost drugs). However, drugs that treat complex conditions such as cancers and autoimmune disorders, which are often characterized by heterogenous treatment effects across products and beneficiaries, are increasingly subject to formulary restrictions, such as greater cost-sharing or use management, which may be negatively associated with outcomes.^6,7^ When these drugs are in Part D–protected classes, exclusions are limited. Therefore, to assess the appropriateness of formulary exclusions, as opposed to coverage with or without restrictions, for a heterogenous condition, we focused on medications used to treat relapsing-remitting multiple sclerosis (MS), which is not a protected class in Part D.

Relapsing-remitting MS is a good test case because while there are no current cures, an increasing number of disease-modifying therapies (DMTs) are available and have been shown to help slow disease progression, reduce the number of relapses, and limit new disease activity.^8^ Furthermore, current DMTs vary widely by mechanism of action, route of administration, tolerability, and efficacy level; thus, treatment selection is often based on clinical and patient factors and professional judgment.^9,10^ The objectives of this study were to document differences in formulary exclusions of MS DMTs in Medicare Part D and examine whether prior year enrollment in a more restrictive plan was associated with greater odds of MS relapse.

## Methods

### Study Design and Data

This retrospective cohort study used 100% Medicare administrative data for 2018 to 2022, including plan and formulary characteristics, beneficiary enrollment, and medical and pharmacy (Part D) claims.^11,12,13^ This study was reviewed and approved by the University of Southern California Institutional Review Board, which granted a waiver of informed consent and a full waiver of Health Insurance Portability and Accountability Act authorization for performance of research. The study followed the Strengthening the Reporting of Observational Studies in Epidemiology (STROBE) reporting guideline (eTable 1 in Supplement 1).

Medicare beneficiaries may obtain prescription drug coverage through PDPs to supplement fee-for-service Medicare (2018-2022) or by enrolling in a Medicare Advantage (MA) Prescription Drug plan (MA-PD) that combines medical and drug coverage (2018-2021). The incentives for PDPs and MA-PDs differ in that only MA-PDs are at financial risk for both medical and drug expenditures. Beneficiaries were required to have at least 5 quarters of continuous coverage in Parts A and B or Part C and the same Part D plan. The first 4 quarters were used as the baseline period, and the fifth quarter was used as the follow-up period. Each beneficiary could be included as multiple observations in the analysis sample if they had more than 5 quarters of continuous coverage. The study period was defined as each quarter between 2019 quarter 1 (Q1) and 2022 Q4, excluding 2020 Q2 due to the acute health care disruptions caused by the COVID-19 pandemic. The administrative data were supplemented with monthly public use file Part D formularies^14^ and plan enrollment^15^ 2019 to 2022, as well as with drug information (eg, long generic name, date of market entry) from First Databank.

### Beneficiaries With Relapsing-Remitting MS

Beneficiaries were included in the analysis if they had relapsing-remitting MS and baseline MS DMT use (Part B or Part D). Beneficiaries were excluded if they did not have continuous coverage for at least 1 baseline and follow-up period, were in employer-sponsored or Program of All-Inclusive Care for the Elderly plans, had MA in combination with a PDP, died during the quarter, were in the other region (ie, not Northeast, Midwest, South, or West or missing), or were in an MA contract without high data completeness. Beneficiaries with MS were identified using an algorithm developed by Culpepper et al.^16^ Beneficiaries were further limited to those with any MS DMT claims (Part B or Part D) and with relapsing-remitting MS during baseline, also using the Medicare administrative data. Relapsing-remitting MS was defined by the absence of evidence of progressive MS, which was identified by the use of cyclophosphamide, methotrexate, or mitoxantrone hydrochloride or an increase in MS severity during baseline.^17^ Multiple sclerosis severity was measured using the expanded disability status scale derived from the disability/impairments (EDSS-DDI) score (range, 0-10), which measures a patient’s level of functioning and has been translated to *International Classification of Diseases, Ninth Revision, Clinical Modification* codes.^18,19^ Additional details on the calculation of EDSS-DDI scores are available in the eMethods in Supplement 1 and in Supplement 2.

### Innovation and Formulary Restrictions

**Figure 1.**
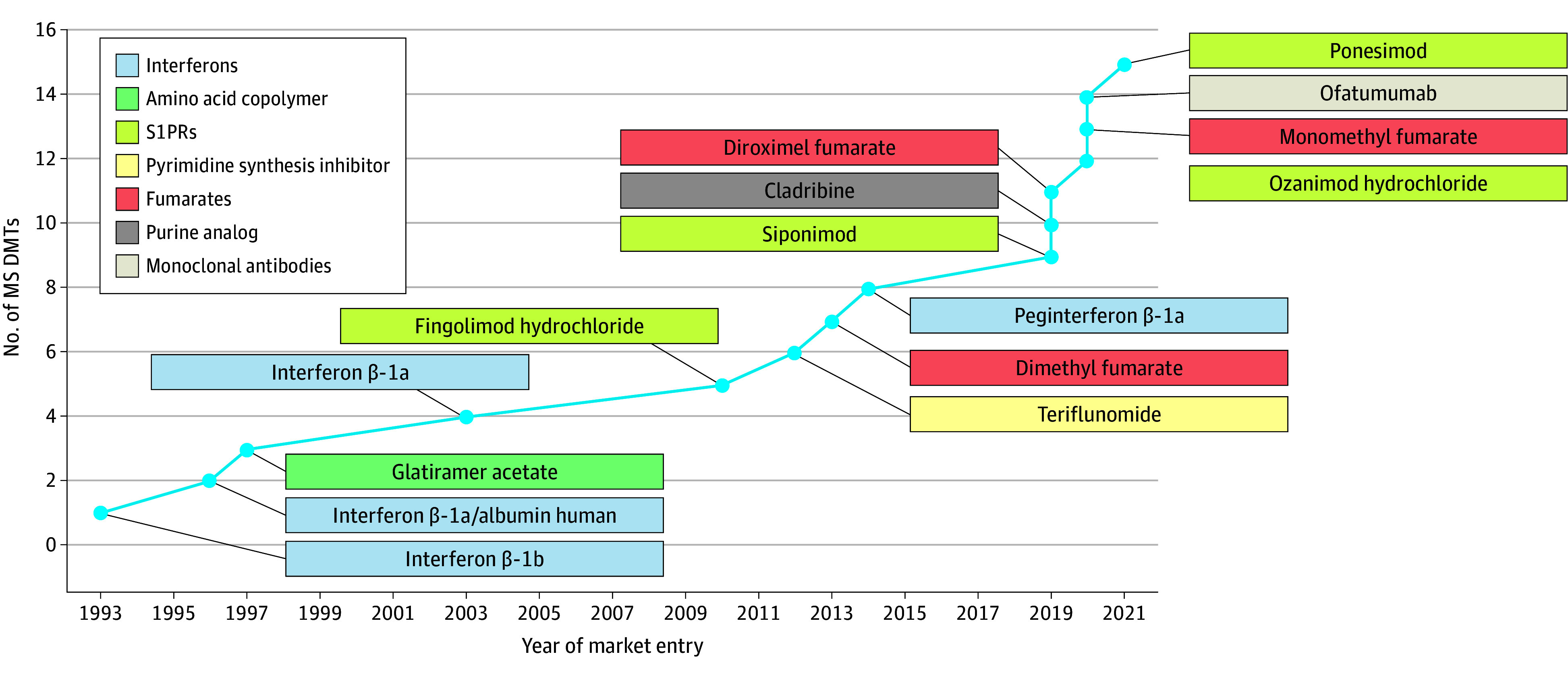
Recent Innovation in Multiple Sclerosis (MS) Disease-Modifying Therapies (DMTs) S1PR indicates sphingosine 1-phosphate receptor modulator.

There has been a good deal of innovation in MS DMTs since 2019 (Figure 1). We examined formulary coverage of the 15 oral and injectable (Part D drugs) DMTs available to beneficiaries with MS quarterly between 2018 and 2022. Multiple sclerosis DMTs were further categorized by therapeutic class (MS drug classes). When oral and injectable drugs are prescribed and filled at a pharmacy, they are reported in the Part D claims and, therefore, subject to formulary restrictions. All plans were assumed to cover the 5 intravenous MS DMTs, commonly administered at a physician’s office and covered under Part B, typically with 20% coinsurance, although supplemental coverage may reduce cost-sharing. Daclizumab was withdrawn from the market in 2018 and was not included in the analysis.

An MS DMT was considered on formulary if any MS-indicated product with the active ingredient (generic name) was covered by the PDP or MA-PD and similarly for MS drug classes. The proportion of MS DMT drugs or classes on formulary (denominator equal to the universe of MS DMT drugs or classes on any Part D formulary in the quarter) for each plan were calculated for the first month of each quarter during the study period. Monthly public use file formularies were only available from April 2019 (2019 Q2; published in January 2019), so the annual formulary for the end of year 2018 was used for 2018 Q1 to 2019 Q1. A 4-quarter moving average of the proportion of drugs on formulary for MS DMT drugs or classes was calculated for each quarter and plan during the study period (eMethods in Supplement 1).

### MS Relapse Episodes, Breadth of Formulary Coverage, and Other Variables

The primary outcome of interest was any MS relapse episode during follow-up. Multiple sclerosis relapses have previously been identified using claims data.^20,21,22^ An MS relapse could be treated in either an inpatient or outpatient setting (eMethods in Supplement 1). Relapses were recorded at the date of the earliest claim, and claims within 30 days of each other were considered a single relapse episode. Secondary outcomes included the number of MS relapse episodes during follow-up, any MS relapse episodes and number of MS relapse episodes by setting (inpatient or outpatient treatment), and any and number of all-cause or MS-related use (eMethods in Supplement 1).

Plans were categorized by plan type (MA-PD vs PDP) and quarter (2019 Q1 to 2022 Q2) as having low coverage of MS DMTs if they covered less than the median of the 4-quarter moving average proportion of MS DMTs during baseline (ie, previous year) among beneficiaries with MS (inclusive of those not using MS DMTs during baseline and/or progressive MS). The binary coverage variable was defined among beneficiaries with MS and by plan type to accommodate beneficiary plan selection (eg, beneficiaries with more active MS may select into plans with broader formulary coverage of DMTs). All other plans were categorized as having high coverage. The categorization was repeated with 3 levels using tertile cutoffs instead of the median (ie, low coverage, moderate coverage, high coverage). Additionally, the same categorizations were repeated for MS DMT classes.

Formulary characteristics for PDPs and MA-PDs also included use management requirements, such as prior authorization or step therapy. A drug was considered under prior authorization or step therapy if any product with that generic name was covered by the plan but every covered product with that generic name required prior authorization and/or step therapy. The proportion of MS DMTs under prior authorization or step therapy (denominator equal to MS DMTs on formulary in the quarter) for each plan was calculated for the first month of each quarter during the study period then combined as a 4-quarter moving average, similar to the calculations for the on-formulary moving average.

To account for variation in overall mortality risk at baseline, we computed the Charlson Comorbidity Index. The Charlson Comorbidity Index score is associated with the risk of mortality.^23,24^ Additional variables included patient demographics (including age, sex, race and ethnicity, and region), original reason for Medicare eligibility (Old-Age and Survivors Insurance vs disability insurance benefits and/or end-stage renal disease), any subsidy in the current quarter (dual eligibility and/or low-income subsidy), and any MS DMT use by route of administration during baseline. Race and ethnicity were reported in the Medicare data as American Indian or Alaska Native, Asian or Pacific Islander, Black or African American, Hispanic, White, other (including any race or ethnicity not listed), or unknown. The American Indian or Alaska Native, Asian or Pacific Islander, other, and unknown categories were combined due to small sample sizes. Race and ethnicity are commonly included in the demographic information in health economic studies when available in the data.

### Statistical Analysis

The data were analyzed between August 1, 2024, and January 30, 2025. To determine the overall formulary coverage of MS DMTs across Part D plans, the proportion of plans without a compound on formulary was calculated by plan type and compound. We also calculated the proportion of plans by count of MS DMT drugs or classes on formulary. The claims analysis of beneficiaries with relapsing-remitting MS and baseline MS DMT use was conducted at the beneficiary-quarter level, so a single beneficiary could have multiple observations in the analysis sample. Descriptive statistics were reported by mean and SD for continuous variables and count and percentage for categorical variables by plan type and formulary coverage (binary or tertiary, drugs or classes). Logistic regression models were estimated for the binary dependent variables of any MS relapse episodes (overall, inpatient treatment, outpatient treatment) during the follow-up period. Poisson or negative binomial regression models were estimated for the nonbinary dependent variables of count of MS relapse episodes (overall, inpatient treatment, outpatient treatment) during the follow-up period. The key independent variables were formulary coverage of MS DMTs (binary or tertiary, drugs or classes) during baseline. Multivariable regression models also controlled for patient characteristics, including age group, sex, race and ethnicity, region, baseline EDSS-DDI score, baseline Charlson Comorbidity Index score, original reason for Medicare eligibility, and moving average proportion of compounds under prior authorization or step therapy during baseline and the study period, in addition to clustering for repeated observations by beneficiary (eMethods in Supplement 1). Missing values were not imputed. In post hoc sensitivity analyses, we repeated the analysis using annual baseline and follow-up periods with formulary coverage from January of the baseline year. The analysis was completed using SAS, version 9.4 (SAS Institute Inc). Statistical significance was tested using 2-sided tests and defined as *P* < .05.

## Results

The claims analysis included 50 162 unique beneficiaries in PDPs (367 833 beneficiary-quarter pairs) and 34 708 unique beneficiaries in MA-PDs (191 329 beneficiary-quarter pairs) (eFigure 1 in Supplement 1). The mean (SD) age of beneficiaries in PDPs was 58.5 (12.1) years; 74.9% were female and 25.1% male; and 12.8% were of Black, 2.6% of Hispanic, 80.7% of White, and 4.0% of other race and ethnicity. Among PDPs, beneficiaries with MS enrolled in high-coverage plans compared with low-coverage plans were older (mean [SD], 60.1 [12.0] vs 57.1 [11.9]; *P* < .001), more likely to be of White race (82.6% vs 78.8%; *P* < .001), initially eligible for Medicare due to age rather than disability (21.3% vs 15.6%; *P* < .001), and less likely to be receiving a subsidy (51.6% vs 67.3%; *P* < .001) (eTable 2 in Supplement 1). The mean (SD) age of beneficiaries in MA-PDs was 58.2 (10.3) years; 77.2% were female and 22.8% male; and 18.6% were of Black, 3.2% of Hispanic, 75.5% of White, and 2.8% of other race and ethnicity. Among MA-PDs, beneficiaries in high-coverage compared with low-coverage plans were more likely to be White (78.5% vs 72.5%; *P* < .001) and reside in the Northeast (24.7% vs 16.0%) and West (24.1% vs 13.0%) (South, 29.7% vs 48.9%; Midwest, 21.6% vs 22.1%) (*P* < .001).

Despite these differences, there was little evidence of plan selection based on MS disease severity (EDSS-DDI) in either PDPs (mean [SD], 2.7 [2.8] vs 2.7 [2.8]; *P* = .39) or MA-PDs (mean [SD], 2.6 [2.7] vs 2.6 [2.7]; *P* = .004). The proportion of drugs under prior authorization or step therapy was highly skewed (ie, median very different from mean) (PDP: mean [SD], 56.1 [44.8] [median (IQR), 75.0 (0.0-100.0)] vs 78.4 [29.7] [median (IQR), 100.0 (66.7-100.0)]; MA-PD: mean [SD], 27.0 [37.8] [median (IQR), 6.3 (0.0-51.7)] vs 85.8 [22.6] [median (IQR), 100.0 (80.0-100.0)]) (both *P* < .001), which did not allow for a binary cutoff. Similar results were observed when formulary coverage was defined based on drug classes and tertiles (eTables 3-5 in Supplement 1).

Oral and injectable MS DMTs had high costs to plans and to beneficiaries (Table 1). Almost all Part D plans covered fingolimod hydrochloride (proportion of plans without compound on formulary: PDP, 3.6%; MA-PD, 0.5%), interferon β-1b (proportion of plans without compound on formulary: PDP, 3.6%; MA-PD, 3.6%), and glatiramer acetate (amino acid copolymer) in 2022. However, many oral or injectable MS DMTs, including older drugs such as teriflunomide, were excluded by almost all PDPs. Mean drug costs and coverage within the analytic sample were similar to the overall Medicare population (>50% excluded in 2022: PDPs, 11 of 15 DMTs; MA-PD, 9 of 15 DMTs) (eTable 6 in Supplement 1).

**Table 1.** Drug Costs and Coverage for Part D MS DMTs by Route of Administration and Market Entry Date^a^

Drug compound	Drug class	Market entry date	Cost per prescription in 2022, mean (SD), $		Plans without compound on formulary (weighted by sample enrollment), %	
			Total	Out of pocket	MA-PD	PDP
**Oral MS DMTs**						
Fingolimod HCL	S1PRs	September 23, 2010	9928 (3299)	72 (315)	0.5	3.6
Teriflunomide	Pyrimidine synthesis inhibitors	September 21, 2012	10 248 (4572)	206 (521)	43.3	87.7
Dimethyl fumarate	Fumarates	March 29, 2013	4990 (4402)	106 (345)	4.2	9.2
Siponimod	S1PRs	March 28, 2019	10 167 (5074)	58 (297)	48.4	96.9
Cladribine	Purine analogs	April 2, 2019	71 962 (18 400)	506 (1453)	85.1	98.5
Diroximel fumarate	Fumarates	November 1, 2019	9258 (4086)	191 (483)	79.5	80.6
Ozanimod HCL	S1PRs	May 26, 2020	9801 (5068)	122 (442)	86.9	99.7
Monomethyl fumarate	Fumarates	August 4, 2020	8324 (4676)	172 (384)	91.1	99.7
Ponesimod	S1PRs	March 18, 2021	10 717 (6625)	210 (477)	100.0	99.7
**Injectable DMTs**						
Interferon β-1B	Interferons	September 9, 1993	8932 (1906)	177 (452)	2.0	0.0
Interferon β-1A/albumin human	Interferons	May 23, 1996	10 104 (3687)	310 (601)	41.4	86.5
Glatiramer acetate	Amino acid copolymers	March 11, 1997	5136 (2498)	235 (438)	0.0	0.0
Interferon β-1A	Interferons	July 28, 2003	8435 (2915)	246 (525)	33.5	85.0
Peginterferon β-1A	Interferons	October 6, 2014	9253 (4542)	179 (496)	72.0	97.9
Ofatumumab	Monoclonal antibodies	August 24, 2020	9718 (5024)	56 (278)	86.0	99.6

Abbreviations: HCL, hydrochloride; MA-PD, Medicare Advantage Prescription Drug plan; PDP, stand-alone prescription drug plan; S1PR, sphingosine 1-phosphate receptor modulator.

^a^ Coverage information from public use file formularies representative of January 2022 for PDPs and October 2021 for MA-PDs.

**Figure 2.**
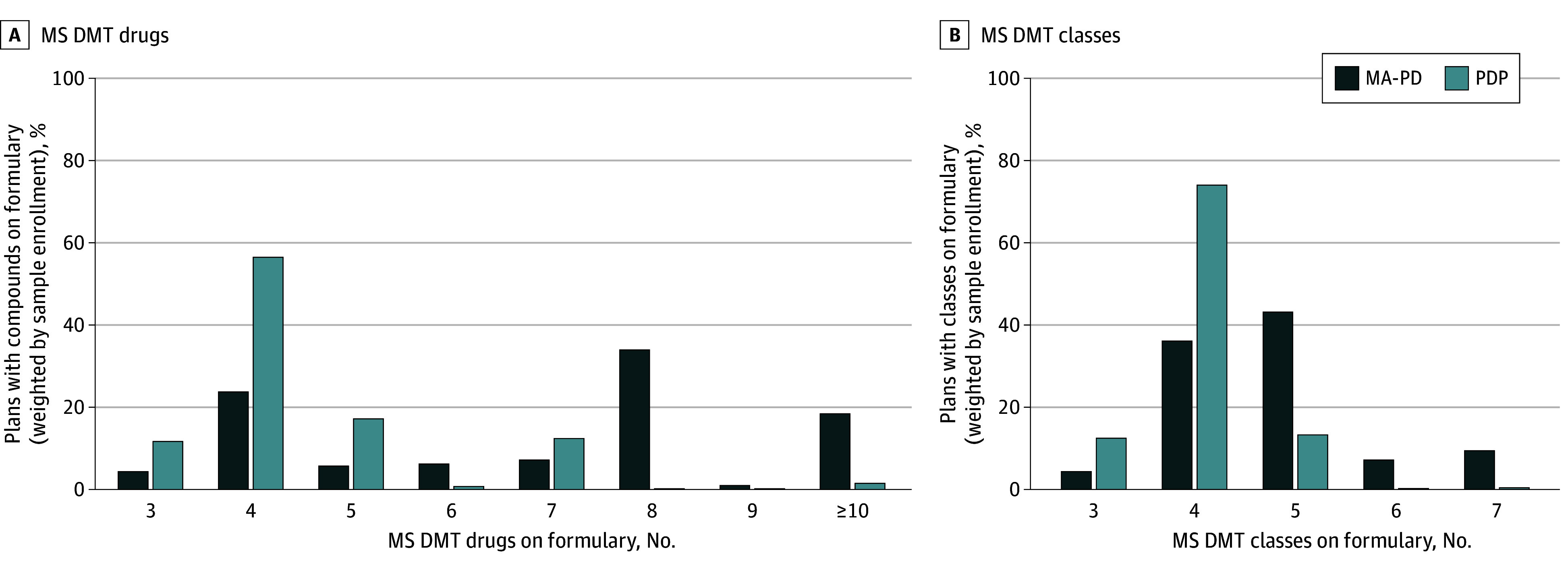
Coverage of Multiple Sclerosis (MS) Disease-Modifying Therapy (DMT) Drugs and Classes on Part D Formularies The maximum number of compounds in 2022 was 15. Coverage information from public use file formularies representative of January 2022 for stand-alone prescription drug plans (PDPs) and October 2021 for Medicare Advantage Prescription Drug plans (MA-PDs).

Prescription drug plans most frequently included 26.7% of available MS DMT drugs (4 of 15) on their formularies or 57.1% of available MS DMT classes (4 of 7) in 2022 (Figure 2). Medicare Advantage Prescription Drug plan coverage of MS DMTs in 2022 was more extensive, with the greatest proportion of plans covering 53.3% of MS DMT drugs (8 of 15) or 71.4% of MS DMT classes (5 of 7). Beneficiaries with MS did not appear to select into Part D plans with broader coverage compared with the overall Medicare population in 2022 (eFigure 2 in Supplement 1). This finding was similar when plans were not weighted by enrollment (eFigure 3 in Supplement 1) and there was a decrease in formulary coverage as a proportion of available MS DMT drugs or classes from 2019 (greatest proportion of plans included: PDP, 37.5% of drugs [3 of 8] and 60.0% of classes [3 of 5]; MA-PD, 87.5% of drugs [7 of 8] and 100% of classes [5 of 5]) (eFigure 4 in Supplement 1).

**Figure 3.**
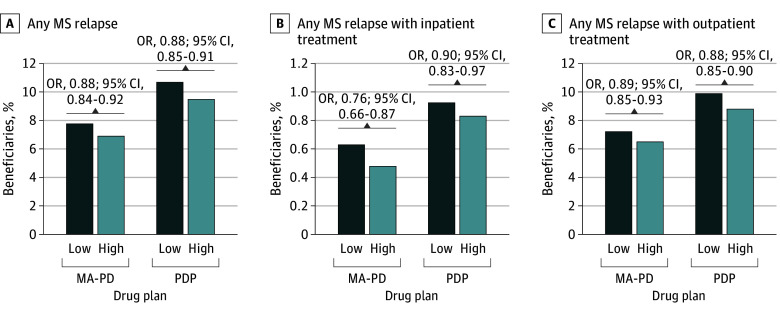
Rates of Any Multiple Sclerosis (MS) Relapse Low and high formulary coverage defined for a qualified MS population by quarter and plan type using median 4-quarter moving average proportion of drugs on formulary. Unadjusted odds ratios (ORs) were estimated using univariable logistic regression with clustering for repeated observations by beneficiary (reference, high formulary coverage). MA-PD indicates Medicare Advantage Prescription Drug plan; PDP, stand-alone prescription drug plan.

The rate of any MS relapse was greater for low- compared with high-coverage plans for both PDPs (10.6% vs 9.5%; odds ratio [OR], 0.88 [95% CI, 0.85-0.91]) and MA-PDs (7.8% vs 6.9%; OR, 0.88 [95% CI, 0.84-0.92]) (Figure 3). After adjusting for patient and plan characteristics, high-coverage plans were still associated with lower relapse rates for PDPs (adjusted OR, 0.93 [95% CI, 0.90-0.96]) and MA-PD (adjusted OR, 0.88 [95% CI, 0.83-0.94]), driven by the top third vs the lowest third of MS DMT breadth of coverage (Table 2). However, the results were less robust for relapses treated in an inpatient setting for both PDPs and MA-PDs. Complete multivariable regression models are reported in eTables 7 to 10 in Supplement 1. Results for count of MS relapses and sensitivity analyses produced similar associations (eTables 11 and 12 in Supplement 1). Fewer significant results were reported for all-cause and MS-related use but indicated fewer emergency department and more office visits with high coverage (eTables 13-16 in Supplement 1).

**Table 2.** Logistic Regressions of Any MS Relapse During Follow-Up Quarters

Formulary coverage^a^	MA-PD (2019 Q1 to 2021 Q4)						PDP (2019 Q1 to 2022 Q4)					
	Any MS relapse		Any MS relapse with inpatient treatment		Any MS relapse with outpatient treatment		Any MS relapse		Any MS relapse with inpatient treatment		Any MS relapse with outpatient treatment	
	AOR (95% CI)^b^	*P* value	AOR (95% CI)^b^	*P* value	AOR (95% CI)^b^	*P* value	AOR (95% CI)^b^	*P* value	AOR (95% CI)^b^	*P* value	AOR (95% CI)^b^	*P* value
**Defined by drugs**												
Binary formulary coverage (reference = low)												
High	0.88 (0.83-0.94)	<.001	0.82 (0.68-1.00)	.04	0.89 (0.83-0.95)	<.001	0.93 (0.90-0.96)	<.001	1.02 (0.94-1.11)	.70	0.92 (0.89-0.95)	<.001
Tertiary formulary coverage (reference = low)												
Moderate	0.93 (0.87-1.00)	.05	0.98 (0.80-1.20)	.83	0.93 (0.87-1.00)	.06	0.98 (0.94-1.02)	.29	1.05 (0.95-1.17)	.31	0.97 (0.93-1.01)	.13
High	0.88 (0.82-0.95)	<.001	0.67 (0.53-0.85)	<.001	0.90 (0.83-0.97)	.004	0.92 (0.88-0.95)	<.001	1.02 (0.92-1.13)	.72	0.90 (0.87-0.94)	<.001
**Defined by drug class**												
Binary formulary coverage (reference = low)												
High	0.92 (0.86-0.98)	.006	1.01 (0.83-1.22)	.93	0.91 (0.86-0.98)	.006	0.94 (0.91-0.97)	<.001	1.07 (0.97-1.17)	.17	0.93 (0.89-0.96)	<.001
Tertiary formulary coverage (reference = low)												
Moderate	0.99 (0.92-1.06)	.77	0.90 (0.72-1.11)	.31	1.00 (0.93-1.07)	.97	1.00 (0.96-1.04)	.98	1.10 (0.98-1.23)	.10	0.99 (0.95-1.03)	.61
High	0.91 (0.85-0.97)	.006	0.96 (0.77-1.19)	.70	0.91 (0.84-0.97)	.007	0.91 (0.87-0.95)	<.001	1.02 (0.91-1.14)	.78	0.90 (0.86-0.94)	<.001

Abbreviations: AOR, adjusted odds ratio; MA-PD, Medicare Advantage Prescription Drug plan; MS, multiple sclerosis; PDP, stand-alone Prescription Drug Plan; Q, quarter.

^a^ Formulary coverage defined for qualified MS population by quarter and plan type using median 4-quarter moving average proportion of drugs or classes on formulary. Observations for 2020 Q2 were dropped from the analysis.

^b^ Adjusted odds ratios estimated using multivariable logistic regression controlling for binary or tertiary formulary coverage, age group, sex, race and ethnicity, region, baseline expanded disability status scale disability-derived impairments score, baseline Charlson Comorbidity Index score, original reason for Medicare eligibility, moving average proportion of compounds under prior authorization or step therapy during baseline, and study period in addition to clustering for repeated observations by beneficiary. Race and ethnicity were reported in the Medicare data as American Indian or Alaska Native, Asian or Pacific Islander, Black, Hispanic, White, other (including any race and ethnicity not listed), or unknown. The American Indian or Alaska Native, Asian or Pacific Islander, other, and unknown categories were combined due to small sample sizes.

## Discussion

The objectives of this cohort study were to document differences in formulary exclusions of MS DMTs and examine whether enrollment in a more restrictive plan was associated with MS relapse. As the number of MS DMTs has increased, formulary coverage has become more restrictive in both PDPs and MA-PDs. We found a negative association between formulary coverage of MS DMTs and the odds of MS relapse among Medicare beneficiaries. Broader formulary coverage in MA-PDs was associated with an 8% to 12% lower odds of MS relapse during follow-up and a 6% to 9% lower odds in PDPs.

An earlier study of Part D formularies reported decreasing coverage for MS DMTs from 2007 to 2016.^25^ Since that time, the drug landscape for MS treatment has changed with the introduction of multiple new therapies. Unfortunately, high levels of formulary exclusion and restrictions have been increasingly common among new drugs in nonprotected classes (ie, exclusive of immunosuppressants for prophylaxis of organ transplant rejection, antidepressants, antipsychotics, anticonvulsants, antiretrovirals, and antineoplastics).^26^ Multiple sclerosis DMTs are no exception, and despite innovation in MS therapies, many are not easily accessible by Part D beneficiaries due to formulary exclusions. This trend may be exacerbated by the newly enacted $2000 annual cap on out-of-pocket drug costs in Part D. The cap increases plan liability above the threshold and restricts its ability to steer beneficiaries toward lower-cost treatments and may lead to narrower coverage of high-cost therapies given that only covered drugs count toward the out-of-pocket cap.

Prior studies have reported mixed results on the association between formulary restrictions and health outcomes.^27,28,29,30,31,32^ When covered by Part D formularies, MS DMTs are commonly on the specialty drug formulary tier (Tier 5), which typically has greater cost-sharing and prior authorization requirements.^33^ Greater prior authorization requirements for specialty drugs have been associated with delayed initiation.^34^ Greater cost-sharing has been associated with worse medication adherence for MS DMTs; specialty pharmacy programs have been associated with greater adherence.^35^ Persistence to MS DMTs is varied and concerningly low, often associated with suboptimal response, side effects, or formulary changes.^36^ The current study suggests that narrow formulary coverage of heterogeneous MS treatments may be associated with worse health outcomes.

### Strengths and Limitations

This study benefits from use of 100% Medicare administrative data, which allow for a large number of beneficiaries with relapsing-remitting MS. However, the study is subject to some limitations. Because the age of onset of MS is much younger than 65 years (ie, typical entitlement age of Medicare),^37^ we were not able to identify beneficiaries at the first initiation of MS treatment. We controlled for age group and original reason for Medicare entitlement as a proxy for length of time with MS. Furthermore, specific diagnosis codes for relapsing-remitting MS or MS relapse were not available, so algorithms adapted for administrative claims data were used. As is common among retrospective studies using health care claims data, the data collected for administrative billing purposes may contain errors or omissions. Although we did not find a strong indicator of Part D plan selection based on MS disease severity, Medicare beneficiaries may choose Part D plans based, in part, on unobserved characteristics. Most patients with an MS relapse were treated in the outpatient setting, which limited the robustness of the results for inpatient relapses alone. Finally, the quarterly assessment of outcomes may be too short a period to see an association between previous year formulary coverage and MS relapse. Similar associations were found with a longer 1-year follow-up period.

## Conclusions

In this cohort study of Medicare data, formularies excluded a greater proportion of available MS DMTs over time in both PDPs and MA-PDs, and more restrictive formularies were associated with greater MS relapse. Health plans and pharmacy benefit managers have been relying on formulary exclusion to limit costs of new therapies. However, a more nuanced approach that also accounts for patient benefit (and potential harm) should also be considered. Creative methods of financing drug costs have been discussed for curative therapies but could also be considered for DMTs.

## Notes

Supplement 1.
eMethods.

eReferences.
**eTable 1.** STROBE Statement **eFigure 1.** Flow Diagram **eTable 2.** Patient Characteristics by Play Type and MS DMT Formulary Coverage: Binary Coverage Based on MS DMT Drugs **eTable 3.** Patient Characteristics by Plan Type and MS DMT Formulary Coverage: Binary Coverage Based on MS DMT Classes **eTable 4.** Patient Characteristics by Plan Type and MS DMT Formulary Coverage: Tertiary Coverage Based on MS DMT Drugs **eTable 5.** Patient Characteristics by Plan Type and MS DMT Formulary Coverage: Tertiary Coverage Based on MS DMT Classes **eTable 6.** Drug Costs and Coverage for Part D MS DMTs, 2022 (Sorted by Route of Administration and Date Entered Market) **eFigure 2.** Coverage of MS DMT Drugs and Classes on Part D Formularies, All Medicare, 2022 **eFigure 3.** Coverage of MS DMT Drugs and Classes on Part D Formularies, Unweighted, 2022 **eFigure 4.** Coverage of MS DMT Drugs and Classes on Part D Formularies, RRMS Sample, 2019 **eTable 7.** Multivariable Logistic Regressions of Any MS Relapse (Overall, Inpatient Treatment, Outpatient Treatment) During Follow-Up Quarter: Binary Coverage Based on MS DMT Drugs **eTable 8.** Multivariable Logistic Regressions of Any MS Relapse (Overall, Inpatient Treatment, Outpatient Treatment) During Follow-Up Quarter: Binary Coverage Based on MS DMT Classes **eTable 9.** Multivariable Logistic Regressions of Any MS Relapse (Overall, Inpatient Treatment, Outpatient Treatment) During Follow-Up Quarter: Tertiary Coverage Based on MS DMT Drugs **eTable 10.** Multivariable Logistic Regressions of Any MS Relapse (Overall, Inpatient Treatment, Outpatient Treatment) During Follow-Up Quarter: Tertiary Coverage Based on MS DMT Classes **eTable 11.** Regressions of Count of MS Relapse (Overall, Inpatient, Outpatient) During Follow-Up Quarter **eTable 12.** Regressions of Any or Count of MS Relapse (Overall, Inpatient, Outpatient) During Follow-Up Year in Sensitivity Analysis Sample **eTable 13.** Regressions of Any All-Cause Utilization (Inpatient, ED, Office Visits) During Follow-Up Quarter **eTable 14.** Regressions of Count of All-Cause Utilization (Inpatient, ED, Office Visits) During Follow-Up Quarter **eTable 15.** Regressions of Any MS-Related Utilization (Inpatient, ED, Office Visits) During Follow-Up Quarter **eTable 16.** Regressions of Count of MS-Related Utilization (Inpatient, ED, Office Visits) During Follow-Up Quarter

Supplement 2.
SAS Code for the EDSS-DDI Score

Supplement 3.
Data Sharing Statement
