# Supplementary material for: Formulary Restrictions and Relapse Episodes in Persons With Relapsing-Remitting Multiple Sclerosis
Source: JAMA Netw Open. 2025 Aug 1;8(8):e2525155. doi: 10.1001/jamanetworkopen.2025.25155 (PMC12317357; doi:10.1001/jamanetworkopen.2025.25155)
Supplement: Supplement 1. — eMethods. eReferences. eTable 1. STROBE Statement eFigure 1. Flow Diagram eTable 2. Patient Characteristics by Play Type and MS DMT Formulary Coverage: Binary Coverage Based on MS DMT Drugs eTable 3. Patient Characteristics by Plan Type and MS DMT Formulary Coverage: Binary Coverage Based on MS DMT Classes eTable 4. Patient Characteristics by Plan Type and MS DMT Formulary Coverage: Tertiary Coverage Based on MS DMT Drugs eTable 5. Patient Characteristics by Plan Type and MS DMT Formulary Coverage: Tertiary Coverage Based on MS DMT Classes eTable 6. Drug Costs and Coverage for Part D MS DMTs, 2022 (Sorted by Route of Administration and Date Entered Market) eFigure 2. Coverage of MS DMT Drugs and Classes on Part D Formularies, All Medicare, 2022 eFigure 3. Coverage of MS DMT Drugs and Classes on Part D Formularies, Unweighted, 2022 eFigure 4. Coverage of MS DMT Drugs and Classes on Part D Formularies, RRMS Sample, 2019 eTable 7. Multivariable Logistic Regressions of Any MS Relapse (Overall, Inpatient Treatment, Outpatient Treatment) During Follow-Up Quarter: Binary Coverage Based on MS DMT Drugs eTable 8. Multivariable Logistic Regressions of Any MS Relapse (Overall, Inpatient Treatment, Outpatient Treatment) During Follow-Up Quarter: Binary Coverage Based on MS DMT Classes eTable 9. Multivariable Logistic Regressions of Any MS Relapse (Overall, Inpatient Treatment, Outpatient Treatment) During Follow-Up Quarter: Tertiary Coverage Based on MS DMT Drugs eTable 10. Multivariable Logistic Regressions of Any MS Relapse (Overall, Inpatient Treatment, Outpatient Treatment) During Follow-Up Quarter: Tertiary Coverage Based on MS DMT Classes eTable 11. Regressions of Count of MS Relapse (Overall, Inpatient, Outpatient) During Follow-Up Quarter eTable 12. Regressions of Any or Count of MS Relapse (Overall, Inpatient, Outpatient) During Follow-Up Year in Sensitivity Analysis Sample eTable 13. Regressions of Any All-Cause Utilization (Inpatient, ED, Office Visits) During Follow [file jamanetwopen-e2525155-s001.pdf]

Supplemental Online Content

Blaylock B, Van Nuys K, Joyce G. Formulary restrictions and relapse episodes in persons with relapsing-remitting multiple sclerosis. *JAMA Netw Open*. 202;8(8):e2525155.
doi:10.1001/jamanetworkopen.2025.25155

eMethods

eReferences

eTable 1. STROBE Statement

eFigure 1. Flow Diagram

eTable 2. Patient Characteristics by Play Type and MS DMT Formulary Coverage: Binary Coverage Based on MS DMT Drugs

eTable 3. Patient Characteristics by Plan Type and MS DMT Formulary Coverage: Binary Coverage Based on MS DMT Classes

eTable 4. Patient Characteristics by Plan Type and MS DMT Formulary Coverage: Tertiary Coverage Based on MS DMT Drugs

eTable 5. Patient Characteristics by Plan Type and MS DMT Formulary Coverage: Tertiary Coverage Based on MS DMT Classes

eTable 6. Drug Costs and Coverage for Part D MS DMTs, 2022 (Sorted by Route of Administration and Date Entered Market)

eFigure 2. Coverage of MS DMT Drugs and Classes on Part D Formularies, All Medicare, 2022

eFigure 3. Coverage of MS DMT Drugs and Classes on Part D Formularies, Unweighted, 2022

eFigure 4. Coverage of MS DMT Drugs and Classes on Part D Formularies, RRMS Sample, 2019

eTable 7. Multivariable Logistic Regressions of Any MS Relapse (Overall, Inpatient Treatment, Outpatient Treatment) During Follow-Up Quarter: Binary Coverage Based on MS DMT Drugs

eTable 8. Multivariable Logistic Regressions of Any MS Relapse (Overall, Inpatient Treatment, Outpatient Treatment) During Follow-Up Quarter: Binary Coverage Based on MS DMT Classes

eTable 9. Multivariable Logistic Regressions of Any MS Relapse (Overall, Inpatient Treatment, Outpatient Treatment) During Follow-Up Quarter: Tertiary Coverage Based on MS DMT Drugs

eTable 10. Multivariable Logistic Regressions of Any MS Relapse (Overall, Inpatient Treatment, Outpatient Treatment) During Follow-Up Quarter: Tertiary Coverage Based on MS DMT Classes

eTable 11. Regressions of Count of MS Relapse (Overall, Inpatient, Outpatient) During Follow-Up Quarter

eTable 12. Regressions of Any or Count of MS Relapse (Overall, Inpatient, Outpatient) During Follow-Up Year in Sensitivity Analysis Sample

eTable 13. Regressions of Any All-Cause Utilization (Inpatient, ED, Office Visits) During Follow-Up Quarter

© 2025 Blaylock B et al. *JAMA Network Open*.

eTable 14. Regressions of Count of All-Cause Utilization (Inpatient, ED, Office Visits) During Follow-Up Quarter

eTable 15. Regressions of Any MS-Related Utilization (Inpatient, ED, Office Visits) During Follow-Up Quarter

eTable 16. Regressions of Count of MS-Related Utilization (Inpatient, ED, Office Visits) During Follow-Up Quarter

This supplemental material has been provided by the authors to give readers additional information about their work.

eMethods

Plans included traditional fee-for-service (FFS) Medicare (available through 2022) as well as Medicare Advantage (MA) plans determined to have 'high data completeness' (available through 2021). In contrast to FFS Medicare, MA plans are capitated and thus have different reporting requirements for patient encounters. We adapted methods from Jung et al (2022),[1] which processed and reformatted encounters to resemble claims data, to identify MA contracts with 'high data completeness' based on concordance with the Medicare Provider Analysis and Review inpatient hospitalizations and restricted the sample to those MA plans.

In special cases, a beneficiary in a MA plan without drug benefits may also be enrolled in a PDP; those individuals were removed from the analysis. The monthly public use file formularies exclude employer-sponsored or Program of All-inclusive Care for the Elderly Part D plans, so beneficiaries in employer-sponsored or Program of All-inclusive Care for the Elderly plans were removed from the analysis.

Multiple sclerosis (MS) disease modifying therapies (DMTs) typically covered by Medicare Part D included *fumarates* (dimethyl fumarate, diroximel fumarate, monomethyl fumarate), *interferons* (interferon beta-1A, interferon beta-1A/albumin human, interferon beta-1B, peginterferon beta-1A), *monoclonal antibodies* (ofatumumab), *sphingosine-1-phosphate receptors* (S1PRs; fingolimod hydrochloride, ozanimod hydrochloride, siponimod, ponesimod), *amino acid copolymer* (glatiramer acetate), *pyrimidine synthesis inhibitor* (teriflunomide), and *purine analog* (cladribine). Intravenous MS DMTs typically covered by Part B included *monoclonal antibodies* (alemtuzumab, natalizumab, ocrelizumab, ublituximab-xiiy) and a *topoisomerase II inhibitor* (mitoxantrone hydrochloride).

The 4-quarter moving averages were calculated as illustrated below using the proportion of MS DMT drugs in 2021 as an example.

	2021Q1	2021Q2	2021Q3	2021Q4	4-quarter moving average
Number of MS DMTs on any Part D formulary, as of first month of quarter	11	13	14	14	
Number of MS DMTs on example Part D formulary, as of first month of quarter	7	7	7	7	
Proportion of MS DMTs on example Part D formulary, as of first month of quarter	63.6%	53.8%	50.0%	50.0%	54.4%

Beneficiaries with any combination of three or more inpatient and/or outpatient claims with a diagnosis of MS (ICD10=G35) or for a MS DMT (Part B or Part D) during the 1-year baseline period in the Medicare administrative data were eligible for the analysis. Past analyses of beneficiaries with MS in the Medicare data have used the same algorithm.[2] MS DMT medical claims (Part B) were required to have a diagnosis of MS on the same claim to avoid flagging drug use for other conditions (e.g., cancer).

We further translated the ICD-9-CM codes to ICD-10-CM codes (see EDSS-DDI scoring macro supplement). EDSS-DDI scores were calculated for the full 1-year baseline period as well as for the first and second six months. The six-month scores were used to define an increase in MS severity during baseline (increase of ≥1.0 if the EDSS-DDI score in the first six months was [0-5.5) or an increase of ≥0.5 if the previous EDSS-DDI score in the first six months was ≥5.5).[3] If a beneficiary was missing an EDSS-DDI score in either 6-month period and did not have a claim for cyclophosphamide, methotrexate, or mitoxantrone hydrochloride during baseline, they were categorized as RRMS. The National Cancer Institute (NCI) Comorbidity SAS Macro (Version 2021) was used to calculate the CCI score from diagnoses on inpatient, outpatient, and carrier claims.[4]

Inpatient treatment of MS relapse was defined by MS-related hospitalizations (diagnosis related group=058, 059, 060). Diagnosis related groups were used because they were available in both the FFS claims and MA encounters data. Outpatient treatment of MS relapse was defined as an outpatient visit with an MS diagnosis code in any position followed by treatment with a corticosteroid (methylprednisolone, dexamethasone, prednisone,

© 2025 Blaylock B et al. *JAMA Network Open*.

betamethasone, prednisolone, triamcinolone) within 7 days and/or treatment with plasmapheresis, intravenous immunoglobulin, or repository corticotropin injection within 30 days.

Other utilization included all-cause and MS-related inpatient stays, emergency department (ED) visits, and office visits. MS-related stays or visits were defined by a diagnosis code for MS on the claim, in any position (ICD10=G35). ED visits were defined by Medicare revenue center codes (0450-9, 0981). Office visits were defined by Healthcare Common Procedure Coding System (HCPCS) codes (95115, 95117, 99058, 99201-5, 99211-5,99354-5, 99366-7, 99381-7, 99391-7, 99401-4, 99412, 99420, 99429, G0101, G0245-8, G0250, G0402, G0420-1, G0463, G0466-70, G0473, G2011, G2083, G9980) associated with Berenson-Eggers Type of Service (BETOS) codes M1A Office visits - new or M1B Office visits - established.[5]

Race/ethnicity was reported in the Medicare data as Non-Hispanic White, Black (Or African-American), Asian/Pacific Islander, Hispanic, American Indian/Alaska Native, Other, or Unknown. The categories for Asian/Pacific Islander, American Indian/Alaska Native, Other, or Unknown were combined due to small sample size.

Clustering for repeated observations by beneficiary used a generalized estimating equations model with an independent working correlation matrix, the SAS default. No mathematical transformations of the dependent or independent variables were used. Any subsidy was excluded from the final models due to high association with the original reason for Medicare eligibility.

Unobserved socioeconomic factors could be associated with Part D plan choice and may also be associated with MS relapse. We limited the dependent variable to MS relapse and controlled for disease severity, rather than also modeling disease severity or time between relapses, which would have added a large amount of complexity to the analysis. We did not analyze the association between coverage of specific MS DMTs alone or in combination and MS relapse, which would have also added a large amount of complexity to the analysis due, in part, to the changing availability of drugs over time and drug switching by beneficiaries. Similarly, intravenous MS DMTs were a changing proportion of available MS DMTs over time, which was not considered in the analysis. The results may not be generalizable to a commercially insured population, which would likely be younger with less severe MS. Due to data access limitations, formulary coverage for oral and injectable MS DMTs was assumed not to change between 2018Q1-2019Q1. Recent innovations in MS DMTs occurred after the beginning of 2019, which are captured in the analysis of monthly formularies.

eReferences

1. Jung J, Carlin C, Feldman R, Tran L. Implementation of resource use measures in Medicare Advantage. *Health Serv Res.* 2022;57(4):957-962.

2. Hartung DM, Johnston KA, McGregor JC, Bourdette DN. Association between pharmacy benefit restrictions and disease-modifying therapy use in the Medicare Part D program. *Neurol Clin Pract.* 2022;12(1):36-42.

3. Van Le H, Le Truong CT, Kamauu AWC, et al. Identifying patients with relapsing-remitting multiple sclerosis using algorithms applied to US integrated delivery network healthcare data. *Value Health.* 2019;22(1):77-84.

4. National Cancer Institute (NCI). Comorbidity SAS Macro (2021 version). https://healthcaredelivery.cancer.gov/seermedicare/considerations/macro-2021.html. Published 2021.

5. Centers for Medicare and Medicaid Services (CMS). Restructured BETOS classification system crosswalk (2022 version). https://data.cms.gov/sites/default/files/2022-10/2022%20RBCS%20and%20BETOS%20Crosswalk.xlsx. Accessed 30 May 2025.

© 2025 Blaylock B et al. *JAMA Network Open*.

eTable 1. STROBE Statement

	Item No	Recommendation	Page No
**Title and abstract**	1	(*a*) Indicate the study's design with a commonly used term in the title or the abstract	1
		(*b*) Provide in the abstract an informative and balanced summary of what was done and what was found	5
**Introduction**			
Background/rationale	2	Explain the scientific background and rationale for the investigation being reported	7
Objectives	3	State specific objectives, including any prespecified hypotheses	8
**Methods**			
Study design	4	Present key elements of study design early in the paper	
Setting	5	Describe the setting, locations, and relevant dates, including periods of recruitment, exposure, follow-up, and data collection	8
Participants	6	(*a*) *Cohort study*—Give the eligibility criteria, and the sources and methods of selection of participants. Describe methods of follow-up<br>*Case-control study*—Give the eligibility criteria, and the sources and methods of case ascertainment and control selection. Give the rationale for the choice of cases and controls<br>*Cross-sectional study*—Give the eligibility criteria, and the sources and methods of selection of participants	9
		(*b*) *Cohort study*—For matched studies, give matching criteria and number of exposed and unexposed<br>*Case-control study*—For matched studies, give matching criteria and the number of controls per case	NA
Variables	7	Clearly define all outcomes, exposures, predictors, potential confounders, and effect modifiers. Give diagnostic criteria, if applicable	11
Data sources/ measurement	8*	For each variable of interest, give sources of data and details of methods of assessment (measurement). Describe comparability of assessment methods if there is more than one group	11
Bias	9	Describe any efforts to address potential sources of bias	17
Study size	10	Explain how the study size was arrived at	9
Quantitative variables	11	Explain how quantitative variables were handled in the analyses. If applicable, describe which groupings were chosen and why	12
Statistical methods	12	(*a*) Describe all statistical methods, including those used to control for confounding	13
		(*b*) Describe any methods used to examine subgroups and interactions	13
		(*c*) Explain how missing data were addressed	13
		(*d*) *Cohort study*—If applicable, explain how loss to follow-up was addressed<br>*Case-control study*—If applicable, explain how matching of cases and controls was addressed	NA

© 2025 Blaylock B et al. *JAMA Network Open*.

	Item No	Recommendation	Page No
		Cross-sectional study—If applicable, describe analytical methods taking account of sampling strategy	
		(*e*) Describe any sensitivity analyses	13
**Results**			
Participants	13*	(a) Report numbers of individuals at each stage of study—eg numbers potentially eligible, examined for eligibility, confirmed eligible, included in the study, completing follow-up, and analysed	13
		(b) Give reasons for non-participation at each stage	eFigure1
		(c) Consider use of a flow diagram	eFigure1
Descriptive data	14*	(a) Give characteristics of study participants (eg demographic, clinical, social) and information on exposures and potential confounders	13
		(b) Indicate number of participants with missing data for each variable of interest	eFigure1
		(c) *Cohort study*—Summarise follow-up time (eg, average and total amount)	13
Outcome data	15*	*Cohort study*—Report numbers of outcome events or summary measures over time	15
		Case-control study—Report numbers in each exposure category, or summary measures of exposure	eTable 2
		Cross-sectional study—Report numbers of outcome events or summary measures	eTable 2
Main results	16	(*a*) Give unadjusted estimates and, if applicable, confounder-adjusted estimates and their precision (eg, 95% confidence interval). Make clear which confounders were adjusted for and why they were included	15
		(*b*) Report category boundaries when continuous variables were categorized	eTable 2
		(*c*) If relevant, consider translating estimates of relative risk into absolute risk for a meaningful time period	eTable 2
Other analyses	17	Report other analyses done—eg analyses of subgroups and interactions, and sensitivity analyses	15
**Discussion**			
Key results	18	Summarise key results with reference to study objectives	15
Limitations	19	Discuss limitations of the study, taking into account sources of potential bias or imprecision. Discuss both direction and magnitude of any potential bias	17
Interpretation	20	Give a cautious overall interpretation of results considering objectives, limitations, multiplicity of analyses, results from similar studies, and other relevant evidence	18
Generalisability	21	Discuss the generalisability (external validity) of the study results	18
**Other information**			
Funding	22	Give the source of funding and the role of the funders for the present study and, if applicable, for the original study on which the present article is based	2

*Give information separately for cases and controls in case-control studies and, if applicable, for exposed and unexposed groups in cohort and cross-sectional studies.

© 2025 Blaylock B et al. *JAMA Network Open*.

eFigure 1. Flow Diagram

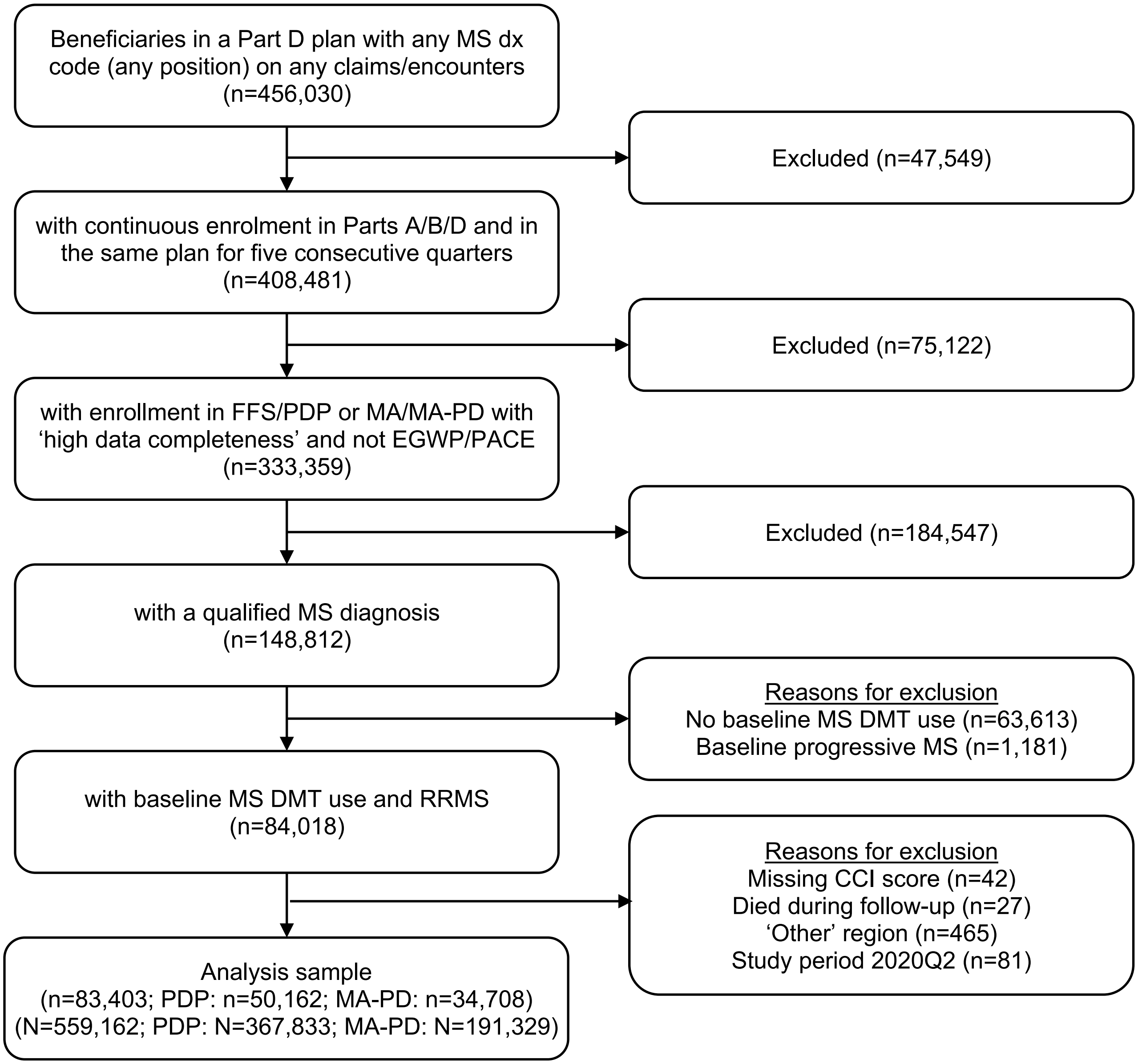

Abbreviations: CCI, Charlson Comorbidity Index; DMT, disease modifying therapy; EGWP, employer group waiver plan; EOY, end of year; FFS, fee-for-service; MA, Medicare Advantage; MA-PD, Medicare Advantage Prescription Drug plan; MS, multiple sclerosis; n, number of beneficiaries; N, number of beneficiary-quarter pairs; PACE, Program of All-inclusive Care for the Elderly; PDP, stand-alone Prescription Drug Plan; RRMS, relapsing-remitting multiple sclerosis.

© 2025 Blaylock B et al. *JAMA Network Open*.

eTable 2. Patient Characteristics by Plan Type and MS DMT Formulary Coverage: Binary Coverage Based on MS DMT Drugs

Characteristic	MA-PD (2019Q1 - 2021Q4)			PDP (2019Q1 - 2022Q4)		
	Low formulary coverage	High formulary coverage	P-value	Low formulary coverage	High formulary coverage	P-value
Part D plans, n	1,972	1,297	--	850	874	--
Unique beneficiaries, n	19,251	19,824	--	36,036	30,421	--
Beneficiary-quarter pairs, n	89,027	102,302	--	191,908	175,925	--
**Beneficiary characteristics**						
Age at end of follow-up quarter, mean (SD) [median]	57.6 (10.2) [58]	58.8 (10.4) [60]	<0.001	57.1 (11.9) [58]	60.1 (12.0) [62]	<0.001
Age group at end of follow-up quarter, n (%)			<0.001			<0.001
<=49	18,981 (21.3)	19,428 (19.0)		52,753 (27.5)	36,583 (20.8)	
50-54	13,085 (14.7)	13,490 (13.2)		24,644 (12.8)	18,365 (10.4)	
55-59	16,827 (18.9)	17,685 (17.3)		27,358 (14.3)	22,168 (12.6)	
60-64	16,616 (18.7)	18,801 (18.4)		24,496 (12.8)	22,678 (12.9)	
65-69	13,099 (14.7)	17,480 (17.1)		32,999 (17.2)	32,236 (18.3)	
70-74	7,440 (8.4)	10,877 (10.6)		20,891 (10.9)	28,963 (16.5)	
75+	2,979 (3.3)	4,541 (4.4)		8,767 (4.6)	14,932 (8.5)	
Sex, n (%)			0.37			0.03
Female	68,945 (77.4)	78,782 (77.0)		142,962 (74.5)	132,462 (75.3)	
Male	20,082 (22.6)	23,520 (23.0)		48,946 (25.5)	43,463 (24.7)	
Race/ethnicity, n (%)			<0.001			<0.001
African American/Black	18,975 (21.3)	16,307 (15.9)		27,283 (14.2)	20,038 (11.4)	
Hispanic/Latinx	3,045 (3.4)	3,008 (2.9)		5,753 (3.0)	3,808 (2.2)	
White	64,541 (72.5)	80,270 (78.5)		151,247 (78.8)	145,295 (82.6)	
Other	2,466 (2.8)	2,717 (2.7)		7,625 (4.0)	6,784 (3.9)	
Region in current quarter, n (%)			<0.001			<0.001
Northeast	14,286 (16.0)	25,237 (24.7)		44,125 (23.0)	43,497 (24.7)	
South	43,521 (48.9)	30,358 (29.7)		59,047 (30.8)	53,254 (30.3)	
West	11,580 (13.0)	24,632 (24.1)		36,404 (19.0)	33,462 (19.0)	
Midwest	19,640 (22.1)	22,075 (21.6)		52,332 (27.3)	45,712 (26.0)	
Original reason for Medicare eligibility, n (%)			<0.001			<0.001
OASI	7,140 (8.0)	12,370 (12.1)		29,954 (15.6)	37,396 (21.3)	
Disability and/or ESRD	81,887 (92.0)	89,932 (87.9)		161,954 (84.4)	138,529 (78.7)	
Any subsidy in current quarter (dual eligible and/or LIS), n (%)	46,878 (52.7)	52,913 (51.7)	0.10	129,060 (67.3)	90,826 (51.6)	<0.001

© 2025 Blaylock B et al. *JAMA Network Open*.

Characteristic	MA-PD (2019Q1 - 2021Q4) Low formulary coverage	MA-PD (2019Q1 - 2021Q4) High formulary coverage	MA-PD P-value	PDP (2019Q1 - 2022Q4) Low formulary coverage	PDP (2019Q1 - 2022Q4) High formulary coverage	PDP P-value
**Clinical characteristics**						
Baseline EDSS-DDI, mean (SD) [median]	2.6 (2.7) [1]	2.6 (2.7) [1]	0.004	2.7 (2.8) [2]	2.7 (2.8) [2]	0.39
Baseline MS DMT use, n (%)						
Oral	38,150 (42.9)	42,814 (41.9)	0.07	68,121 (35.5)	67,146 (38.2)	<0.001
Injection	39,471 (44.3)	47,870 (46.8)	<0.001	85,640 (44.6)	80,306 (45.6)	0.01
IV	14,393 (16.2)	14,995 (14.7)	<0.001	44,968 (23.4)	34,125 (19.4)	<0.001
Baseline CCI score, mean (SD) [median]	0.9 (1.4) [0]	1.0 (1.5) [0]	<0.001	0.9 (1.4) [0]	0.9 (1.4) [0]	0.46
Baseline CCI score, n (%)			<0.001			0.003
0	51,237 (57.6)	54,329 (53.1)		113,205 (59.0)	101,804 (57.9)	
1	16,924 (19.0)	20,066 (19.6)		34,552 (18.0)	33,177 (18.9)	
2	10,699 (12.0)	13,362 (13.1)		22,225 (11.6)	20,883 (11.9)	
3+	10,167 (11.4)	14,545 (14.2)		21,926 (11.4)	20,061 (11.4)	
**Formulary coverage**						
Moving average percent of MS DMTs on formulary during baseline, mean (SD) [median]	50.0 (15.1) [45.0]	86.3 (10.3) [87.5]	<0.001	32.7 (5.8) [32.2]	53.3 (18.8) [50.0]	<0.001
Moving average percent of MS drug classes on formulary during baseline, mean (SD) [median]	66.7 (10.3) [65.0]	92.8 (8.4) [100.0]	<0.001	56.1 (6.0) [57.1]	72.9 (13.4) [70.0]	<0.001
Moving average percent of on formulary MS DMTs under PA/ST during baseline, mean (SD) [median]	85.8 (22.6) [100.0]	27.0 (37.8) [6.3]	<0.001	78.4 (29.7) [100.0]	56.1 (44.8) [75.0]	<0.001
**MS relapse**						
Binary/count, n (%)						
Any	6,900 (7.8)	7,043 (6.9)	<0.001	20,428 (10.6)	16,689 (9.5)	<0.001
1	6,657 (7.5)	6,772 (6.6)		19,539 (10.2)	15,984 (9.1)	
2+	243 (0.3)	271 (0.3)		889 (0.5)	705 (0.4)	
Inpatient treatment	558 (0.6)	485 (0.5)	<0.001	1,767 (0.9)	1,453 (0.8)	0.006
Outpatient treatment	6,454 (7.2)	6,655 (6.5)	<0.001	19,068 (9.9)	15,499 (8.8)	<0.001
Count, mean (SD)						
Any	0.1 (0.3)	0.1 (0.3)	<0.001	0.1 (0.3)	0.1 (0.3)	<0.001
Inpatient treatment	0.0 (0.1)	0.0 (0.1)	<0.001	0.0 (0.1)	0.0 (0.1)	0.003
Outpatient treatment	0.1 (0.3)	0.1 (0.3)	<0.001	0.1 (0.3)	0.1 (0.3)	<0.001
**Other utilization**						
All-cause						
Inpatient stays						
Any, n (%)	4,287 (4.8)	4,835 (4.7)	0.46	11,677 (6.1)	10,484 (6.0)	0.20

© 2025 Blaylock B et al. *JAMA Network Open*.

Characteristic	MA-PD (2019Q1 - 2021Q4) Low formulary coverage	MA-PD (2019Q1 - 2021Q4) High formulary coverage	MA-PD P-value	PDP (2019Q1 - 2022Q4) Low formulary coverage	PDP (2019Q1 - 2022Q4) High formulary coverage	PDP P-value
Count, mean (SD)	0.1 (0.3)	0.1 (0.3)	0.78	0.1 (0.4)	0.1 (0.4)	0.38
ED visits						
Any, n (%)	9,302 (10.4)	10,494 (10.3)	0.30	21,870 (11.4)	18,419 (10.5)	<0.001
Count, mean (SD)	0.1 (0.5)	0.1 (0.5)	0.62	0.2 (0.6)	0.1 (0.5)	<0.001
Office visits						
Any, n (%)	72,108 (81.0)	80,964 (79.1)	<0.001	147,218 (76.7)	139,315 (79.2)	<0.001
Count, mean (SD)	2.4 (2.4)	2.3 (2.4)	0.002	2.2 (2.3)	2.4 (2.4)	<0.001
MS-related						
Inpatient stays						
Any, n (%)	4,067 (4.6)	4,603 (4.5)	0.56	11,231 (5.9)	10,113 (5.7)	0.28
Count, mean (SD)	0.1 (0.3)	0.1 (0.3)	0.73	0.1 (0.3)	0.1 (0.3)	0.47
ED visits						
Any, n (%)	5,903 (6.6)	6,637 (6.5)	0.33	13,213 (6.9)	11,155 (6.3)	<0.001
Count, mean (SD)	0.1 (0.4)	0.1 (0.4)	0.45	0.1 (0.4)	0.1 (0.3)	<0.001
Office visits						
Any, n (%)	58,595 (65.8)	67,374 (65.9)	0.89	112,454 (58.6)	106,026 (60.3)	<0.001
Count, mean (SD)	1.2 (1.4)	1.3 (1.5)	<0.001	1.0 (1.2)	1.0 (1.2)	<0.001

Abbreviations: --, not reported; CCI, Charlson comorbidity index; EDSS DDI, expanded disability status scale disability-derived impairments; ESRD, end stage renal disease; LIS, low income subsidy; MA-PD, Medicare Advantage Prescription Drug plan; MS, multiple sclerosis; PA, prior authorization; OASI, old-age & survivors insurance; PDP, Prescription Drug Plan; SD, standard deviation; ST, step therapy.

Notes: Low/high formulary coverage defined by median 4-quarter moving average proportion of drugs on formulary of qualified MS population by quarter and plan type. Statistically significant difference in characteristics between low/high formulary coverage groups calculated using univariable logistic regressions with clustering for repeated observations by beneficiary. Observations for 2020Q2 were dropped from the analysis. Race/ethnicity was reported in the Medicare data as Non-Hispanic White, Black (Or African-American), Asian/Pacific Islander, Hispanic, American Indian/Alaska Native, Other, or Unknown. The categories for Asian/Pacific Islander, American Indian/Alaska Native, Other, or Unknown were combined due to small sample size.

© 2025 Blaylock B et al. *JAMA Network Open*.

eTable 3. Patient Characteristics by Plan Type and MS DMT Formulary Coverage: Binary Coverage Based on MS DMT Classes

Characteristic	MA-PD (2019Q1 - 2021Q4)			PDP (2019Q1 - 2022Q4)		
	Low formulary coverage	High formulary coverage	P-value	Low formulary coverage	High formulary coverage	P-value
Part D plans, n	1,869	1,357	--	844	806	--
Unique beneficiaries, n	17,538	20,445	--	31,659	34,823	--
Beneficiary-quarter pairs, n	83,277	108,052	--	174,499	193,334	--
**Beneficiary characteristics**						
Age at end of follow-up quarter, mean (SD) [median]	57.5 (10.2) [58]	58.8 (10.4) [60]	<0.001	57.6 (11.9) [59]	59.3 (12.2) [61]	<0.001
Age group at end of follow-up quarter, n (%)			<0.001			<0.001
<=49	17,752 (21.3)	20,657 (19.1)		45,625 (26.1)	43,711 (22.6)	
50-54	12,200 (14.6)	14,375 (13.3)		21,699 (12.4)	21,310 (11.0)	
55-59	15,752 (18.9)	18,760 (17.4)		24,679 (14.1)	24,847 (12.9)	
60-64	15,751 (18.9)	19,666 (18.2)		22,499 (12.9)	24,675 (12.8)	
65-69	12,267 (14.7)	18,312 (16.9)		31,574 (18.1)	33,661 (17.4)	
70-74	6,859 (8.2)	11,458 (10.6)		19,855 (11.4)	29,999 (15.5)	
75+	2,696 (3.2)	4,824 (4.5)		8,568 (4.9)	15,131 (7.8)	
Sex, n (%)			0.64			0.33
Female	64,408 (77.3)	83,319 (77.1)		130,323 (74.7)	145,101 (75.1)	
Male	18,869 (22.7)	24,733 (22.9)		44,176 (25.3)	48,233 (24.9)	
Race/ethnicity, n (%)			<0.001			<0.001
African American/Black	17,959 (21.6)	17,323 (16.0)		23,897 (13.7)	23,424 (12.1)	
Hispanic/Latinx	2,830 (3.4)	3,223 (3.0)		4,771 (2.7)	4,790 (2.5)	
White	60,161 (72.2)	84,650 (78.3)		139,115 (79.7)	157,427 (81.4)	
Other	2,327 (2.8)	2,856 (2.6)		6,716 (3.8)	7,693 (4.0)	
Region in current quarter, n (%)			<0.001			<0.001
Northeast	13,047 (15.7)	26,476 (24.5)		38,991 (22.3)	48,631 (25.2)	
South	42,665 (51.2)	31,214 (28.9)		55,119 (31.6)	57,182 (29.6)	
West	10,436 (12.5)	25,776 (23.9)		31,715 (18.2)	38,151 (19.7)	
Midwest	17,129 (20.6)	24,586 (22.8)		48,674 (27.9)	49,370 (25.5)	
Original reason for Medicare eligibility, n (%)			<0.001			<0.001
OASI	6,583 (7.9)	12,927 (12.0)		29,182 (16.7)	38,168 (19.7)	
Disability and/or ESRD	76,694 (92.1)	95,125 (88.0)		145,317 (83.3)	155,166 (80.3)	
Any subsidy in current quarter (dual eligible and/or LIS), n (%)	43,169 (51.8)	56,622 (52.4)	0.33	111,130 (63.7)	108,756 (56.3)	<0.001

© 2025 Blaylock B et al. *JAMA Network Open*.

Characteristic	MA-PD (2019Q1 - 2021Q4) Low formulary coverage	MA-PD (2019Q1 - 2021Q4) High formulary coverage	MA-PD P-value	PDP (2019Q1 - 2022Q4) Low formulary coverage	PDP (2019Q1 - 2022Q4) High formulary coverage	PDP P-value
**Clinical characteristics**						
Baseline EDSS-DDI, mean (SD) [median]	2.6 (2.7) [1]	2.6 (2.7) [1]	0.008	2.7 (2.8) [2]	2.7 (2.8) [2]	0.97
Baseline MS DMT use, n (%)						
Oral	35,454 (42.6)	45,510 (42.1)	0.42	60,442 (34.6)	74,825 (38.7)	<0.001
Injection	37,263 (44.7)	50,078 (46.3)	0.006	80,517 (46.1)	85,429 (44.2)	<0.001
IV	13,373 (16.1)	16,015 (14.8)	0.002	39,766 (22.8)	39,327 (20.3)	<0.001
Baseline CCI score, mean (SD) [median]	0.9 (1.4) [0]	1.0 (1.5) [0]	<0.001	0.9 (1.4) [0]	0.9 (1.4) [0]	0.17
Baseline CCI score, n (%)			<0.001			<0.001
0	48,295 (58.0)	57,271 (53.0)		103,250 (59.2)	111,759 (57.8)	
1	15,743 (18.9)	21,247 (19.7)		31,318 (17.9)	36,411 (18.8)	
2	9,891 (11.9)	14,170 (13.1)		20,067 (11.5)	23,041 (11.9)	
3+	9,348 (11.2)	15,364 (14.2)		19,864 (11.4)	22,123 (11.4)	
**Formulary coverage**						
Moving average percent of MS DMTs on formulary during baseline, mean (SD) [median]	49.2 (15.4) [45.0]	85.0 (11.4) [87.5]	<0.001	34.5 (8.7) [33.8]	49.8 (19.4) [45.0]	<0.001
Moving average percent of MS drug classes on formulary during baseline, mean (SD) [median]	65.3 (8.9) [65.0]	92.5 (8.4) [100.0]	<0.001	55.2 (5.0) [55.0]	72.2 (13.1) [70.0]	<0.001
Moving average percent of on formulary MS DMTs under PA/ST during baseline, mean (SD) [median]	85.1 (23.0) [100.0]	30.6 (39.9) [7.5]	<0.001	77.9 (28.0) [100.0]	58.6 (45.3) [83.3]	<0.001
**MS relapse**						
Binary/count, n (%)						
Any	6,387 (7.7)	7,556 (7.0)	<0.001	18,149 (10.4)	18,968 (9.8)	<0.001
1	6,160 (7.4)	7,269 (6.7)		17,361 (9.9)	18,162 (9.4)	
2+	227 (0.3)	287 (0.3)		788 (0.5)	806 (0.4)	
Inpatient treatment	501 (0.6)	542 (0.5)	0.01	1,580 (0.9)	1,640 (0.8)	0.11
Outpatient treatment	5,980 (7.2)	7,129 (6.6)	<0.001	16,927 (9.7)	17,640 (9.1)	<0.001
Count, mean (SD)						
Any	0.1 (0.3)	0.1 (0.3)	<0.001	0.1 (0.3)	0.1 (0.3)	<0.001
Inpatient treatment	0.0 (0.1)	0.0 (0.1)	0.02	0.0 (0.1)	0.0 (0.1)	0.10
Outpatient treatment	0.1 (0.3)	0.1 (0.3)	<0.001	0.1 (0.3)	0.1 (0.3)	<0.001
**Other utilization**						
All-cause						
Inpatient stays						
Any, n (%)	3,983 (4.8)	5,139 (4.8)	0.83	10,477 (6.0)	11,684 (6.0)	0.69

© 2025 Blaylock B et al. *JAMA Network Open*.

Characteristic	MA-PD (2019Q1 - 2021Q4)			PDP (2019Q1 - 2022Q4)		
	Low formulary coverage	High formulary coverage	P-value	Low formulary coverage	High formulary coverage	P-value
Count, mean (SD)	0.1 (0.3)	0.1 (0.3)	0.56	0.1 (0.4)	0.1 (0.4)	0.83
ED visits						
Any, n (%)	8,610 (10.3)	11,186 (10.4)	0.94	19,313 (11.1)	20,976 (10.8)	0.12
Count, mean (SD)	0.1 (0.5)	0.1 (0.5)	0.70	0.2 (0.6)	0.1 (0.5)	0.01
Office visits						
Any, n (%)	67,232 (80.7)	85,840 (79.4)	<0.001	134,589 (77.1)	151,944 (78.6)	<0.001
Count, mean (SD)	2.3 (2.3)	2.4 (2.5)	0.11	2.2 (2.3)	2.4 (2.4)	<0.001
MS-related						
Inpatient stays						
Any, n (%)	3,779 (4.5)	4,891 (4.5)	0.92	10,075 (5.8)	11,269 (5.8)	0.57
Count, mean (SD)	0.1 (0.3)	0.1 (0.3)	0.52	0.1 (0.3)	0.1 (0.3)	0.77
ED visits						
Any, n (%)	5,469 (6.6)	7,071 (6.5)	0.88	11,832 (6.8)	12,536 (6.5)	0.005
Count, mean (SD)	0.1 (0.4)	0.1 (0.4)	0.79	0.1 (0.4)	0.1 (0.4)	0.003
Office visits						
Any, n (%)	54,251 (65.1)	71,718 (66.4)	<0.001	103,308 (59.2)	115,172 (59.6)	0.12
Count, mean (SD)	1.2 (1.3)	1.3 (1.5)	<0.001	1.0 (1.2)	1.0 (1.2)	0.77

Abbreviations: --, not reported; CCI, Charlson comorbidity index; EDSS DDI, expanded disability status scale disability-derived impairments; ESRD, end stage renal disease; LIS, low income subsidy; MA-PD, Medicare Advantage Prescription Drug plan; MS, multiple sclerosis; PA, prior authorization; OASI, old-age & survivors insurance; PDP, Prescription Drug Plan; SD, standard deviation; ST, step therapy.

Notes: Low/high formulary coverage defined by median 4-quarter moving average proportion of drug classes on formulary of qualified MS population by quarter and plan type. Statistically significant difference in characteristics between low/high formulary coverage groups calculated using univariable logistic regressions with clustering for repeated observations by beneficiary. Observations for 2020Q2 were dropped from the analysis. Race/ethnicity was reported in the Medicare data as Non-Hispanic White, Black (Or African-American), Asian/Pacific Islander, Hispanic, American Indian/Alaska Native, Other, or Unknown. The categories for Asian/Pacific Islander, American Indian/Alaska Native, Other, or Unknown were combined due to small sample size.

© 2025 Blaylock B et al. *JAMA Network Open*.

eTable 4. Patient Characteristics by Plan Type and MS DMT Formulary Coverage: Tertiary Coverage Based on MS DMT Drugs

Characteristic	MA-PD (2019Q1 - 2021Q4)				PDP (2019Q1 - 2022Q4)			
	Low formulary coverage	Moderate formulary coverage	High formulary coverage	P-value	Low formulary coverage	Moderate formulary coverage	High formulary coverage	P-value
Part D plans, n	1,106	1,350	798	--	725	711	821	--
Unique beneficiaries, n	13,064	17,468	8,579	--	27,807	28,253	25,939	--
Beneficiary-quarter pairs, n	66,761	82,736	41,832	--	143,504	107,045	117,284	--
**Beneficiary characteristics**								
Age at end of follow-up quarter, mean (SD) [median]	57.4 (10.2) [58]	58.0 (10.4) [59]	60.1 (10.2) [61]	<0.001	57.1 (11.8) [58]	57.0 (12.2) [58]	61.6 (11.6) [64]	<0.001
Age group at end of follow-up quarter, n (%)				<0.001				<0.001
<=49	14,550 (21.8)	17,253 (20.9)	6,606 (15.8)		39,194 (27.3)	29,935 (28.0)	20,207 (17.2)	
50-54	9,882 (14.8)	11,802 (14.3)	4,891 (11.7)		18,413 (12.8)	13,622 (12.7)	10,974 (9.4)	
55-59	12,603 (18.9)	14,914 (18.0)	6,995 (16.7)		20,610 (14.4)	15,047 (14.1)	13,869 (11.8)	
60-64	12,689 (19.0)	15,205 (18.4)	7,523 (18.0)		18,324 (12.8)	13,536 (12.6)	15,314 (13.1)	
65-69	9,752 (14.6)	12,576 (15.2)	8,251 (19.7)		25,242 (17.6)	16,914 (15.8)	23,079 (19.7)	
70-74	5,184 (7.8)	7,815 (9.4)	5,318 (12.7)		15,410 (10.7)	12,452 (11.6)	21,992 (18.8)	
75+	2,101 (3.1)	3,171 (3.8)	2,248 (5.4)		6,311 (4.4)	5,539 (5.2)	11,849 (10.1)	
Sex, n (%)				0.56				0.13
Female	51,443 (77.1)	64,345 (77.8)	31,939 (76.4)		107,056 (74.6)	80,056 (74.8)	88,312 (75.3)	
Male	15,318 (22.9)	18,391 (22.2)	9,893 (23.6)		36,448 (25.4)	26,989 (25.2)	28,972 (24.7)	
Race/ethnicity, n (%)				<0.001				<0.001
African American/Black	14,753 (22.1)	15,591 (18.8)	4,938 (11.8)		20,354 (14.2)	15,036 (14.0)	11,931 (10.2)	
Hispanic/Latinx	2,327 (3.5)	2,910 (3.5)	816 (2.0)		4,140 (2.9)	3,396 (3.2)	2,025 (1.7)	
White	47,779 (71.6)	62,076 (75.0)	34,956 (83.6)		113,417 (79.0)	84,204 (78.7)	98,921 (84.3)	
Other	1,902 (2.8)	2,159 (2.6)	1,122 (2.7)		5,593 (3.9)	4,409 (4.1)	4,407 (3.8)	
Region in current quarter, n (%)				<0.001				0.02
Northeast	9,244 (13.8)	15,715 (19.0)	14,564 (34.8)		32,770 (22.8)	26,584 (24.8)	28,268 (24.1)	
South	36,069 (54.0)	31,311 (37.8)	6,499 (15.5)		45,049 (31.4)	30,670 (28.7)	36,582 (31.2)	
West	8,005 (12.0)	16,860 (20.4)	11,347 (27.1)		26,453 (18.4)	22,787 (21.3)	20,626 (17.6)	
Midwest	13,443 (20.1)	18,850 (22.8)	9,422 (22.5)		39,232 (27.3)	27,004 (25.2)	31,808 (27.1)	

© 2025 Blaylock B et al. *JAMA Network Open*.

Characteristic	MA-PD (2019Q1 - 2021Q4)				PDP (2019Q1 - 2022Q4)			
	Low formulary coverage	Moderate formulary coverage	High formulary coverage	P-value	Low formulary coverage	Moderate formulary coverage	High formulary coverage	P-value
Original reason for Medicare eligibility, n (%)				<0.001				<0.001
OASI	4,903 (7.3)	8,271 (10.0)	6,336 (15.1)		22,659 (15.8)	16,517 (15.4)	28,174 (24.0)	
Disability and/or ESRD	61,858 (92.7)	74,465 (90.0)	35,496 (84.9)		120,845 (84.2)	90,528 (84.6)	89,110 (76.0)	
Any subsidy in current quarter (dual eligible and/or LIS), n (%)	34,534 (51.7)	48,285 (58.4)	16,972 (40.6)	<0.001	96,027 (66.9)	71,357 (66.7)	52,502 (44.8)	<0.001
**Clinical characteristics**								
Baseline EDSS-DDI, mean (SD) [median]	2.6 (2.7) [1]	2.7 (2.8) [2]	2.5 (2.7) [1]	0.17	2.7 (2.8) [2]	2.7 (2.8) [2]	2.7 (2.8) [2]	0.68
Baseline MS DMT use, n (%)								
Oral	28,712 (43.0)	36,597 (44.2)	15,655 (37.4)	<0.001	50,108 (34.9)	42,163 (39.4)	42,996 (36.7)	<0.001
Injection	29,390 (44.0)	36,440 (44.0)	21,511 (51.4)	<0.001	65,532 (45.7)	43,838 (41.0)	56,576 (48.2)	<0.001
IV	10,961 (16.4)	12,614 (15.2)	5,813 (13.9)	<0.001	32,992 (23.0)	24,852 (23.2)	21,249 (18.1)	<0.001
Baseline CCI score, mean (SD) [median]	0.9 (1.4) [0]	1.1 (1.6) [0]	0.8 (1.3) [0]	0.17	0.9 (1.4) [0]	0.9 (1.4) [0]	0.9 (1.4) [0]	0.01
Baseline CCI score, n (%)				0.47				<0.001
0	38,841 (58.2)	41,750 (50.5)	24,975 (59.7)		84,957 (59.2)	63,161 (59.0)	66,891 (57.0)	
1	12,625 (18.9)	16,513 (20.0)	7,852 (18.8)		25,636 (17.9)	19,724 (18.4)	22,369 (19.1)	
2	7,933 (11.9)	11,374 (13.7)	4,754 (11.4)		16,526 (11.5)	12,328 (11.5)	14,254 (12.2)	
3+	7,362 (11.0)	13,099 (15.8)	4,251 (10.2)		16,385 (11.4)	11,832 (11.1)	13,770 (11.7)	
**Formulary coverage**								
Moving average percent of MS DMTs on formulary during baseline, mean (SD) [median]	43.8 (10.9) [40.6]	77.1 (9.8) [77.2]	95.0 (6.8) [100.0]	<0.001	32.5 (5.2) [33.8]	37.3 (8.6) [35.7]	59.7 (19.4) [57.8]	<0.001
Moving average percent of MS drug classes on formulary during baseline, mean (SD) [median]	63.2 (7.4) [61.9]	87.0 (11.0) [85.0]	96.1 (6.5) [100.0]	<0.001	54.9 (5.2) [55.0]	64.4 (9.2) [61.9]	75.1 (14.6) [71.4]	<0.001
Moving average percent of on formulary MS DMTs under PA/ST during baseline, mean (SD) [median]	87.8 (18.9) [100.0]	25.9 (39.7) [0.0]	56.5 (37.9) [59.4]	<0.001	76.3 (29.0) [100.0]	68.1 (43.1) [100.0]	56.9 (43.7) [75.0]	<0.001
**MS relapse**								
Binary/count, n (%)								

© 2025 Blaylock B et al. *JAMA Network Open*.

Characteristic	MA-PD (2019Q1 - 2021Q4) Low formulary coverage	MA-PD Moderate formulary coverage	MA-PD High formulary coverage	MA-PD P-value	PDP (2019Q1 - 2022Q4) Low formulary coverage	PDP Moderate formulary coverage	PDP High formulary coverage	PDP P-value
Any	5,207 (7.8)	6,055 (7.3)	2,681 (6.4)	<0.001	15,060 (10.5)	11,486 (10.7)	10,571 (9.0)	<0.001
1	5,018 (7.5)	5,819 (7.0)	2,592 (6.2)		14,385 (10.0)	11,019 (10.3)	10,119 (8.6)	
2+	189 (0.3)	236 (0.3)	89 (0.2)		675 (0.5)	467 (0.4)	452 (0.4)	
Inpatient treatment	431 (0.6)	465 (0.6)	147 (0.4)	<0.001	1,340 (0.9)	969 (0.9)	911 (0.8)	<0.001
Outpatient treatment	4,859 (7.3)	5,688 (6.9)	2,562 (6.1)	<0.001	14,037 (9.8)	10,707 (10.0)	9,823 (8.4)	<0.001
Count, mean (SD)								
Any	0.1 (0.3)	0.1 (0.3)	0.1 (0.3)	<0.001	0.1 (0.3)	0.1 (0.3)	0.1 (0.3)	<0.001
Inpatient treatment	0.0 (0.1)	0.0 (0.1)	0.0 (0.1)	<0.001	0.0 (0.1)	0.0 (0.1)	0.0 (0.1)	<0.001
Outpatient treatment	0.1 (0.3)	0.1 (0.3)	0.1 (0.3)	<0.001	0.1 (0.3)	0.1 (0.3)	0.1 (0.3)	<0.001
**Other utilization**								
All-cause								
Inpatient stays								
Any, n (%)	3,150 (4.7)	4,104 (5.0)	1,868 (4.5)	0.37	8,725 (6.1)	6,463 (6.0)	6,973 (5.9)	0.27
Count, mean (SD)	0.1 (0.3)	0.1 (0.4)	0.1 (0.3)	0.58	0.1 (0.4)	0.1 (0.3)	0.1 (0.4)	0.40
ED visits								
Any, n (%)	6,848 (10.3)	9,161 (11.1)	3,787 (9.1)	0.001	16,201 (11.3)	12,393 (11.6)	11,695 (10.0)	<0.001
Count, mean (SD)	0.1 (0.5)	0.2 (0.5)	0.1 (0.4)	0.007	0.2 (0.6)	0.2 (0.6)	0.1 (0.5)	<0.001
Office visits								
Any, n (%)	53,986 (80.9)	66,443 (80.3)	32,643 (78.0)	<0.001	110,187 (76.8)	82,673 (77.2)	93,673 (79.9)	<0.001
Count, mean (SD)	2.3 (2.3)	2.5 (2.5)	2.2 (2.3)	<0.001	2.2 (2.3)	2.3 (2.4)	2.4 (2.5)	<0.001
MS-related								
Inpatient stays								
Any, n (%)	2,979 (4.5)	3,921 (4.7)	1,770 (4.2)	0.46	8,390 (5.8)	6,231 (5.8)	6,723 (5.7)	0.34
Count, mean (SD)	0.1 (0.3)	0.1 (0.3)	0.1 (0.3)	0.61	0.1 (0.3)	0.1 (0.3)	0.1 (0.3)	0.49
ED visits								
Any, n (%)	4,347 (6.5)	5,798 (7.0)	2,395 (5.7)	0.007	9,965 (6.9)	7,366 (6.9)	7,037 (6.0)	<0.001
Count, mean (SD)	0.1 (0.4)	0.1 (0.4)	0.1 (0.3)	0.006	0.1 (0.4)	0.1 (0.4)	0.1 (0.3)	<0.001
Office visits								
Any, n (%)	43,444 (65.1)	56,376 (68.1)	26,149 (62.5)	0.001	84,595 (58.9)	62,907 (58.8)	70,978 (60.5)	<0.001
Count, mean (SD)	1.2 (1.3)	1.4 (1.6)	1.1 (1.3)	0.09	1.0 (1.2)	1.0 (1.2)	1.0 (1.2)	0.02

Abbreviations: --, not reported; CCI, Charlson comorbidity index; EDSS DDI, expanded disability status scale disability-derived impairments; ESRD, end stage renal disease; LIS, low income subsidy; MA-PD, Medicare Advantage Prescription Drug plan; MS, multiple sclerosis; PA, prior authorization; OASI, old-age & survivors insurance; PDP, Prescription Drug Plan; SD, standard deviation; ST, step therapy.

Notes: Low/moderate/high formulary coverage defined by median 4-quarter moving average proportion of drugs on formulary of qualified MS population by quarter and plan type. Statistically significant difference in characteristics between low/moderate/high formulary coverage groups calculated using univariable ordinal multinomial regressions with clustering for repeated observations by beneficiary. Observations for 2020Q2 were dropped from the analysis. Race/ethnicity was reported in the Medicare data as Non-Hispanic White, Black (Or African-American), Asian/Pacific Islander, Hispanic, American Indian/Alaska Native, Other, or Unknown. The categories for Asian/Pacific Islander, American Indian/Alaska Native, Other, or Unknown were combined due to small sample size.

© 2025 Blaylock B et al. *JAMA Network Open*.

eTable 5. Patient Characteristics by Plan Type and MS DMT Formulary Coverage: Tertiary Coverage Based on MS DMT Classes

Characteristic	MA-PD (2019Q1 - 2021Q4)				PDP (2019Q1 - 2022Q4)			
	Low formulary coverage	Moderate formulary coverage	High formulary coverage	P-value	Low formulary coverage	Moderate formulary coverage	High formulary coverage	P-value
Part D plans, n	1,284	1,399	1,112	--	761	849	589	--
Unique beneficiaries, n	14,801	16,180	14,835	--	24,597	35,308	23,331	--
Beneficiary-quarter pairs, n	73,534	57,194	60,601	--	122,357	115,825	129,651	--
**Beneficiary characteristics**								
Age at end of follow-up quarter, mean (SD) [median]	57.5 (10.2) [58]	58.1 (10.4) [59]	59.2 (10.3) [60]	<0.001	58.0 (11.8) [59]	57.2 (12.1) [58]	60.1 (12.1) [62]	<0.001
Age group at end of follow-up quarter, n (%)				<0.001				<0.001
<=49	15,797 (21.5)	11,556 (20.2)	11,056 (18.2)		30,422 (24.9)	31,832 (27.5)	27,082 (20.9)	
50-54	10,792 (14.7)	8,148 (14.2)	7,635 (12.6)		14,833 (12.1)	14,657 (12.7)	13,519 (10.4)	
55-59	13,861 (18.8)	10,239 (17.9)	10,412 (17.2)		17,163 (14.0)	16,227 (14.0)	16,136 (12.4)	
60-64	14,013 (19.1)	10,357 (18.1)	11,047 (18.2)		15,865 (13.0)	14,785 (12.8)	16,524 (12.7)	
65-69	10,772 (14.6)	9,067 (15.9)	10,740 (17.7)		23,167 (18.9)	18,854 (16.3)	23,214 (17.9)	
70-74	5,899 (8.0)	5,584 (9.8)	6,834 (11.3)		14,615 (11.9)	13,305 (11.5)	21,934 (16.9)	
75+	2,400 (3.3)	2,243 (3.9)	2,877 (4.7)		6,292 (5.1)	6,165 (5.3)	11,242 (8.7)	
Sex, n (%)				0.47				0.14
Female	56,805 (77.2)	44,394 (77.6)	46,528 (76.8)		91,385 (74.7)	86,325 (74.5)	97,714 (75.4)	
Male	16,729 (22.8)	12,800 (22.4)	14,073 (23.2)		30,972 (25.3)	29,500 (25.5)	31,937 (24.6)	
Race/ethnicity, n (%)				<0.001				<0.001
African American/Black	16,074 (21.9)	10,267 (18.0)	8,941 (14.8)		16,427 (13.4)	15,866 (13.7)	15,028 (11.6)	
Hispanic/Latinx	2,565 (3.5)	1,886 (3.3)	1,602 (2.6)		3,311 (2.7)	3,503 (3.0)	2,747 (2.1)	
White	52,810 (71.8)	43,555 (76.2)	48,446 (79.9)		97,777 (79.9)	91,901 (79.3)	106,864 (82.4)	
Other	2,085 (2.8)	1,486 (2.6)	1,612 (2.7)		4,842 (4.0)	4,555 (3.9)	5,012 (3.9)	
Region in current quarter, n (%)				<0.001				<0.001
Northeast	10,939 (14.9)	12,231 (21.4)	16,353 (27.0)		27,812 (22.7)	25,948 (22.4)	33,862 (26.1)	
South	39,334 (53.5)	19,456 (34.0)	15,089 (24.9)		38,808 (31.7)	34,886 (30.1)	38,607 (29.8)	
West	9,069 (12.3)	10,462 (18.3)	16,681 (27.5)		22,332 (18.3)	22,769 (19.7)	24,765 (19.1)	
Midwest	14,192 (19.3)	15,045 (26.3)	12,478 (20.6)		33,405 (27.3)	32,222 (27.8)	32,417 (25.0)	

© 2025 Blaylock B et al. *JAMA Network Open*.

Characteristic	MA-PD (2019Q1 - 2021Q4)				PDP (2019Q1 - 2022Q4)			
	Low formulary coverage	Moderate formulary coverage	High formulary coverage	P-value	Low formulary coverage	Moderate formulary coverage	High formulary coverage	P-value
Original reason for Medicare eligibility, n (%)				<0.001				<0.001
OASI	5,618 (7.6)	5,860 (10.2)	8,032 (13.3)		21,661 (17.7)	17,924 (15.5)	27,765 (21.4)	
Disability and/or ESRD	67,916 (92.4)	51,334 (89.8)	52,569 (86.7)		100,696 (82.3)	97,901 (84.5)	101,886 (78.6)	
Any subsidy in current quarter (dual eligible and/or LIS), n (%)	38,025 (51.7)	33,659 (58.9)	28,107 (46.4)	<0.001	76,459 (62.5)	76,056 (65.7)	67,371 (52.0)	<0.001
**Clinical characteristics**								
Baseline EDSS-DDI, mean (SD) [median]	2.6 (2.7) [1]	2.6 (2.7) [2]	2.6 (2.7) [1]	0.47	2.7 (2.8) [2]	2.8 (2.8) [2]	2.7 (2.8) [2]	0.43
Baseline MS DMT use, n (%)								
Oral	31,475 (42.8)	25,325 (44.3)	24,164 (39.9)	<0.001	42,048 (34.4)	41,981 (36.2)	51,238 (39.5)	<0.001
Injection	32,661 (44.4)	24,356 (42.6)	30,324 (50.0)	<0.001	55,699 (45.5)	51,408 (44.4)	58,839 (45.4)	0.83
IV	11,905 (16.2)	9,454 (16.5)	8,029 (13.2)	<0.001	28,581 (23.4)	26,772 (23.1)	23,740 (18.3)	<0.001
Baseline CCI score, mean (SD) [median]	0.9 (1.4) [0]	1.1 (1.6) [0]	1.0 (1.5) [0]	<0.001	0.9 (1.4) [0]	0.9 (1.4) [0]	0.9 (1.4) [0]	0.74
Baseline CCI score, n (%)				<0.001				<0.001
0	42,666 (58.0)	29,309 (51.2)	33,591 (55.4)		72,439 (59.2)	67,637 (58.4)	74,933 (57.8)	
1	13,936 (19.0)	11,403 (19.9)	11,651 (19.2)		21,701 (17.7)	21,366 (18.4)	24,662 (19.0)	
2	8,747 (11.9)	7,785 (13.6)	7,529 (12.4)		14,165 (11.6)	13,605 (11.7)	15,338 (11.8)	
3+	8,185 (11.1)	8,697 (15.2)	7,830 (12.9)		14,052 (11.5)	13,217 (11.4)	14,718 (11.4)	
**Formulary coverage**								
Moving average percent of MS DMTs on formulary during baseline, mean (SD) [median]	46.0 (12.8) [41.6]	75.3 (8.7) [75.3]	92.3 (7.3) [88.0]	<0.001	32.7 (7.8) [31.2]	37.1 (9.8) [37.5]	56.7 (19.2) [50.0]	<0.001
Moving average percent of MS drug classes on formulary during baseline, mean (SD) [median]	63.2 (7.0) [64.3]	83.5 (5.2) [83.3]	99.1 (3.1) [100.0]	<0.001	53.8 (5.1) [52.5]	60.0 (5.0) [60.0]	77.6 (12.3) [79.2]	<0.001
Moving average percent of on formulary MS DMTs under PA/ST during baseline, mean (SD) [median]	87.8 (18.7) [100.0]	35.2 (42.8) [6.3]	31.8 (38.8) [11.3]	<0.001	86.1 (23.2) [100.0]	69.7 (35.0) [83.3]	48.7 (45.8) [50.0]	<0.001
**MS relapse**								
Binary/count, n (%)								

© 2025 Blaylock B et al. *JAMA Network Open*.

Characteristic	MA-PD (2019Q1 - 2021Q4)				PDP (2019Q1 - 2022Q4)			
	Low formulary coverage	Moderate formulary coverage	High formulary coverage	P-value	Low formulary coverage	Moderate formulary coverage	High formulary coverage	P-value
Any	5,607 (7.6)	4,342 (7.6)	3,994 (6.6)	<0.001	12,625 (10.3)	12,485 (10.8)	12,007 (9.3)	<0.001
1	5,406 (7.3)	4,173 (7.3)	3,850 (6.4)		12,100 (9.9)	11,937 (10.3)	11,486 (8.9)	
2+	201 (0.3)	169 (0.3)	144 (0.2)		525 (0.4)	548 (0.5)	521 (0.4)	
Inpatient treatment	457 (0.6)	262 (0.5)	324 (0.5)	0.04	1,058 (0.9)	1,106 (1.0)	1,056 (0.8)	0.21
Outpatient treatment	5,237 (7.1)	4,139 (7.2)	3,733 (6.2)	<0.001	11,811 (9.7)	11,610 (10.0)	11,146 (8.6)	<0.001
Count, mean (SD)								
Any	0.1 (0.3)	0.1 (0.3)	0.1 (0.3)	<0.001	0.1 (0.3)	0.1 (0.3)	0.1 (0.3)	<0.001
Inpatient treatment	0.0 (0.1)	0.0 (0.1)	0.0 (0.1)	0.07	0.0 (0.1)	0.0 (0.1)	0.0 (0.1)	0.17
Outpatient treatment	0.1 (0.3)	0.1 (0.3)	0.1 (0.3)	<0.001	0.1 (0.3)	0.1 (0.3)	0.1 (0.3)	<0.001
**Other utilization**								
All-cause								
Inpatient stays								
Any, n (%)	3,494 (4.8)	2,671 (4.7)	2,957 (4.9)	0.43	7,217 (5.9)	7,249 (6.3)	7,695 (5.9)	0.82
Count, mean (SD)	0.1 (0.3)	0.1 (0.3)	0.1 (0.4)	0.11	0.1 (0.4)	0.1 (0.4)	0.1 (0.4)	0.87
ED visits								
Any, n (%)	7,524 (10.2)	6,079 (10.6)	6,193 (10.2)	0.92	13,054 (10.7)	13,659 (11.8)	13,576 (10.5)	0.17
Count, mean (SD)	0.1 (0.5)	0.1 (0.5)	0.1 (0.5)	0.81	0.1 (0.6)	0.2 (0.6)	0.1 (0.5)	0.009
Office visits								
Any, n (%)	59,405 (80.8)	45,841 (80.2)	47,826 (78.9)	<0.001	93,681 (76.6)	90,017 (77.7)	102,835 (79.3)	<0.001
Count, mean (SD)	2.3 (2.3)	2.5 (2.6)	2.3 (2.3)	0.04	2.2 (2.3)	2.3 (2.3)	2.4 (2.5)	<0.001
MS-related								
Inpatient stays								
Any, n (%)	3,305 (4.5)	2,546 (4.5)	2,819 (4.7)	0.30	6,923 (5.7)	7,009 (6.1)	7,412 (5.7)	0.68
Count, mean (SD)	0.1 (0.3)	0.1 (0.3)	0.1 (0.4)	0.08	0.1 (0.3)	0.1 (0.3)	0.1 (0.3)	0.83
ED visits								
Any, n (%)	4,769 (6.5)	3,820 (6.7)	3,951 (6.5)	0.77	7,910 (6.5)	8,080 (7.0)	8,378 (6.5)	0.90
Count, mean (SD)	0.1 (0.4)	0.1 (0.4)	0.1 (0.4)	0.88	0.1 (0.4)	0.1 (0.4)	0.1 (0.4)	0.28
Office visits								
Any, n (%)	47,935 (65.2)	38,251 (66.9)	39,783 (65.6)	0.10	71,397 (58.4)	68,847 (59.4)	78,236 (60.3)	<0.001
Count, mean (SD)	1.2 (1.3)	1.3 (1.6)	1.2 (1.4)	<0.001	1.0 (1.2)	1.0 (1.2)	1.0 (1.2)	<0.001

Abbreviations: --, not reported; CCI, Charlson comorbidity index; EDSS DDI, expanded disability status scale disability-derived impairments; ESRD, end stage renal disease; LIS, low income subsidy; MA-PD, Medicare Advantage Prescription Drug plan; MS, multiple sclerosis; PA, prior authorization; OASI, old-age & survivors insurance; PDP, Prescription Drug Plan; SD, standard deviation; ST, step therapy.

Notes: Low/moderate/high formulary coverage defined by median 4-quarter moving average proportion of classes on formulary of qualified MS population by quarter and plan type. Statistically significant difference in characteristics between low/moderate/high formulary coverage groups calculated using univariable ordinal multinomial regressions with clustering for repeated observations by beneficiary. Observations for 2020Q2 were dropped from the analysis. Race/ethnicity was reported in the Medicare data as Non-Hispanic White, Black (Or African-American), Asian/Pacific Islander, Hispanic, American Indian/Alaska Native, Other, or Unknown. The categories for Asian/Pacific Islander, American Indian/Alaska Native, Other, or Unknown were combined due to small sample size.

eTable 6. Drug Costs and Coverage for Part D MS DMTs, 2022 (Sorted by Route of Administration and Date Entered Market)

Drug compound	Drug class	Date entered market	Total cost per script, 2022, mean (SD)	OOP cost per script, 2022, mean (SD)	Plans without compound on formulary (weighted by enrollment), %	
					MA-PD	PDP
**Oral MS DMTs**						
Fingolimod hydrochloride	S1PRs	23-Sep-10	$10,080 ($9,381)	$73 ($721)	1.7%	3.4%
Teriflunomide	Pyrimidine synthesis inhibitor	21-Sep-12	$10,212 ($13,748)	$180 ($1,380)	48.0%	91.1%
Dimethyl fumarate	Fumarates	29-Mar-13	$4,818 ($14,467)	$97 ($738)	4.7%	21.1%
Siponimod	S1PRs	28-Mar-19	$9,950 ($10,690)	$60 ($640)	51.5%	96.6%
Cladribine	Purine analog	2-Apr-19	$70,702 ($22,720)	$429 ($1,651)	83.5%	98.9%
Diroximel fumarate	Fumarates	1-Nov-19	$9,167 ($8,559)	$161 ($1,016)	74.9%	74.6%
Ozanimod hydrochloride	S1PRs	26-May-20	$9,532 ($9,355)	$118 ($747)	87.2%	99.9%
Monomethyl fumarate	Fumarates	4-Aug-20	$8,247 ($8,374)	$151 ($760)	89.5%	99.8%
Ponesimod	S1PRs	18-Mar-21	$10,527 ($11,178)	$182 ($861)	92.1%	99.9%
**Injectable DMTs**						
Interferon beta-1B	Interferons	9-Sep-93	$9,115 ($7,504)	$161 ($1,003)	2.9%	0.0%
Interferon beta-1A/albumin human	Interferons	23-May-96	$10,130 ($9,335)	$280 ($1,279)	51.0%	90.1%
Glatiramer acetate	Amino acid copolymer	11-Mar-97	$5,126 ($9,594)	$213 ($1,097)	0.0%	0.0%
Interferon beta-1A	Interferons	28-Jul-03	$8,548 ($8,929)	$202 ($1,234)	31.9%	88.1%
Peginterferon beta-1A	Interferons	6-Oct-14	$9,234 ($10,628)	$164 ($1,014)	70.7%	98.3%
Ofatumumab	Monoclonal antibodies	24-Aug-20	$9,694 ($9,115)	$55 ($727)	74.7%	99.8%

Abbreviations: FFS, fee-for-service; MA-PD, Medicare Advantage Prescription Drug plan; OOP, out-of-pocket; PDP, stand-alone Prescription Drug Plan; PUF, public use file; S1PR, sphingosine-1-phosphate receptor modulator.
Notes: Coverage information from PUF formularies representative of January 2022. Plan enrollment from PUF represents enrollment at beginning of calendar year.

© 2025 Blaylock B et al. *JAMA Network Open*.

eFigure 2. Coverage of MS DMT Drugs (A) and Classes (B) on Part D Formularies, All Medicare, 2022

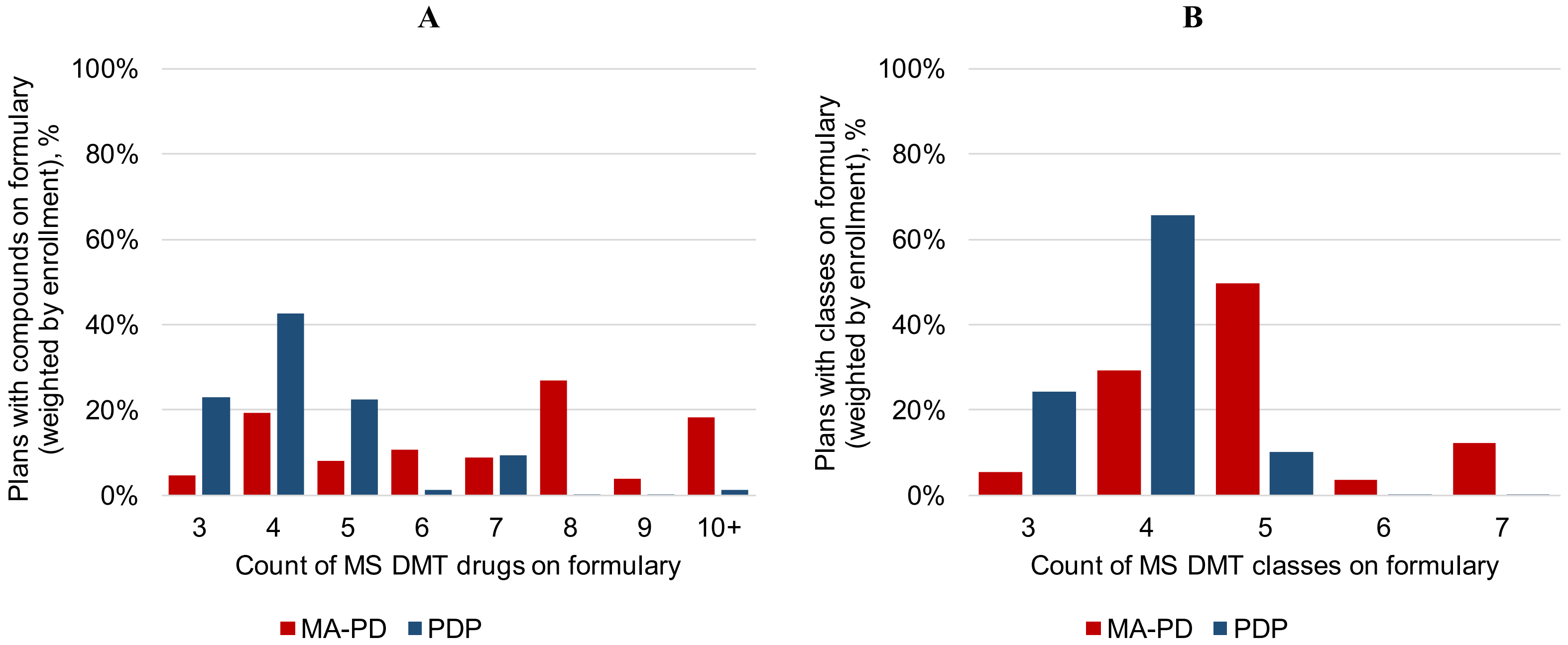

Abbreviations: DMT, disease modifying therapy; MA-PD, Medicare Advantage Prescription Drug plan; MS, multiple sclerosis; PDP, stand-alone Prescription Drug Plan; PUF, public use file.
Notes: The maximum number of compounds in 2022 was 15. Coverage information from PUF formularies representative of January 2022. Plan enrollment from PUF represents enrollment at beginning of calendar year.

© 2025 Blaylock B et al. *JAMA Network Open*.

eFigure 3. Coverage of MS DMT Drugs (A) and Classes (B) on Part D Formularies, Unweighted, 2022

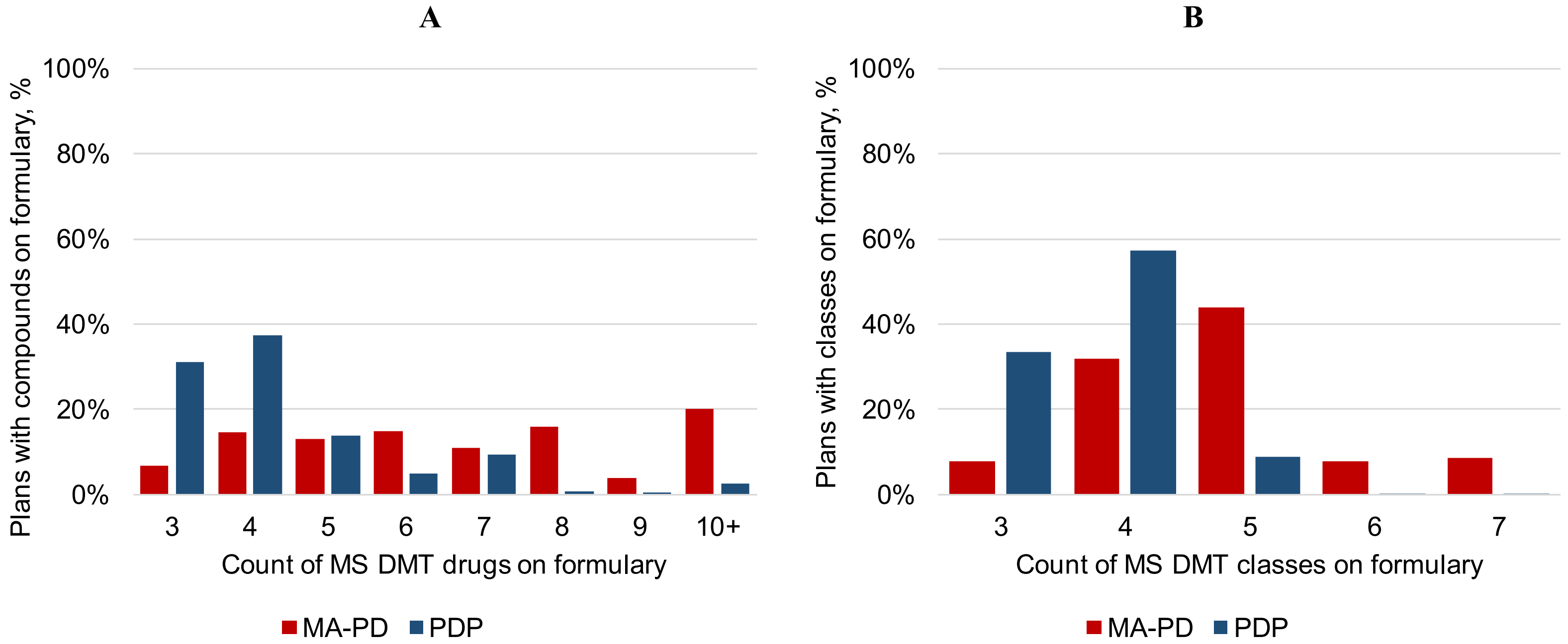

Abbreviations: DMT, disease modifying therapy; MA-PD, Medicare Advantage Prescription Drug plan; MS, multiple sclerosis; PDP, stand-alone Prescription Drug Plan; PUF, public use file.

Notes: The maximum number of compounds in 2022 was 15. Coverage information from PUF formularies representative of January 2022.

© 2025 Blaylock B et al. *JAMA Network Open*.

eFigure 4. Coverage of MS DMT Drugs (A) and Classes (B) on Part D Formularies, RRMS Sample, 2019

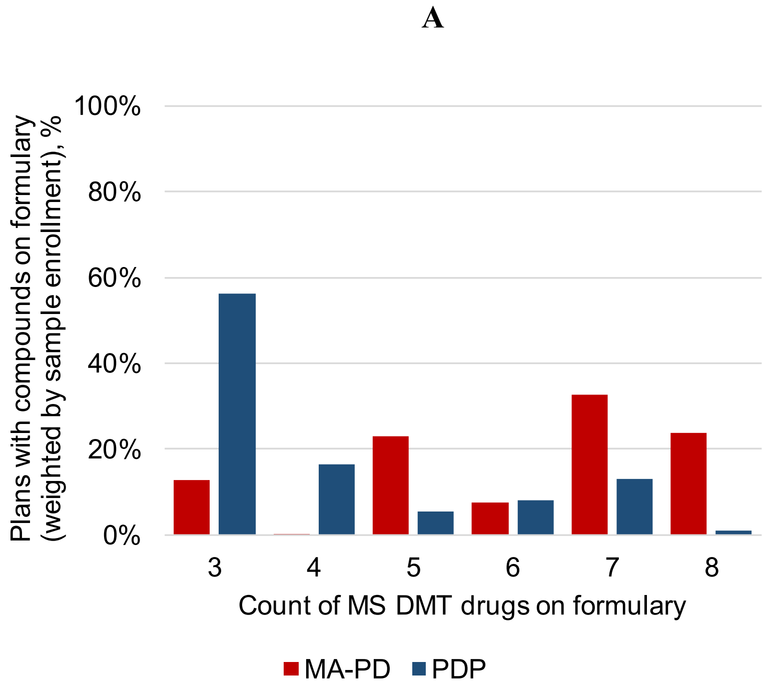

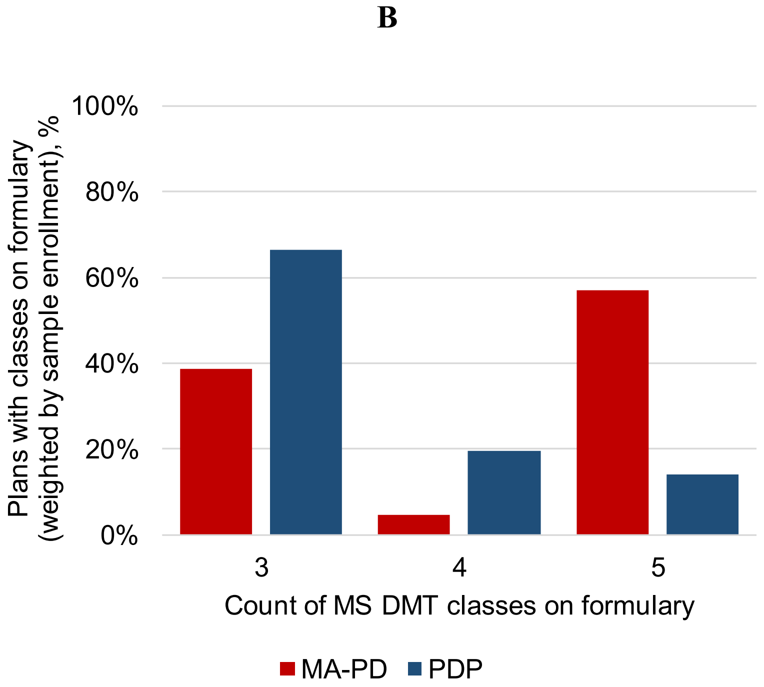

Abbreviations: DMT, disease modifying therapy; MA-PD, Medicare Advantage Prescription Drug plan; MS, multiple sclerosis; PDP, stand-alone Prescription Drug Plan; PUF, public use file; RRMS, relapsing-remitting multiple sclerosis.
Notes: Coverage information from PUF formularies representative of December 2018.

© 2025 Blaylock B et al. *JAMA Network Open*.

eTable 7. Multivariable Logistic Regressions of any MS Relapse (Overall, Inpatient Treatment, Outpatient Treatment) During Follow-Up Quarter: Binary Coverage Based on MS DMT Drugs

Characteristic	MA-PD (2019Q1 - 2021Q4)			PDP (2019Q1 - 2022Q4)		
	Any MS relapse OR (95% CI)	Any MS relapse with IP treatment OR (95% CI)	Any MS relapse with OP treatment OR (95% CI)	Any MS relapse with IP treatment OR (95% CI)	Any MS relapse with IP treatment OR (95% CI)	Any MS relapse with OP treatment OR (95% CI)
**Formulary coverage**						
High formulary coverage [ref=low]	0.88 (0.83, 0.94)	0.82 (0.68, 1.00)	0.89 (0.83, 0.95)	0.93 (0.90, 0.96)	1.02 (0.94, 1.11)	0.92 (0.89, 0.95)
Moving average percent of on formulary MS DMTs under PA/ST during baseline	0.97 (0.90, 1.05)	1.08 (0.87, 1.35)	0.97 (0.89, 1.05)	0.98 (0.93, 1.04)	1.05 (0.92, 1.20)	0.98 (0.92, 1.03)
**Beneficiary characteristics**						
Age group at end of current quarter						
<=49 [ref]						
50-54	0.70 (0.65, 0.75)	0.77 (0.62, 0.96)	0.69 (0.64, 0.75)	0.74 (0.70, 0.78)	0.64 (0.56, 0.73)	0.75 (0.71, 0.79)
55-59	0.62 (0.58, 0.67)	0.50 (0.41, 0.62)	0.63 (0.58, 0.67)	0.65 (0.61, 0.68)	0.61 (0.53, 0.69)	0.65 (0.61, 0.69)
60-64	0.49 (0.45, 0.53)	0.46 (0.36, 0.57)	0.49 (0.45, 0.53)	0.59 (0.56, 0.63)	0.65 (0.56, 0.75)	0.58 (0.55, 0.62)
65-69	0.39 (0.36, 0.43)	0.37 (0.28, 0.48)	0.39 (0.36, 0.43)	0.55 (0.52, 0.58)	0.44 (0.38, 0.51)	0.56 (0.52, 0.59)
70-74	0.30 (0.27, 0.34)	0.36 (0.26, 0.51)	0.30 (0.26, 0.34)	0.45 (0.42, 0.49)	0.50 (0.42, 0.59)	0.45 (0.42, 0.48)
75+	0.26 (0.22, 0.31)	0.39 (0.25, 0.62)	0.25 (0.21, 0.30)	0.39 (0.35, 0.43)	0.58 (0.47, 0.71)	0.38 (0.34, 0.42)
Female	0.92 (0.87, 0.98)	0.96 (0.81, 1.14)	0.92 (0.87, 0.98)	0.85 (0.82, 0.89)	0.97 (0.88, 1.07)	0.84 (0.81, 0.88)
Race/ethnicity						
African American/Black	1.07 (1.01, 1.14)	1.26 (1.06, 1.51)	1.06 (0.99, 1.13)	1.00 (0.95, 1.05)	1.74 (1.57, 1.93)	0.93 (0.88, 0.99)
Hispanic/Latinx	0.99 (0.87, 1.14)	1.64 (1.19, 2.26)	0.95 (0.82, 1.09)	0.98 (0.88, 1.09)	1.53 (1.23, 1.91)	0.93 (0.83, 1.04)
White [ref]						

© 2025 Blaylock B et al. *JAMA Network Open*.

Other	1.06 (0.90, 1.24)	1.75 (1.19, 2.58)	1.01 (0.85, 1.20)	0.98 (0.89, 1.07)	1.20 (0.94, 1.53)	0.96 (0.87, 1.06)
Region in current quarter						
Northeast [ref]						
South	0.83 (0.77, 0.88)	1.01 (0.82, 1.25)	0.81 (0.76, 0.87)	0.77 (0.74, 0.81)	1.09 (0.97, 1.22)	0.75 (0.71, 0.79)
West	0.77 (0.70, 0.83)	0.75 (0.58, 0.98)	0.77 (0.71, 0.84)	0.92 (0.87, 0.98)	0.84 (0.73, 0.97)	0.93 (0.88, 0.99)
Midwest	1.12 (1.04, 1.20)	1.03 (0.82, 1.29)	1.12 (1.04, 1.21)	1.01 (0.96, 1.06)	1.02 (0.90, 1.15)	1.01 (0.96, 1.07)
Original reason for Medicare eligibility disability and/or ESRD [ref=OASI]	1.06 (0.94, 1.20)	1.16 (0.80, 1.70)	1.05 (0.93, 1.19)	0.93 (0.87, 1.00)	1.46 (1.24, 1.73)	0.90 (0.84, 0.97)
**Clinical characteristics**						
Baseline EDSS-DDI	1.09 (1.08, 1.10)	1.15 (1.12, 1.18)	1.08 (1.07, 1.09)	1.08 (1.08, 1.09)	1.15 (1.14, 1.17)	1.08 (1.07, 1.08)
Baseline CCI score						
0 [ref]						
1	1.14 (1.07, 1.20)	1.16 (0.96, 1.40)	1.13 (1.07, 1.20)	1.10 (1.05, 1.14)	1.44 (1.30, 1.60)	1.07 (1.03, 1.12)
2	1.16 (1.08, 1.23)	1.67 (1.37, 2.03)	1.12 (1.05, 1.20)	1.06 (1.01, 1.11)	1.45 (1.29, 1.63)	1.02 (0.97, 1.08)
3+	1.23 (1.14, 1.32)	1.98 (1.62, 2.43)	1.19 (1.10, 1.28)	1.03 (0.98, 1.09)	1.96 (1.75, 2.21)	0.96 (0.91, 1.02)
**Study period**						
2019 Q1 [ref]						
2019 Q2	1.05 (0.97, 1.14)	0.85 (0.66, 1.09)	1.08 (0.99, 1.17)	1.02 (0.97, 1.08)	0.92 (0.79, 1.07)	1.03 (0.97, 1.09)
2019 Q3	1.04 (0.97, 1.12)	1.07 (0.83, 1.36)	1.04 (0.97, 1.12)	1.01 (0.97, 1.06)	1.01 (0.86, 1.17)	1.01 (0.97, 1.06)
2019 Q4	1.01 (0.94, 1.10)	0.84 (0.65, 1.09)	1.03 (0.95, 1.13)	1.01 (0.96, 1.07)	0.87 (0.74, 1.02)	1.03 (0.97, 1.09)
2020 Q1	1.00 (0.92, 1.08)	0.65 (0.49, 0.86)	1.03 (0.95, 1.12)	0.96 (0.91, 1.02)	0.88 (0.74, 1.04)	0.97 (0.91, 1.02)
2020 Q3	1.06 (0.98, 1.15)	0.69 (0.53, 0.91)	1.10 (1.01, 1.19)	1.02 (0.97, 1.08)	0.78 (0.65, 0.93)	1.04 (0.98, 1.11)

© 2025 Blaylock B et al. *JAMA Network Open*.

2020 Q4	0.98 (0.90, 1.07)	0.53 (0.40, 0.72)	1.03 (0.94, 1.12)	0.96 (0.90, 1.02)	0.67 (0.55, 0.81)	0.99 (0.93, 1.06)
2021 Q1	1.09 (1.00, 1.18)	0.70 (0.52, 0.93)	1.13 (1.04, 1.23)	1.01 (0.95, 1.07)	0.67 (0.55, 0.81)	1.04 (0.98, 1.11)
2021 Q2	1.08 (1.00, 1.18)	0.65 (0.49, 0.87)	1.13 (1.03, 1.23)	1.13 (1.07, 1.20)	0.82 (0.68, 0.98)	1.16 (1.09, 1.24)
2021 Q3	1.12 (1.03, 1.21)	0.73 (0.55, 0.96)	1.16 (1.07, 1.26)	1.08 (1.02, 1.15)	0.68 (0.56, 0.82)	1.12 (1.06, 1.20)
2021 Q4	1.12 (1.03, 1.21)	0.62 (0.47, 0.82)	1.17 (1.08, 1.28)	1.13 (1.06, 1.20)	0.65 (0.53, 0.79)	1.17 (1.10, 1.25)
2022 Q1				1.20 (1.13, 1.28)	0.65 (0.53, 0.81)	1.25 (1.18, 1.34)
2022 Q2				1.21 (1.13, 1.29)	0.66 (0.53, 0.81)	1.26 (1.18, 1.35)
2022 Q3				1.19 (1.12, 1.27)	0.71 (0.58, 0.87)	1.24 (1.16, 1.32)
2022 Q4				1.15 (1.08, 1.22)	0.58 (0.47, 0.72)	1.21 (1.13, 1.29)

Abbreviations: CCI, Charlson comorbidity index; EDSS-DDI, expanded disability status scale disability-derived impairments; IP, inpatient; MA-PD, Medicare Advantage Prescription Drug plan; MS, multiple sclerosis; PA, prior authorization; OP, outpatient; OR, odds ratio; PDP, Prescription Drug Plan; ref, reference; SD, standard deviation; ST, step therapy.
Notes: Low/high formulary coverage defined for qualified MS population by quarter and plan type using median 4-quarter moving average proportion of drugs on formulary. Observations for 2020Q2 were dropped from the analysis. Adjusted odds ratios estimated using multivariable logistic regressions controlling for binary or tertiary formulary coverage variable, age group, sex, race/ethnicity, region, baseline EDSS-DDI score, baseline CCI score, original reason for Medicare eligibility, moving average proportion of compounds under PA/ST during baseline, and study period in addition to clustering for repeated observations by beneficiary. Race/ethnicity was reported in the Medicare data as Non-Hispanic White, Black (Or African-American), Asian/Pacific Islander, Hispanic, American Indian/Alaska Native, Other, or Unknown. The categories for Asian/Pacific Islander, American Indian/Alaska Native, Other, or Unknown were combined due to small sample size.

© 2025 Blaylock B et al. *JAMA Network Open*.

eTable 8. Multivariable Logistic Regressions of Any MS Relapse (Overall, Inpatient Treatment, Outpatient Treatment) During Follow-Up Quarter: Binary Coverage Based on MS DMT Classes

Characteristic	MA-PD (2019Q1 - 2021Q4)			PDP (2019Q1 - 2022Q4)		
	Any MS relapse OR (95% CI)	Any MS relapse with IP treatment OR (95% CI)	Any MS relapse with OP treatment OR (95% CI)	Any MS relapse with IP treatment OR (95% CI)	Any MS relapse with IP treatment OR (95% CI)	Any MS relapse with OP treatment OR (95% CI)
**Formulary coverage**						
High formulary coverage [ref=low]	0.92 (0.86, 0.98)	1.01 (0.83, 1.22)	0.91 (0.86, 0.98)	0.94 (0.91, 0.97)	1.07 (0.97, 1.17)	0.93 (0.89, 0.96)
Moving average percent of on formulary MS DMTs under PA/ST during baseline	1.01 (0.93, 1.08)	1.27 (1.03, 1.58)	0.99 (0.92, 1.07)	0.99 (0.93, 1.04)	1.07 (0.94, 1.23)	0.98 (0.92, 1.03)
**Beneficiary characteristics**						
Age group at end of current quarter						
<=49 [ref]						
50-54	0.70 (0.65, 0.75)	0.77 (0.62, 0.96)	0.69 (0.64, 0.75)	0.74 (0.70, 0.78)	0.64 (0.56, 0.73)	0.75 (0.71, 0.79)
55-59	0.62 (0.58, 0.67)	0.50 (0.41, 0.62)	0.63 (0.58, 0.67)	0.65 (0.61, 0.68)	0.61 (0.53, 0.69)	0.65 (0.61, 0.69)
60-64	0.49 (0.45, 0.53)	0.46 (0.36, 0.57)	0.49 (0.45, 0.53)	0.59 (0.56, 0.62)	0.65 (0.56, 0.75)	0.58 (0.55, 0.62)
65-69	0.39 (0.36, 0.43)	0.37 (0.28, 0.48)	0.39 (0.36, 0.43)	0.55 (0.51, 0.58)	0.44 (0.38, 0.51)	0.55 (0.52, 0.59)
70-74	0.30 (0.27, 0.34)	0.36 (0.25, 0.51)	0.30 (0.26, 0.33)	0.45 (0.42, 0.48)	0.50 (0.42, 0.59)	0.45 (0.41, 0.48)
75+	0.26 (0.22, 0.30)	0.39 (0.24, 0.61)	0.25 (0.21, 0.30)	0.39 (0.35, 0.43)	0.58 (0.47, 0.71)	0.38 (0.34, 0.42)
Female	0.92 (0.87, 0.98)	0.96 (0.81, 1.14)	0.92 (0.87, 0.98)	0.85 (0.82, 0.89)	0.97 (0.88, 1.07)	0.84 (0.81, 0.88)
Race/ethnicity						
African American/Black	1.08 (1.01, 1.15)	1.27 (1.07, 1.52)	1.06 (1.00, 1.13)	1.00 (0.95, 1.06)	1.74 (1.57, 1.93)	0.93 (0.88, 0.99)
Hispanic/Latinx	1.00 (0.87, 1.14)	1.65 (1.20, 2.28)	0.95 (0.82, 1.09)	0.98 (0.88, 1.09)	1.53 (1.23, 1.91)	0.93 (0.83, 1.04)
White [ref]						

© 2025 Blaylock B et al. *JAMA Network Open*.

Characteristic	MA-PD (2019Q1 - 2021Q4)			PDP (2019Q1 - 2022Q4)		
	Any MS relapse OR (95% CI)	Any MS relapse with IP treatment OR (95% CI)	Any MS relapse with OP treatment OR (95% CI)	Any MS relapse with IP treatment OR (95% CI)	Any MS relapse with IP treatment OR (95% CI)	Any MS relapse with OP treatment OR (95% CI)
Other	1.06 (0.90, 1.24)	1.76 (1.20, 2.59)	1.01 (0.85, 1.20)	0.98 (0.89, 1.07)	1.20 (0.94, 1.53)	0.96 (0.87, 1.06)
Region in current quarter						
Northeast [ref]						
South	0.83 (0.77, 0.88)	1.03 (0.83, 1.27)	0.81 (0.76, 0.87)	0.77 (0.73, 0.81)	1.09 (0.97, 1.23)	0.75 (0.71, 0.79)
West	0.77 (0.70, 0.83)	0.74 (0.57, 0.97)	0.77 (0.71, 0.84)	0.93 (0.87, 0.98)	0.84 (0.73, 0.97)	0.93 (0.88, 0.99)
Midwest	1.12 (1.04, 1.21)	1.03 (0.82, 1.29)	1.13 (1.04, 1.21)	1.01 (0.96, 1.06)	1.02 (0.90, 1.15)	1.01 (0.96, 1.07)
Original reason for Medicare eligibility disability and/or ESRD [ref=OASI]	1.06 (0.94, 1.20)	1.17 (0.80, 1.70)	1.05 (0.93, 1.19)	0.93 (0.87, 1.00)	1.46 (1.24, 1.73)	0.90 (0.84, 0.97)
**Clinical characteristics**						
Baseline EDSS-DDI	1.09 (1.08, 1.10)	1.15 (1.12, 1.18)	1.08 (1.07, 1.09)	1.08 (1.08, 1.09)	1.15 (1.14, 1.17)	1.08 (1.07, 1.08)
Baseline CCI score						
0 [ref]						
1	1.14 (1.07, 1.20)	1.16 (0.96, 1.40)	1.13 (1.07, 1.21)	1.10 (1.05, 1.14)	1.44 (1.30, 1.60)	1.07 (1.03, 1.12)
2	1.16 (1.08, 1.24)	1.67 (1.37, 2.03)	1.12 (1.05, 1.20)	1.06 (1.01, 1.11)	1.45 (1.29, 1.63)	1.02 (0.97, 1.08)
3+	1.23 (1.14, 1.32)	1.98 (1.62, 2.42)	1.19 (1.10, 1.28)	1.03 (0.98, 1.09)	1.97 (1.75, 2.21)	0.96 (0.91, 1.02)
**Study period**						
2019 Q1 [ref]						
2019 Q2	1.05 (0.97, 1.14)	0.85 (0.66, 1.09)	1.08 (0.99, 1.17)	1.02 (0.97, 1.08)	0.92 (0.79, 1.07)	1.03 (0.97, 1.09)
2019 Q3	1.04 (0.97, 1.12)	1.06 (0.83, 1.36)	1.04 (0.97, 1.12)	1.02 (0.98, 1.07)	0.99 (0.85, 1.16)	1.03 (0.98, 1.08)
2019 Q4	1.01 (0.93, 1.10)	0.83 (0.64, 1.08)	1.03 (0.95, 1.12)	1.02 (0.97, 1.08)	0.86 (0.73, 1.01)	1.04 (0.98, 1.10)

© 2025 Blaylock B et al. *JAMA Network Open*.

Characteristic	MA-PD (2019Q1 - 2021Q4)			PDP (2019Q1 - 2022Q4)		
	Any MS relapse OR (95% CI)	Any MS relapse with IP treatment OR (95% CI)	Any MS relapse with OP treatment OR (95% CI)	Any MS relapse with IP treatment OR (95% CI)	Any MS relapse with IP treatment OR (95% CI)	Any MS relapse with OP treatment OR (95% CI)
2020 Q1	0.99 (0.92, 1.07)	0.64 (0.48, 0.85)	1.03 (0.95, 1.12)	0.97 (0.92, 1.03)	0.86 (0.73, 1.02)	0.98 (0.92, 1.04)
2020 Q3	1.05 (0.97, 1.14)	0.67 (0.51, 0.89)	1.09 (1.01, 1.18)	1.03 (0.97, 1.09)	0.76 (0.64, 0.91)	1.05 (0.99, 1.12)
2020 Q4	0.98 (0.90, 1.07)	0.52 (0.39, 0.70)	1.03 (0.94, 1.12)	0.96 (0.90, 1.02)	0.65 (0.54, 0.79)	1.00 (0.94, 1.07)
2021 Q1	1.09 (1.00, 1.18)	0.68 (0.51, 0.90)	1.13 (1.04, 1.23)	1.01 (0.95, 1.08)	0.65 (0.53, 0.80)	1.05 (0.98, 1.11)
2021 Q2	1.08 (1.00, 1.18)	0.63 (0.47, 0.84)	1.13 (1.03, 1.23)	1.14 (1.07, 1.21)	0.80 (0.67, 0.97)	1.17 (1.10, 1.25)
2021 Q3	1.12 (1.03, 1.21)	0.71 (0.54, 0.93)	1.16 (1.07, 1.26)	1.09 (1.02, 1.15)	0.66 (0.55, 0.81)	1.13 (1.06, 1.20)
2021 Q4	1.12 (1.03, 1.21)	0.60 (0.46, 0.80)	1.17 (1.08, 1.28)	1.13 (1.07, 1.21)	0.64 (0.52, 0.78)	1.18 (1.11, 1.26)
2022 Q1				1.19 (1.12, 1.27)	0.64 (0.52, 0.79)	1.25 (1.17, 1.33)
2022 Q2				1.21 (1.13, 1.29)	0.64 (0.52, 0.79)	1.27 (1.18, 1.35)
2022 Q3				1.24 (1.16, 1.33)	0.68 (0.55, 0.84)	1.30 (1.21, 1.40)
2022 Q4				1.20 (1.12, 1.28)	0.55 (0.44, 0.69)	1.27 (1.18, 1.36)

Abbreviations: CCI, Charlson comorbidity index; EDSS-DDI, expanded disability status scale disability-derived impairments; IP, inpatient; MA-PD, Medicare Advantage Prescription Drug plan; MS, multiple sclerosis; PA, prior authorization; OP, outpatient; OR, odds ratio; PDP, Prescription Drug Plan; ref, reference; SD, standard deviation; ST, step therapy.
Notes: Low/high formulary coverage defined for qualified MS population by quarter and plan type using median 4-quarter moving average proportion of drug classes on formulary. Observations for 2020Q2 were dropped from the analysis. Adjusted odds ratios estimated using multivariable logistic regressions controlling for binary or tertiary formulary coverage variable, age group, sex, race/ethnicity, region, baseline EDSS-DDI score, baseline CCI score, original reason for Medicare eligibility, moving average proportion of compounds under PA/ST during baseline, and study period in addition to clustering for repeated observations by beneficiary. Race/ethnicity was reported in the Medicare data as Non-Hispanic White, Black (Or African-American), Asian/Pacific Islander, Hispanic, American Indian/Alaska Native, Other, or Unknown. The categories for Asian/Pacific Islander, American Indian/Alaska Native, Other, or Unknown were combined due to small sample size.

© 2025 Blaylock B et al. *JAMA Network Open*.

eTable 9. Multivariable Logistic Regressions of Any MS relapse (Overall, Inpatient Treatment, Outpatient Treatment) During Follow-Up Quarter: Tertiary Coverage Based on MS DMT Drugs

Characteristic	MA-PD (2019Q1 - 2021Q4)			PDP (2019Q1 - 2022Q4)		
	Any MS relapse OR (95% CI)	Any MS relapse with IP treatment OR (95% CI)	Any MS relapse with OP treatment OR (95% CI)	Any MS relapse with IP treatment OR (95% CI)	Any MS relapse with IP treatment OR (95% CI)	Any MS relapse with OP treatment OR (95% CI)
**Formulary coverage**						
Level of coverage						
Low [ref]						
Moderate	0.93 (0.87, 1.00)	0.98 (0.80, 1.20)	0.93 (0.87, 1.00)	0.98 (0.94, 1.02)	1.05 (0.95, 1.17)	0.97 (0.93, 1.01)
High	0.88 (0.82, 0.95)	0.67 (0.53, 0.85)	0.90 (0.83, 0.97)	0.92 (0.88, 0.95)	1.02 (0.92, 1.13)	0.90 (0.87, 0.94)
Moving average percent of on formulary MS DMTs under PA/ST during baseline	1.03 (0.95, 1.11)	1.29 (1.04, 1.60)	1.02 (0.94, 1.10)	0.99 (0.94, 1.05)	1.05 (0.92, 1.20)	0.98 (0.93, 1.04)
**Beneficiary characteristics**						
Age group at end of current quarter						
<=49 [ref]						
50-54	0.70 (0.65, 0.75)	0.77 (0.62, 0.96)	0.69 (0.64, 0.75)	0.74 (0.70, 0.78)	0.64 (0.56, 0.73)	0.75 (0.71, 0.79)
55-59	0.62 (0.58, 0.67)	0.51 (0.41, 0.63)	0.63 (0.58, 0.67)	0.65 (0.61, 0.68)	0.61 (0.53, 0.69)	0.65 (0.61, 0.69)
60-64	0.49 (0.45, 0.53)	0.46 (0.36, 0.58)	0.49 (0.45, 0.53)	0.59 (0.56, 0.63)	0.65 (0.56, 0.75)	0.59 (0.55, 0.62)
65-69	0.39 (0.36, 0.43)	0.38 (0.29, 0.49)	0.40 (0.36, 0.43)	0.55 (0.52, 0.58)	0.44 (0.38, 0.51)	0.56 (0.52, 0.59)
70-74	0.30 (0.27, 0.34)	0.37 (0.26, 0.52)	0.30 (0.26, 0.34)	0.46 (0.42, 0.49)	0.50 (0.42, 0.59)	0.45 (0.42, 0.49)
75+	0.26 (0.22, 0.31)	0.40 (0.25, 0.63)	0.25 (0.21, 0.30)	0.39 (0.36, 0.44)	0.58 (0.47, 0.72)	0.38 (0.34, 0.42)
Female	0.92 (0.87, 0.98)	0.96 (0.81, 1.13)	0.92 (0.87, 0.98)	0.85 (0.82, 0.89)	0.97 (0.88, 1.07)	0.84 (0.81, 0.88)
Race/ethnicity						

© 2025 Blaylock B et al. *JAMA Network Open*.

Characteristic	MA-PD (2019Q1 - 2021Q4)			PDP (2019Q1 - 2022Q4)		
	Any MS relapse OR (95% CI)	Any MS relapse with IP treatment OR (95% CI)	Any MS relapse with OP treatment OR (95% CI)	Any MS relapse with IP treatment OR (95% CI)	Any MS relapse with IP treatment OR (95% CI)	Any MS relapse with OP treatment OR (95% CI)
African American/Black	1.07 (1.01, 1.14)	1.25 (1.05, 1.49)	1.06 (0.99, 1.13)	1.00 (0.95, 1.05)	1.74 (1.56, 1.93)	0.93 (0.88, 0.98)
Hispanic/Latinx	0.99 (0.87, 1.13)	1.61 (1.16, 2.21)	0.95 (0.82, 1.09)	0.98 (0.88, 1.09)	1.53 (1.23, 1.90)	0.92 (0.83, 1.04)
White [ref]						
Other	1.06 (0.90, 1.24)	1.74 (1.18, 2.56)	1.01 (0.85, 1.20)	0.97 (0.89, 1.07)	1.20 (0.94, 1.53)	0.96 (0.87, 1.06)
Region in current quarter						
Northeast [ref]						
South	0.81 (0.76, 0.87)	0.94 (0.76, 1.17)	0.81 (0.75, 0.86)	0.77 (0.74, 0.82)	1.09 (0.97, 1.22)	0.75 (0.71, 0.79)
West	0.76 (0.70, 0.83)	0.74 (0.57, 0.96)	0.77 (0.70, 0.84)	0.92 (0.87, 0.98)	0.84 (0.73, 0.97)	0.93 (0.88, 0.98)
Midwest	1.11 (1.03, 1.19)	0.99 (0.79, 1.25)	1.12 (1.04, 1.21)	1.01 (0.96, 1.06)	1.02 (0.90, 1.15)	1.02 (0.97, 1.07)
Original reason for Medicare eligibility disability and/or ESRD [ref=OASI]	1.06 (0.94, 1.20)	1.15 (0.79, 1.69)	1.05 (0.93, 1.19)	0.93 (0.87, 1.00)	1.46 (1.24, 1.73)	0.90 (0.84, 0.97)
**Clinical characteristics**						
Baseline EDSS-DDI	1.09 (1.08, 1.10)	1.15 (1.12, 1.18)	1.08 (1.07, 1.09)	1.08 (1.08, 1.09)	1.15 (1.14, 1.17)	1.08 (1.07, 1.08)
Baseline CCI score						
0 [ref]						
1	1.13 (1.07, 1.20)	1.16 (0.96, 1.40)	1.13 (1.07, 1.20)	1.10 (1.05, 1.14)	1.44 (1.30, 1.60)	1.07 (1.03, 1.12)
2	1.15 (1.08, 1.23)	1.66 (1.36, 2.02)	1.12 (1.05, 1.20)	1.06 (1.01, 1.11)	1.45 (1.29, 1.63)	1.02 (0.97, 1.08)
3+	1.23 (1.14, 1.32)	1.96 (1.60, 2.40)	1.18 (1.10, 1.28)	1.03 (0.98, 1.09)	1.97 (1.75, 2.21)	0.96 (0.91, 1.02)
**Study period**						
2019 Q1 [ref]						

© 2025 Blaylock B et al. *JAMA Network Open*.

Characteristic	MA-PD (2019Q1 - 2021Q4)			PDP (2019Q1 - 2022Q4)		
	Any MS relapse OR (95% CI)	Any MS relapse with IP treatment OR (95% CI)	Any MS relapse with OP treatment OR (95% CI)	Any MS relapse with IP treatment OR (95% CI)	Any MS relapse with IP treatment OR (95% CI)	Any MS relapse with OP treatment OR (95% CI)
2019 Q2	1.05 (0.97, 1.14)	0.85 (0.66, 1.09)	1.08 (0.99, 1.18)	1.02 (0.97, 1.08)	0.92 (0.79, 1.07)	1.03 (0.97, 1.09)
2019 Q3	1.05 (0.97, 1.12)	1.06 (0.83, 1.36)	1.04 (0.97, 1.12)	1.02 (0.97, 1.06)	1.00 (0.86, 1.16)	1.02 (0.97, 1.07)
2019 Q4	1.01 (0.93, 1.10)	0.83 (0.64, 1.08)	1.03 (0.95, 1.12)	1.02 (0.96, 1.07)	0.86 (0.74, 1.01)	1.03 (0.97, 1.09)
2020 Q1	0.99 (0.92, 1.08)	0.64 (0.48, 0.84)	1.03 (0.95, 1.12)	0.97 (0.91, 1.02)	0.87 (0.73, 1.03)	0.97 (0.92, 1.03)
2020 Q3	1.05 (0.97, 1.14)	0.67 (0.51, 0.89)	1.09 (1.00, 1.18)	1.02 (0.96, 1.08)	0.77 (0.64, 0.92)	1.04 (0.98, 1.10)
2020 Q4	0.98 (0.90, 1.06)	0.51 (0.38, 0.69)	1.02 (0.94, 1.11)	0.96 (0.90, 1.02)	0.67 (0.55, 0.80)	1.00 (0.94, 1.06)
2021 Q1	1.08 (0.99, 1.17)	0.66 (0.50, 0.89)	1.12 (1.03, 1.22)	1.01 (0.95, 1.08)	0.67 (0.55, 0.82)	1.04 (0.98, 1.11)
2021 Q2	1.07 (0.99, 1.17)	0.62 (0.46, 0.83)	1.12 (1.03, 1.22)	1.12 (1.06, 1.19)	0.81 (0.67, 0.97)	1.15 (1.08, 1.23)
2021 Q3	1.11 (1.02, 1.20)	0.69 (0.53, 0.92)	1.15 (1.06, 1.25)	1.09 (1.03, 1.16)	0.67 (0.55, 0.82)	1.14 (1.07, 1.21)
2021 Q4	1.11 (1.02, 1.20)	0.59 (0.44, 0.78)	1.16 (1.07, 1.27)	1.12 (1.06, 1.20)	0.64 (0.52, 0.78)	1.17 (1.10, 1.25)
2022 Q1				1.19 (1.12, 1.27)	0.64 (0.52, 0.80)	1.24 (1.17, 1.33)
2022 Q2				1.19 (1.12, 1.27)	0.65 (0.53, 0.80)	1.25 (1.17, 1.33)
2022 Q3				1.21 (1.14, 1.29)	0.69 (0.56, 0.86)	1.27 (1.19, 1.36)
2022 Q4				1.17 (1.09, 1.25)	0.57 (0.45, 0.70)	1.23 (1.15, 1.32)

Abbreviations: CCI, Charlson comorbidity index; EDSS-DDI, expanded disability status scale disability-derived impairments; IP, inpatient; MA-PD, Medicare Advantage Prescription Drug plan; MS, multiple sclerosis; PA, prior authorization; OP, outpatient; OR, odds ratio; PDP, Prescription Drug Plan; ref, reference; SD, standard deviation; ST, step therapy.
Notes: Low/moderate/high formulary coverage defined for qualified MS population by quarter and plan type using median 4-quarter moving average proportion of drugs on formulary. Observations for 2020Q2 were dropped from the analysis. Adjusted odds ratios estimated using multivariable logistic regressions controlling for binary or tertiary formulary coverage variable, age group, sex, race/ethnicity, region, baseline EDSS-DDI score, baseline CCI score, original reason for Medicare eligibility, moving average proportion of compounds under PA/ST during baseline, and study period in addition to clustering for repeated observations by beneficiary. Race/ethnicity was reported in the Medicare data as Non-Hispanic White, Black (Or African-American), Asian/Pacific Islander, Hispanic, American Indian/Alaska Native, Other, or Unknown. The categories for Asian/Pacific Islander, American Indian/Alaska Native, Other, or Unknown were combined due to small sample size.

© 2025 Blaylock B et al. *JAMA Network Open*.

eTable 10. Multivariable Logistic Regressions of Any MS Relapse (Overall, Inpatient Treatment, Outpatient Treatment) During Follow-Up Quarter: Tertiary Coverage Based on MS DMT Classes

	MA-PD (2019Q1 - 2021Q4)			PDP (2019Q1 - 2022Q4)		
Characteristic	Any MS relapse OR (95% CI)	Any MS relapse with IP treatment OR (95% CI)	Any MS relapse with OP treatment OR (95% CI)	Any MS relapse with IP treatment OR (95% CI)	Any MS relapse with IP treatment OR (95% CI)	Any MS relapse with OP treatment OR (95% CI)
Formulary coverage						
Level of coverage						
Low [ref]						
Moderate	0.99 (0.92, 1.06)	0.90 (0.72, 1.11)	1.00 (0.93, 1.07)	1.00 (0.96, 1.04)	1.10 (0.98, 1.23)	0.99 (0.95, 1.03)
High	0.91 (0.85, 0.97)	0.96 (0.77, 1.19)	0.91 (0.84, 0.97)	0.91 (0.87, 0.95)	1.02 (0.91, 1.14)	0.90 (0.86, 0.94)
Moving average percent of on formulary MS DMTs under PA/ST during baseline	1.04 (0.97, 1.12)	1.20 (0.97, 1.48)	1.03 (0.96, 1.11)	0.97 (0.92, 1.02)	1.03 (0.90, 1.19)	0.96 (0.91, 1.02)
Beneficiary characteristics						
Age group at end of current quarter						
<=49 [ref]						
50-54	0.70 (0.65, 0.75)	0.77 (0.62, 0.96)	0.69 (0.64, 0.75)	0.74 (0.70, 0.78)	0.64 (0.56, 0.73)	0.75 (0.71, 0.79)
55-59	0.62 (0.58, 0.67)	0.50 (0.41, 0.62)	0.63 (0.58, 0.67)	0.65 (0.61, 0.68)	0.61 (0.53, 0.69)	0.65 (0.61, 0.69)
60-64	0.49 (0.45, 0.53)	0.45 (0.36, 0.57)	0.49 (0.45, 0.53)	0.59 (0.56, 0.62)	0.65 (0.57, 0.75)	0.58 (0.55, 0.62)
65-69	0.39 (0.36, 0.43)	0.37 (0.28, 0.48)	0.40 (0.36, 0.43)	0.55 (0.52, 0.58)	0.44 (0.38, 0.51)	0.55 (0.52, 0.59)
70-74	0.30 (0.27, 0.34)	0.36 (0.25, 0.51)	0.30 (0.26, 0.34)	0.45 (0.42, 0.49)	0.50 (0.42, 0.59)	0.45 (0.41, 0.48)
75+	0.26 (0.22, 0.31)	0.39 (0.24, 0.61)	0.25 (0.21, 0.30)	0.39 (0.35, 0.43)	0.58 (0.48, 0.72)	0.38 (0.34, 0.42)
Female	0.92 (0.87, 0.98)	0.96 (0.81, 1.14)	0.92 (0.87, 0.98)	0.85 (0.82, 0.89)	0.97 (0.88, 1.07)	0.84 (0.81, 0.88)
Race/ethnicity						

© 2025 Blaylock B et al. *JAMA Network Open*.

Characteristic	MA-PD (2019Q1 - 2021Q4)			PDP (2019Q1 - 2022Q4)		
	Any MS relapse OR (95% CI)	Any MS relapse with IP treatment OR (95% CI)	Any MS relapse with OP treatment OR (95% CI)	Any MS relapse with IP treatment OR (95% CI)	Any MS relapse with IP treatment OR (95% CI)	Any MS relapse with OP treatment OR (95% CI)
African American/Black	1.08 (1.01, 1.15)	1.27 (1.07, 1.51)	1.06 (1.00, 1.13)	1.00 (0.95, 1.06)	1.74 (1.56, 1.93)	0.93 (0.88, 0.99)
Hispanic/Latinx	0.99 (0.87, 1.14)	1.65 (1.20, 2.28)	0.95 (0.82, 1.09)	0.98 (0.88, 1.09)	1.53 (1.23, 1.90)	0.93 (0.83, 1.04)
White [ref]						
Other	1.06 (0.90, 1.24)	1.76 (1.19, 2.58)	1.01 (0.85, 1.20)	0.98 (0.89, 1.07)	1.20 (0.94, 1.53)	0.96 (0.87, 1.06)
Region in current quarter						
Northeast [ref]						
South	0.82 (0.77, 0.88)	1.02 (0.82, 1.26)	0.81 (0.75, 0.87)	0.77 (0.73, 0.81)	1.09 (0.97, 1.22)	0.75 (0.71, 0.79)
West	0.77 (0.70, 0.83)	0.74 (0.57, 0.97)	0.77 (0.71, 0.84)	0.92 (0.87, 0.98)	0.84 (0.73, 0.96)	0.93 (0.88, 0.98)
Midwest	1.11 (1.03, 1.20)	1.03 (0.82, 1.30)	1.12 (1.04, 1.21)	1.01 (0.96, 1.06)	1.01 (0.90, 1.15)	1.01 (0.96, 1.07)
Original reason for Medicare eligibility disability and/or ESRD [ref=OASI]	1.06 (0.94, 1.20)	1.17 (0.80, 1.70)	1.05 (0.93, 1.19)	0.93 (0.87, 1.00)	1.46 (1.24, 1.73)	0.90 (0.84, 0.97)
Clinical characteristics						
Baseline EDSS-DDI	1.09 (1.08, 1.10)	1.15 (1.12, 1.18)	1.08 (1.07, 1.09)	1.08 (1.08, 1.09)	1.15 (1.14, 1.17)	1.08 (1.07, 1.08)
Baseline CCI score						
0 [ref]						
1	1.13 (1.07, 1.20)	1.16 (0.96, 1.40)	1.13 (1.07, 1.20)	1.10 (1.05, 1.14)	1.44 (1.30, 1.60)	1.07 (1.03, 1.12)
2	1.15 (1.08, 1.23)	1.67 (1.37, 2.03)	1.12 (1.05, 1.20)	1.06 (1.01, 1.11)	1.45 (1.29, 1.63)	1.02 (0.97, 1.08)
3+	1.23 (1.14, 1.32)	1.98 (1.62, 2.43)	1.18 (1.10, 1.28)	1.03 (0.98, 1.09)	1.96 (1.75, 2.21)	0.96 (0.91, 1.02)
Study period						
2019 Q1 [ref]						

© 2025 Blaylock B et al. *JAMA Network Open*.

Characteristic	MA-PD (2019Q1 - 2021Q4)			PDP (2019Q1 - 2022Q4)		
	Any MS relapse OR (95% CI)	Any MS relapse with IP treatment OR (95% CI)	Any MS relapse with OP treatment OR (95% CI)	Any MS relapse with IP treatment OR (95% CI)	Any MS relapse with IP treatment OR (95% CI)	Any MS relapse with OP treatment OR (95% CI)
2019 Q2	1.05 (0.97, 1.14)	0.85 (0.66, 1.09)	1.08 (0.99, 1.17)	1.02 (0.97, 1.08)	0.92 (0.79, 1.07)	1.03 (0.97, 1.09)
2019 Q3	1.04 (0.97, 1.12)	1.07 (0.83, 1.37)	1.04 (0.96, 1.12)	1.01 (0.97, 1.07)	1.06 (0.90, 1.24)	1.01 (0.96, 1.06)
2019 Q4	1.01 (0.93, 1.10)	0.84 (0.65, 1.09)	1.03 (0.95, 1.12)	1.02 (0.96, 1.08)	0.92 (0.78, 1.08)	1.02 (0.96, 1.08)
2020 Q1	0.99 (0.91, 1.07)	0.64 (0.48, 0.86)	1.02 (0.94, 1.11)	0.97 (0.91, 1.02)	0.92 (0.77, 1.10)	0.97 (0.91, 1.02)
2020 Q3	1.01 (0.93, 1.09)	0.70 (0.52, 0.94)	1.04 (0.95, 1.13)	1.03 (0.97, 1.10)	0.82 (0.68, 0.99)	1.05 (0.99, 1.11)
2020 Q4	0.94 (0.86, 1.02)	0.54 (0.40, 0.74)	0.98 (0.89, 1.07)	0.96 (0.90, 1.03)	0.71 (0.58, 0.86)	0.99 (0.93, 1.06)
2021 Q1	1.04 (0.95, 1.13)	0.70 (0.52, 0.95)	1.08 (0.99, 1.18)	1.02 (0.96, 1.08)	0.71 (0.57, 0.87)	1.04 (0.98, 1.11)
2021 Q2	1.03 (0.95, 1.13)	0.66 (0.49, 0.88)	1.07 (0.98, 1.17)	1.15 (1.08, 1.23)	0.87 (0.72, 1.06)	1.17 (1.10, 1.25)
2021 Q3	1.07 (0.98, 1.16)	0.74 (0.55, 0.98)	1.11 (1.01, 1.21)	1.10 (1.03, 1.17)	0.71 (0.58, 0.86)	1.13 (1.06, 1.21)
2021 Q4	1.07 (0.98, 1.16)	0.63 (0.47, 0.84)	1.12 (1.02, 1.22)	1.14 (1.07, 1.22)	0.68 (0.56, 0.83)	1.18 (1.11, 1.26)
2022 Q1				1.21 (1.13, 1.29)	0.69 (0.55, 0.85)	1.26 (1.18, 1.34)
2022 Q2				1.17 (1.10, 1.25)	0.67 (0.55, 0.83)	1.22 (1.14, 1.30)
2022 Q3				1.19 (1.12, 1.26)	0.71 (0.58, 0.87)	1.23 (1.16, 1.31)
2022 Q4				1.14 (1.07, 1.22)	0.58 (0.47, 0.72)	1.20 (1.13, 1.28)

Abbreviations: CCI, Charlson comorbidity index; EDSS-DDI, expanded disability status scale disability-derived impairments; IP, inpatient; MA-PD, Medicare Advantage Prescription Drug plan; MS, multiple sclerosis; PA, prior authorization; OP, outpatient; OR, odds ratio; PDP, Prescription Drug Plan; ref, reference; SD, standard deviation; ST, step therapy.
Notes: Low/moderate/high formulary coverage defined for qualified MS population by quarter and plan type using median 4-quarter moving average proportion of classes on formulary. Observations for 2020Q2 were dropped from the analysis. Adjusted odds ratios estimated using multivariable logistic regressions controlling for binary or tertiary formulary coverage variable, age group, sex, race/ethnicity, region, baseline EDSS-DDI score, baseline CCI score, original reason for Medicare eligibility, moving average proportion of compounds under PA/ST during baseline, and study period in addition to clustering for repeated observations by beneficiary. Race/ethnicity was reported in the Medicare data as Non-Hispanic White, Black (Or African-American), Asian/Pacific Islander, Hispanic, American Indian/Alaska Native, Other, or Unknown. The categories for Asian/Pacific Islander, American Indian/Alaska Native, Other, or Unknown were combined due to small sample size.

© 2025 Blaylock B et al. *JAMA Network Open*.

eTable 11. Regressions of Count of MS Relapse (Overall, Inpatient, Outpatient) During Follow-Up Quarter (bold indicates statistical significance)

	MA-PD (2019Q1 - 2021Q4)						PDP (2019Q1 - 2022Q4)					
	MS relapse		MS relapse with IP treatment		MS relapse with OP treatment		MS relapse		MS relapse with IP treatment		MS relapse with OP treatment	
	coef. (95% CI)	P-value	coef. (95% CI)	P-value	coef. (95% CI)	P-value	coef. (95% CI)	P-value	coef. (95% CI)	P-value	coef. (95% CI)	P-value
Formulary coverage defined by drugs												
Binary formulary coverage [reference=Low]												
High	**-0.11 (-0.17, -0.05)**	**<0.001**	-0.16 (-0.36, 0.03)	0.11	**-0.11 (-0.17, -0.05)**	**<0.001**	**-0.06 (-0.09, -0.03)**	**<0.001**	0.00 (-0.08, 0.08)	0.99	**-0.07 (-0.10, -0.04)**	**<0.001**
Tertiary formulary coverage [reference=Low]												
Moderate	-0.07 (-0.13, 0.00)	0.05	-0.02 (-0.22, 0.18)	0.86	-0.07 (-0.13, 0.00)	0.05	-0.02 (-0.06, 0.01)	0.23	0.05 (-0.05, 0.15)	0.30	-0.03 (-0.06, 0.01)	0.12
High	**-0.12 (-0.19, -0.05)**	**<0.001**	**-0.37 (-0.61, -0.13)**	**0.003**	**-0.11 (-0.18, -0.04)**	**0.003**	**-0.08 (-0.12, -0.04)**	**<0.001**	0.01 (-0.10, 0.11)	0.91	**-0.09 (-0.13, -0.05)**	**<0.001**
Formulary coverage defined by drug classes												
Binary formulary coverage [reference=Low]												
High	**-0.08 (-0.14, -0.02)**	**0.007**	0.02 (-0.17, 0.21)	0.83	**-0.09 (-0.15, -0.03)**	**0.006**	**-0.05 (-0.09, -0.02)**	**0.001**	0.06 (-0.03, 0.15)	0.21	**-0.07 (-0.10, -0.03)**	**<0.001**
Tertiary formulary coverage [reference=Low]												
Moderate	-0.01 (-0.07, 0.05)	0.77	-0.10 (-0.31, 0.12)	0.37	0.00 (-0.07, 0.06)	0.93	0.00 (-0.03, 0.03)	0.98	0.10 (-0.01, 0.21)	0.07	-0.01 (-0.05, 0.03)	0.59
High	**-0.09 (-0.16, -0.03)**	**0.005**	-0.03 (-0.25, 0.19)	0.82	**-0.10 (-0.16, -0.03)**	**0.005**	**-0.08 (-0.12, -0.04)**	**<0.001**	0.01 (-0.11, 0.12)	0.92	**-0.09 (-0.13, -0.05)**	**<0.001**

Abbreviations: CCI, Charlson comorbidity index; coef., coefficient; EDSS-DDI, expanded disability status scale disability-derived impairments; IP, inpatient; MA-PD, Medicare Advantage Prescription Drug plan; MS, multiple sclerosis; PA, prior authorization; OP, outpatient; OR, odds ratio; PDP, stand-alone Prescription Drug Plan; SD, standard deviation; ST, step therapy.

Notes: Formulary coverage defined for qualified MS population by quarter and plan type using median 4-quarter moving average proportion of drugs or classes on formulary. Observations for 2020Q2 were dropped from the analysis. Coefficients estimated using multivariable poisson regressions controlling for binary or tertiary formulary coverage, age group, sex, race/ethnicity, region, baseline EDSS-DDI score, baseline CCI score, original reason for Medicare eligibility, proportion of compounds under PA/ST during baseline, and study period in addition to clustering for repeated observations by beneficiary. Race/ethnicity was reported in the Medicare data as Non-Hispanic White, Black (Or African-American), Asian/Pacific Islander, Hispanic, American Indian/Alaska Native, Other, or Unknown. The categories for Asian/Pacific Islander, American Indian/Alaska Native, Other, or Unknown were combined due to small sample size.

© 2025 Blaylock B et al. *JAMA Network Open*.

eTable 12. Regressions of Any or Count of MS Relapse (Overall, Inpatient, Outpatient) During Follow-Up Year in Sensitivity Analysis Sample (bold indicates statistical significance)

	MA-PD (2019 - 2021)			PDP (2019 - 2022)		
	MS relapse	MS relapse with IP treatment	MS relapse with OP treatment	MS relapse	MS relapse with IP treatment	MS relapse with OP treatment
**Logistic (OR, 95% CI)**						
Formulary coverage defined by drugs						
Binary formulary coverage [reference=Low]						
High	**0.89 (0.84, 0.95)**	0.96 (0.81, 1.14)	**0.90 (0.84, 0.96)**	**0.93 (0.89, 0.96)**	1.02 (0.94, 1.11)	**0.92 (0.88, 0.95)**
Tertiary formulary coverage [reference=Low]						
Moderate	**0.87 (0.80, 0.93)**	0.94 (0.78, 1.12)	**0.87 (0.80, 0.93)**	1.00 (0.94, 1.06)	1.07 (0.93, 1.23)	0.98 (0.92, 1.05)
High	**0.84 (0.78, 0.91)**	**0.70 (0.56, 0.87)**	**0.87 (0.81, 0.95)**	**0.91 (0.87, 0.95)**	1.00 (0.92, 1.10)	**0.90 (0.86, 0.94)**
Formulary coverage defined by drug classes						
Binary formulary coverage [reference=Low]						
High	**0.90 (0.84, 0.96)**	0.98 (0.83, 1.16)	**0.91 (0.85, 0.97)**	**0.93 (0.90, 0.97)**	1.00 (0.92, 1.09)	**0.92 (0.89, 0.96)**
Tertiary formulary coverage [reference=Low]						
Moderate	0.93 (0.86, 1.01)	**0.78 (0.62, 0.97)**	0.96 (0.88, 1.04)	0.95 (0.89, 1.01)	1.11 (0.95, 1.31)	0.94 (0.88, 1.01)
High	**0.86 (0.80, 0.92)**	0.96 (0.79, 1.15)	**0.87 (0.81, 0.93)**	**0.91 (0.86, 0.95)**	1.07 (0.94, 1.21)	**0.89 (0.85, 0.94)**
**Negative binomial (coef., 95% CI)**						
Formulary coverage defined by drugs						
Binary formulary coverage [reference=Low]						
High	**-0.11 (-0.16, -0.05)**	-0.07 (-0.26, 0.11)	**-0.11 (-0.17, -0.05)**	**-0.07 (-0.11, -0.04)**	0.01 (-0.07, 0.10)	**-0.08 (-0.12, -0.05)**
Tertiary formulary coverage [reference=Low]						
Moderate	**-0.12 (-0.19, -0.05)**	-0.04 (-0.23, 0.15)	**-0.13 (-0.20, -0.06)**	-0.02 (-0.08, 0.03)	0.08 (-0.07, 0.22)	-0.03 (-0.09, 0.03)
High	**-0.15 (-0.22, -0.08)**	**-0.41 (-0.65, -0.17)**	**-0.14 (-0.21, -0.06)**	**-0.09 (-0.12, -0.05)**	-0.01 (-0.10, 0.09)	**-0.09 (-0.13, -0.06)**

© 2025 Blaylock B et al. *JAMA Network Open*.

Formulary coverage defined by drug classes						
Binary formulary coverage [reference=Low]						
High	**-0.10 (-0.16, -0.05)**	-0.05 (-0.23, 0.13)	**-0.11 (-0.17, -0.05)**	**-0.07 (-0.10, -0.03)**	0.00 (-0.09, 0.09)	**-0.07 (-0.11, -0.04)**
Tertiary formulary coverage [reference=Low]						
Moderate	-0.04 (-0.12, 0.03)	**-0.23 (-0.46, -0.01)**	-0.03 (-0.11, 0.04)	-0.04 (-0.10, 0.01)	0.10 (-0.06, 0.27)	-0.05 (-0.11, 0.01)
High	**-0.15 (-0.21, -0.08)**	-0.08 (-0.28, 0.11)	**-0.15 (-0.22, -0.08)**	**-0.09 (-0.13, -0.04)**	0.07 (-0.07, 0.20)	**-0.10 (-0.15, -0.05)**

Abbreviations: CCI, Charlson comorbidity index; coef., coefficient; EDSS-DDI, expanded disability status scale disability-derived impairments; IP, inpatient; MA-PD, Medicare Advantage Prescription Drug plan; MS, multiple sclerosis; PA, prior authorization; OP, outpatient; OR, odds ratio; PDP, stand-alone Prescription Drug Plan; SD, standard deviation; ST, step therapy.
Notes: Formulary coverage defined for qualified MS population by year and plan type using the proportion of drugs or classes on formulary in January of the baseline period. Adjusted odds ratios (logistic) or coefficients (negative binomial) estimated using multivariable regressions controlling for binary or tertiary formulary coverage, age group, sex, race/ethnicity, region, baseline EDSS-DDI score, baseline CCI score, original reason for Medicare eligibility, proportion of compounds under PA/ST during baseline, and study period in addition to clustering for repeated observations by beneficiary. Race/ethnicity was reported in the Medicare data as Non-Hispanic White, Black (Or African-American), Asian/Pacific Islander, Hispanic, American Indian/Alaska Native, Other, or Unknown. The categories for Asian/Pacific Islander, American Indian/Alaska Native, Other, or Unknown were combined due to small sample size.

© 2025 Blaylock B et al. *JAMA Network Open*.

eTable 13. Regressions of Any All-Cause Utilization (Inpatient, ED, Office Visits) During Follow-Up Quarter (bold indicates statistical significance)

	MA-PD (2019Q1 - 2021Q4)						PDP (2019Q1 - 2022Q4)					
	Any inpatient stays		Any ED visits		Any office visits		Any inpatient stays		Any ED visits		Any office visits	
	aOR (95% CI)	P-value	aOR (95% CI)	P-value	aOR (95% CI)	P-value	aOR (95% CI)	P-value	aOR (95% CI)	P-value	aOR (95% CI)	P-value
Formulary coverage defined by drugs												
Binary formulary coverage [reference=Low]												
High	0.94 (0.88, 1.01)	0.10	0.96 (0.91, 1.01)	0.14	1.04 (1.00, 1.09)	0.05	1.00 (0.97, 1.04)	0.86	0.98 (0.95, 1.01)	0.18	**1.10 (1.07, 1.13)**	**<0.001**
Tertiary formulary coverage [reference=Low]												
Moderate	1.01 (0.94, 1.09)	0.72	**1.07 (1.01, 1.13)**	**0.03**	**1.17 (1.12, 1.24)**	**<0.001**	1.03 (0.99, 1.07)	0.17	1.04 (1.00, 1.07)	0.05	1.01 (0.98, 1.04)	0.46
High	0.97 (0.89, 1.04)	0.38	0.97 (0.92, 1.04)	0.40	1.02 (0.97, 1.07)	0.54	1.00 (0.96, 1.04)	0.86	**0.96 (0.92, 0.99)**	**0.01**	**1.14 (1.10, 1.18)**	**<0.001**
Formulary coverage defined by drug classes												
Binary formulary coverage [reference=Low]												
High	0.96 (0.90, 1.03)	0.24	1.00 (0.95, 1.06)	0.89	**1.11 (1.06, 1.15)**	**<0.001**	1.03 (1.00, 1.07)	0.07	1.03 (0.99, 1.06)	0.11	**1.05 (1.02, 1.08)**	**<0.001**
Tertiary formulary coverage [reference=Low]												
Moderate	0.96 (0.89, 1.03)	0.27	**1.09 (1.03, 1.15)**	**0.005**	**1.20 (1.14, 1.26)**	**<0.001**	1.03 (0.98, 1.08)	0.21	1.04 (1.00, 1.07)	0.05	**0.97 (0.94, 1.00)**	**0.03**
High	1.00 (0.93, 1.08)	0.99	0.99 (0.93, 1.05)	0.72	1.04 (0.99, 1.09)	0.12	1.01 (0.97, 1.06)	0.63	1.01 (0.97, 1.05)	0.55	**1.07 (1.03, 1.10)**	**<0.001**

Abbreviations: aOR, adjusted odds ratio; CCI, Charlson comorbidity index; ED, emergency department; EDSS-DDI, expanded disability status scale disability-derived impairments; IP, inpatient; MA-PD, Medicare Advantage Prescription Drug plan; MS, multiple sclerosis; PA, prior authorization; OP, outpatient; PDP, stand-alone Prescription Drug Plan; SD, standard deviation; ST, step therapy. Notes: Formulary coverage defined for qualified MS population by quarter and plan type using median 4-quarter moving average proportion of drugs or classes on formulary. Observations for 2020Q2 were dropped from the analysis. Adjusted odds ratios estimated using multivariable logistic regressions controlling for binary or tertiary formulary coverage, age group, sex, race/ethnicity, region, baseline EDSS-DDI score, baseline CCI score, original reason for Medicare eligibility, moving average proportion of compounds under PA/ST during baseline, and study period in addition to clustering for repeated observations by beneficiary. Race/ethnicity was reported in the Medicare data as Non-Hispanic White, Black (Or African-American), Asian/Pacific Islander, Hispanic, American Indian/Alaska Native, Other, or Unknown. The categories for Asian/Pacific Islander, American Indian/Alaska Native, Other, or Unknown were combined due to small sample size.

© 2025 Blaylock B et al. *JAMA Network Open*.

eTable 14. Regressions of Count of All-Cause Utilization (Inpatient, ED, Office Visits) During Follow-Up Quarter (bold indicates statistical significance)

	MA-PD (2019Q1 - 2021Q4)						PDP (2019Q1 - 2022Q4)					
	Inpatient stays		ED visits		Office visits		Inpatient stays		ED visits		Office visits	
	coef. (95% CI)	P-value	coef. (95% CI)	P-value	coef. (95% CI)	P-value	coef. (95% CI)	P-value	coef. (95% CI)	P-value	coef. (95% CI)	P-value
Formulary coverage defined by drugs												
Binary formulary coverage [reference=Low]												
High	-0.05 (-0.13, 0.02)	0.15	-0.05 (-0.11, 0.02)	0.15	0.01 (-0.01, 0.03)	0.18	0.01 (-0.03, 0.04)	0.65	**-0.05 (-0.08, -0.02)**	**0.005**	**0.05 (0.04, 0.06)**	**<0.001**
Tertiary formulary coverage [reference=Low]												
Moderate	0.04 (-0.04, 0.11)	0.37	0.05 (-0.02, 0.12)	0.14	**0.11 (0.09, 0.13)**	**<0.001**	0.02 (-0.03, 0.06)	0.41	0.02 (-0.02, 0.07)	0.25	0.01 (-0.01, 0.02)	0.44
High	-0.05 (-0.13, 0.03)	0.22	-0.04 (-0.11, 0.03)	0.27	0.02 (-0.01, 0.04)	0.26	0.00 (-0.04, 0.05)	0.92	**-0.08 (-0.12, -0.04)**	**<0.001**	**0.07 (0.05, 0.08)**	**<0.001**
Formulary coverage defined by drug classes												
Binary formulary coverage [reference=Low]												
High	-0.04 (-0.11, 0.03)	0.25	0.00 (-0.07, 0.06)	0.88	**0.08 (0.06, 0.10)**	**<0.001**	0.03 (-0.01, 0.07)	0.17	0.01 (-0.03, 0.05)	0.70	**0.03 (0.01, 0.04)**	**<0.001**
Tertiary formulary coverage [reference=Low]												
Moderate	-0.05 (-0.13, 0.03)	0.22	**0.08 (0.01, 0.15)**	**0.02**	**0.12 (0.09, 0.14)**	**<0.001**	0.01 (-0.04, 0.06)	0.67	0.04 (0.00, 0.08)	0.08	-0.01 (-0.03, 0.00)	0.05
High	0.00 (-0.08, 0.08)	0.97	-0.02 (-0.09, 0.05)	0.56	**0.04 (0.02, 0.06)**	**<0.001**	0.01 (-0.04, 0.05)	0.83	-0.01 (-0.05, 0.04)	0.82	**0.04 (0.02, 0.06)**	**<0.001**

Abbreviations: CCI, Charlson comorbidity index; coef., coefficient; ED, emergency department; EDSS-DDI, expanded disability status scale disability-derived impairments; IP, inpatient; MA-PD, Medicare Advantage Prescription Drug plan; MS, multiple sclerosis; PA, prior authorization; OP, outpatient; OR, odds ratio; PDP, stand-alone Prescription Drug Plan; SD, standard deviation; ST, step therapy.

Notes: Formulary coverage defined for qualified MS population by quarter and plan type using median 4-quarter moving average proportion of drugs or classes on formulary. Observations for 2020Q2 were dropped from the analysis. Coefficients estimated using multivariable poisson regressions controlling for binary or tertiary formulary coverage, age group, sex, race/ethnicity, region, baseline EDSS-DDI score, baseline CCI score, original reason for Medicare eligibility, proportion of compounds under PA/ST during baseline, and study period in addition to clustering for repeated observations by beneficiary. Race/ethnicity was reported in the Medicare data as Non-Hispanic White, Black (Or African-American), Asian/Pacific Islander, Hispanic, American Indian/Alaska Native, Other, or Unknown. The categories for Asian/Pacific Islander, American Indian/Alaska Native, Other, or Unknown were combined due to small sample size.

© 2025 Blaylock B et al. *JAMA Network Open*.

eTable 15. Regressions of Any MS-Related Utilization (Inpatient, ED, Office Visits) During Follow-Up Quarter (bold indicates statistical significance)

	MA-PD (2019Q1 - 2021Q4)						PDP (2019Q1 - 2022Q4)					
	Any inpatient stays		Any ED visits		Any office visits		Any inpatient stays		Any ED visits		Any office visits	
	aOR (95% CI)	P-value	aOR (95% CI)	P-value	aOR (95% CI)	P-value	aOR (95% CI)	P-value	aOR (95% CI)	P-value	aOR (95% CI)	P-value
Formulary coverage defined by drugs												
Binary formulary coverage [reference=Low]												
High	0.94 (0.88, 1.01)	0.09	**0.93 (0.87, 0.99)**	**0.02**	**1.04 (1.01, 1.07)**	**0.02**	1.01 (0.97, 1.04)	0.69	0.98 (0.95, 1.01)	0.24	**1.06 (1.04, 1.08)**	**<0.001**
Tertiary formulary coverage [reference=Low]												
Moderate	1.02 (0.95, 1.10)	0.57	1.03 (0.96, 1.10)	0.46	**1.29 (1.24, 1.34)**	**<0.001**	1.03 (0.99, 1.08)	0.12	1.03 (0.99, 1.08)	0.12	1.00 (0.98, 1.02)	0.96
High	0.96 (0.89, 1.04)	0.37	0.95 (0.89, 1.02)	0.19	1.02 (0.98, 1.06)	0.31	1.00 (0.96, 1.04)	0.95	**0.96 (0.92, 1.00)**	**0.04**	**1.08 (1.05, 1.11)**	**<0.001**
Formulary coverage defined by drug classes												
Binary formulary coverage [reference=Low]												
High	0.96 (0.89, 1.03)	0.21	0.97 (0.91, 1.03)	0.28	**1.16 (1.12, 1.20)**	**<0.001**	**1.04 (1.00, 1.08)**	**0.04**	1.03 (0.99, 1.06)	0.19	1.02 (1.00, 1.04)	0.10
Tertiary formulary coverage [reference=Low]												
Moderate	0.96 (0.89, 1.04)	0.36	1.05 (0.98, 1.13)	0.15	**1.23 (1.18, 1.27)**	**<0.001**	1.04 (0.99, 1.08)	0.12	1.04 (1.00, 1.09)	0.08	**0.97 (0.95, 0.99)**	**0.01**
High	1.00 (0.93, 1.08)	0.98	0.96 (0.90, 1.03)	0.30	**1.07 (1.03, 1.11)**	**<0.001**	1.02 (0.97, 1.07)	0.47	1.02 (0.98, 1.07)	0.35	**1.03 (1.00, 1.06)**	**0.02**

Abbreviations: aOR, adjusted odds ratio; CCI, Charlson comorbidity index; ED, emergency department; EDSS-DDI, expanded disability status scale disability-derived impairments; IP, inpatient; MA-PD, Medicare Advantage Prescription Drug plan; MS, multiple sclerosis; PA, prior authorization; OP, outpatient; PDP, stand-alone Prescription Drug Plan; SD, standard deviation; ST, step therapy. Notes: Formulary coverage defined for qualified MS population by quarter and plan type using median 4-quarter moving average proportion of drugs or classes on formulary. Observations for 2020Q2 were dropped from the analysis. Adjusted odds ratios estimated using multivariable logistic regressions controlling for binary or tertiary formulary coverage, age group, sex, race/ethnicity, region, baseline EDSS-DDI score, baseline CCI score, original reason for Medicare eligibility, moving average proportion of compounds under PA/ST during baseline, and study period in addition to clustering for repeated observations by beneficiary. Race/ethnicity was reported in the Medicare data as Non-Hispanic White, Black (Or African-American), Asian/Pacific Islander, Hispanic, American Indian/Alaska Native, Other, or Unknown. The categories for Asian/Pacific Islander, American Indian/Alaska Native, Other, or Unknown were combined due to small sample size.

© 2025 Blaylock B et al. *JAMA Network Open*.

eTable 16. Regressions of Count of MS-Related Utilization (Inpatient, ED, Office Visits) During Follow-Up Quarter (bold indicates statistical significance)

	MA-PD (2019Q1 - 2021Q4)						PDP (2019Q1 - 2022Q4)					
	Inpatient stays		ED visits		Office visits		Inpatient stays		ED visits		Office visits	
	coef. (95% CI)	P-value	coef. (95% CI)	P-value	coef. (95% CI)	P-value	coef. (95% CI)	P-value	coef. (95% CI)	P-value	coef. (95% CI)	P-value
Formulary coverage defined by drugs												
Binary formulary coverage [reference=Low]												
High	-0.06 (-0.13, 0.02)	0.14	**-0.09 (-0.16, -0.01)**	**0.02**	**0.04 (0.01, 0.06)**	**0.002**	0.01 (-0.03, 0.05)	0.56	-0.04 (-0.08, 0.00)	0.06	**0.03 (0.02, 0.04)**	**<0.001**
Tertiary formulary coverage [reference=Low]												
Moderate	0.05 (-0.04, 0.13)	0.27	0.01 (-0.08, 0.09)	0.87	**0.21 (0.18, 0.23)**	**<0.001**	0.02 (-0.03, 0.06)	0.44	0.03 (-0.02, 0.08)	0.23	0.00 (-0.02, 0.02)	0.97
High	-0.06 (-0.14, 0.03)	0.19	-0.08 (-0.16, 0.00)	0.06	0.03 (0.00, 0.05)	0.06	0.01 (-0.04, 0.05)	0.77	**-0.06 (-0.11, -0.02)**	**0.009**	**0.04 (0.02, 0.06)**	**<0.001**
Formulary coverage defined by drug classes												
Binary formulary coverage [reference=Low]												
High	-0.04 (-0.11, 0.03)	0.24	-0.06 (-0.13, 0.02)	0.14	**0.12 (0.10, 0.15)**	**<0.001**	0.03 (-0.01, 0.07)	0.16	0.01 (-0.03, 0.06)	0.57	0.01 (-0.01, 0.02)	0.24
Tertiary formulary coverage [reference=Low]												
Moderate	-0.04 (-0.12, 0.05)	0.40	0.05 (-0.04, 0.13)	0.26	**0.16 (0.14, 0.19)**	**<0.001**	0.01 (-0.03, 0.06)	0.61	0.04 (-0.01, 0.09)	0.09	**-0.02 (-0.03, 0.00)**	**0.02**
High	0.00 (-0.08, 0.08)	0.96	-0.06 (-0.13, 0.02)	0.16	**0.07 (0.04, 0.09)**	**<0.001**	0.00 (-0.04, 0.05)	0.84	0.01 (-0.05, 0.06)	0.79	0.01 (0.00, 0.03)	0.15

Abbreviations: CCI, Charlson comorbidity index; coef., coefficient; ED, emergency department; EDSS-DDI, expanded disability status scale disability-derived impairments; IP, inpatient; MA-PD, Medicare Advantage Prescription Drug plan; MS, multiple sclerosis; PA, prior authorization; OP, outpatient; OR, odds ratio; PDP, stand-alone Prescription Drug Plan; SD, standard deviation; ST, step therapy.

Notes: Formulary coverage defined for qualified MS population by quarter and plan type using median 4-quarter moving average proportion of drugs or classes on formulary. Observations for 2020Q2 were dropped from the analysis. Coefficients estimated using multivariable poisson regressions controlling for binary or tertiary formulary coverage, age group, sex, race/ethnicity, region, baseline EDSS-DDI score, baseline CCI score, original reason for Medicare eligibility, proportion of compounds under PA/ST during baseline, and study period in addition to clustering for repeated observations by beneficiary. Race/ethnicity was reported in the Medicare data as Non-Hispanic White, Black (Or African-American), Asian/Pacific Islander, Hispanic, American Indian/Alaska Native, Other, or Unknown. The categories for Asian/Pacific Islander, American Indian/Alaska Native, Other, or Unknown were combined due to small sample size.

© 2025 Blaylock B et al. *JAMA Network Open*.
