# Supplementary material for: Formulary Restrictions and Relapse Episodes in Persons With Relapsing-Remitting Multiple Sclerosis
Source: JAMA Netw Open. 2025 Aug 1;8(8):e2525155. doi: 10.1001/jamanetworkopen.2025.25155 (PMC12317357; doi:10.1001/jamanetworkopen.2025.25155)
Supplement: Supplement 2. — SAS Code for the EDSS-DDI Score [file jamanetwopen-e2525155-s002.pdf]

Supplemental Online Content

Blaylock B, Van Nuys K, Joyce G. Formulary restrictions and relapse episodes in persons with relapsing-remitting multiple sclerosis. *JAMA Netw Open*. 202;8(8):e2525155. doi:10.1001/jamanetworkopen.2025.25155

SAS Code for the EDSS-DDI Score

© 2025 Blaylock B et al. JAMA Network Open.

```
/******************************************************************************
/
/* Program: EDSS_DDI_score.sas
*/
/* Purpose: To generate EDSS-DDI MS severity score 0-10
*/
/* Author: Joanne Wu, University of Southern California
*/
/* Created: 12/06/2023                                   */
/* Updated: August 2024 by Barbara Blaylock, Blaylock Health Economics
LLC                                                          */
/******************************************************************************
/
/* This macro was based on the NCI.comorbidity.macro.sas available from
the US National Cancer Institute (2021).                    */
/* NCI website:
https://healthcaredelivery.cancer.gov/seermedicare/considerations/macro-
2021.html                                        */
/******************************************************************************
/
/* This sas macro scans ICD-10 diagnosis codes from all claims to
generate EDSS-DDI system function score (Truong et al, 2021),    */
/* then create a modified EDSS score ranging 0-10.
*/
/*                                    */
/* The claims dataset to run this macro should be one patient with one
observation with the following variables:       */
/* 1) Patient id                                  */
/* 2) List of ICD 10 codes within a checked period, e.g., 2017
*/
/* 3) MS-related death (death_ms) coded as 1= yes, 0=no
*/
/******************************************************************************
/
/* References                                       */
/* National Cancer Institute. Comorbidity SAS Macro (2021 version).
Available from:              */
/*
https://healthcaredelivery.cancer.gov/seermedicare/considerations/macro-
2021.html. Last accessed April 17, 2025.       */
/* Truong CTL, Le HV, Kamauu AW, et al. Creating a real-world data,
United States healthcare claims-based adaptation of Kurtzke    */
/* Functional Systems Scores for assessing multiple sclerosis severity
and progression. Adv Ther. 2021;38(9):4786-4797.     */
/******************************************************************************
/

%macro mkedss(indata, id, startdate, enddate, claimstartdate, dxvarlist,
outdata);

/* List functional systems (fs) */
%let fs = PF CF BF SF BBF VF MF;
%let fs_items = PF1 PF2 PF3 PF4 PF6 PF8 PF9 PF10 PF12 PF13 PF14 PF15
```

© 2025 Blaylock B et al. JAMA Network Open.

```
PF16
  PF17 PF18 PF20 PF21 PF22 PF24 PF25 PF26 PF28 PF29 PF30 PF31 PF32 PF33
  PF34 CF1 CF2 CF4 CF5 CF6 BF1 BF3 BF4 BF5 BF6 BF9 BF10 BF11
  BF12 BF13 BF14 BF15 BF17 BF18 BF21 BF22 BF25 BF26 BF27 BF28 BF29 BF30
  BF31 BF33 BF34 BF35 BF36 BF39 BF40 BF41 BF42 BF43 BF46 BF47 BF48 BF49
  BF50 BF51 BF52 BF53 SF1 SF2 SF3 SF4 SF6 BBF2 BBF3 BBF4 BBF5 BBF6
  BBF7 BBF9 BBF10 BBF11 BBF12 BBF13 BBF14 BBF15 BBF17 BBF18 BBF19 BBF20
BBF21 BBF22
  BBF23 BBF25 VF1 VF2 VF3 VF4 VF5 VF6 VF7 VF8 VF11 VF12 VF13 VF14
  VF15 VF16 VF20 VF21 VF22 VF23 VF24 VF25 VF26 VF27 VF30 VF31 VF32 VF33
  VF34 VF35 VF36 VF37 VF40 VF41 VF42 VF43 VF44 VF47 VF48 VF49 VF50 VF51
  VF52 VF53 VF54 VF55 MF1 MF2 MF3 MF4 MF7 MF8 MF9 MF10 MF11 MF12
  MF14 MF15 MF17 MF18 MF19 MF20 MF21 MF23 MF24 MF27 MF30 MF31 MF33;

data claims (rename=(&startdate.=Start_date &enddate.=End_date));
set &indata. (keep=&id. &startdate. &enddate. &claimstartdate.
&dxvarlist.);

where &startdate. <= &claimstartdate. <=&enddate.;
inwindow=1;

* initial fs items to 0 *;
length &fs_items. 3.;
array _fsit &fs_items.;
do over _fsit;
 _fsit=0;
end;

* check diagnosis codes and assign fs score *;
array dxcodes(*) $ &dxvarlist.;
do i=1 to dim(dxcodes);
 if not missing(dxcodes(i)) then do;
  dxcode = upcase(dxcodes(i));
  if dxcode in ( "R260", "R261", "R2689", "R269" ) then PF1 = 1 ;
  if dxcode=  "R262" then    PF2 = 1 ;
  if dxcode in ( "G8381", "G8384", "G8389" ) then  PF3 = 1 ;
  if dxcode=  "G839" then    PF4 = 1 ;
  if dxcode=  "R6889" then    PF6 = 1 ;
  if dxcode=  "G8310" then    PF8 = 1 ;
  if dxcode in ( "G8311", "G8312" ) then   PF9 = 1 ;
  if dxcode in ( "G8313", "G8314" ) then   PF10 = 1 ;
  if dxcode=  "G8320" then    PF12 = 1 ;
  if dxcode in ( "G8321", "G8322" ) then   PF13 = 1 ;
  if dxcode in ( "G8323", "G8324" ) then   PF14 = 1 ;
  if dxcode=  "G8330" then    PF15 = 1 ;
  if dxcode=  "Z5189" then    PF16 = 1 ;
  if dxcode=  "Z5189" then    PF17 = 1 ;
  if dxcode=  "G8220" then    PF18 = 1 ;
  if dxcode=  "G8110" then    PF20 = 1 ;
  if dxcode in ( "G8111", "G8112" ) then   PF21 = 1 ;
  if dxcode in ( "G8113", "G8114" ) then   PF22 = 1 ;
  if dxcode=  "G8190" then    PF24 = 1 ;
  if dxcode in ( "G8191", "G8192" ) then   PF25 = 1 ;
  if dxcode in ( "G8193", "G8194" ) then   PF26 = 1 ;
```

© 2025 Blaylock B et al. JAMA Network Open.

```
if dxcode=  "G8190" then    PF28 = 1 ;
if dxcode in ( "G8191", "G8192" ) then   PF29 = 1 ;
if dxcode in ( "G8193", "G8194" ) then   PF30 = 1 ;
if dxcode=  "Z993" then    PF31 = 1 ;
if dxcode=  "G8250" then    PF32 = 1 ;
if dxcode=  "G8250" then    PF33 = 1 ;
if dxcode=  "Z7401" then    PF34 = 1 ;
if dxcode=  "R293" then    CF1 = 1 ;
if dxcode in ( "G250", "G251", "G252" ) then  CF2 = 1 ;
if dxcode=  "G111" then    CF4 = 1 ;
if dxcode=  "G3281" then    CF5 = 1 ;
if dxcode in ( "R270", "R278", "R279" ) then  CF6 = 1 ;
if dxcode=  "H5502" then    BF1 = 1 ;
if dxcode=  "H5509" then    BF3 = 1 ;
if dxcode=  "H5503" then    BF4 = 1 ;
if dxcode=  "H5509" then    BF5 = 1 ;
if dxcode=  "H5504" then    BF6 = 1 ;
if dxcode=  "G521" then    BF9 = 1 ;
if dxcode=  "G521" then    BF10 = 1 ;
if dxcode=  "G522" then    BF11 = 1 ;
if dxcode=  "G528" then    BF12 = 1 ;
if dxcode=  "G523" then    BF13 = 1 ;
if dxcode=  "G527" then    BF14 = 1 ;
if dxcode=  "G529" then    BF15 = 1 ;
if dxcode=  "G508" then    BF17 = 1 ;
if dxcode=  "G509" then    BF18 = 1 ;
if dxcode in ( "G512", "G514", "G518" ) then  BF21 = 1 ;
if dxcode=  "G519" then    BF22 = 1 ;
if dxcode=  "H4900" then    BF25 = 1 ;
if dxcode=  "H4900" then    BF26 = 1 ;
if dxcode=  "H4910" then    BF27 = 1 ;
if dxcode=  "H4920" then    BF28 = 1 ;
if dxcode=  "H4940" then    BF29 = 1 ;
if dxcode=  "H4930" then    BF30 = 1 ;
if dxcode=  "Z5189" then    BF31 = 1 ;
if dxcode=  "R471" then    BF33 = 1 ;
if dxcode=  "R4782" then    BF34 = 1 ;
if dxcode in ( "R4702", "R4781", "R4789" ) then  BF35 = 1 ;
if dxcode=  "I69922" then    BF36 = 1 ;
if dxcode=  "I69921" then    BF39 = 1 ;
if dxcode=  "I69923" then    BF40 = 1 ;
if dxcode=  "I69928" then    BF41 = 1 ;
if dxcode=  "Z5189" then    BF42 = 1 ;
if dxcode=  "I69991" then    BF43 = 1 ;
if dxcode=  "R1311" then    BF46 = 1 ;
if dxcode=  "R1312" then    BF47 = 1 ;
if dxcode=  "R1313" then    BF48 = 1 ;
if dxcode=  "R1314" then    BF49 = 1 ;
if dxcode=  "R1319" then    BF50 = 1 ;
if dxcode=  "R1310" then    BF51 = 1 ;
if dxcode=  "R4701" then    BF52 = 1 ;
if dxcode=  "I69920" then    BF53 = 1 ;
if dxcode=  "G992" then    SF1 = 1 ;
if dxcode=  "G9589" then    SF2 = 1 ;
```

© 2025 Blaylock B et al. JAMA Network Open.

```
if dxcode=  "I69998" then    SF3 = 1 ;
if dxcode=  "R6889" then    SF4 = 1 ;
if dxcode=  "R6889" then    SF6 = 1 ;
if dxcode in ( "R39191", "R39192", "R39198" ) then  BBF2 = 1 ;
if dxcode=  "R6889" then    BBF3 = 1 ;
if dxcode=  "R3915" then    BBF4 = 1 ;
if dxcode=  "R152" then    BBF5 = 1 ;
if dxcode=  "R3911" then    BBF6 = 1 ;
if dxcode=  "R150" then    BBF7 = 1 ;
if dxcode=  "N3941" then    BBF9 = 1 ;
if dxcode=  "N3946" then    BBF10 = 1 ;
if dxcode=  "N3942" then    BBF11 = 1 ;
if dxcode=  "N39490" then    BBF12 = 1 ;
if dxcode in ( "N39491", "N39492", "N39498" ) then BBF13 = 1 ;
if dxcode=  "N3943" then    BBF14 = 1 ;
if dxcode=  "Z466" then    BBF15 = 1 ;
if dxcode=  "N3281" then    BBF17 = 1 ;
if dxcode=  "N318" then    BBF18 = 1 ;
if dxcode=  "N312" then    BBF19 = 1 ;
if dxcode=  "N319" then    BBF20 = 1 ;
if dxcode=  "N3644" then    BBF21 = 1 ;
if dxcode=  "N319" then    BBF22 = 1 ;
if dxcode=  "N3945" then    BBF23 = 1 ;
if dxcode=  "Z435" then    BBF25 = 1 ;
if dxcode=  "H53419" then    VF1 = 1 ;
if dxcode=  "H53429" then    VF2 = 1 ;
if dxcode=  "H53439" then    VF3 = 1 ;
if dxcode=  "H53459" then    VF4 = 1 ;
if dxcode=  "H543" then    VF5 = 1 ;
if dxcode=  "H5460" then    VF6 = 1 ;
if dxcode=  "H547" then    VF7 = 1 ;
if dxcode=  "H542X11" then    VF8 = 1 ;
if dxcode=  "H5450" then    VF11 = 1 ;
if dxcode=  "H5450" then    VF12 = 1 ;
if dxcode in ( "H5450", "H54512A", "H5452A2" ) then  VF13 = 1 ;
if dxcode=  "H5450" then    VF14 = 1 ;
if dxcode=  "H5450" then    VF15 = 1 ;
if dxcode in ( "H5450", "H54511A", "H5452A1" ) then  VF16 = 1 ;
if dxcode=  "H540X55" then    VF20 = 1 ;
if dxcode=  "H5440" then    VF21 = 1 ;
if dxcode in ( "H540X45", "H540X54" ) then   VF22 = 1 ;
if dxcode=  "H540X44" then    VF23 = 1 ;
if dxcode=  "H5440" then    VF24 = 1 ;
if dxcode in ( "H540X35", "H540X53" ) then   VF25 = 1 ;
if dxcode in ( "H540X34", "H540X43" ) then   VF26 = 1 ;
if dxcode=  "H540X33" then    VF27 = 1 ;
if dxcode=  "H5410" then    VF30 = 1 ;
if dxcode in ( "H541152", "H541225" ) then   VF31 = 1 ;
if dxcode in ( "H541142", "H541224" ) then   VF32 = 1 ;
if dxcode in ( "H541132", "H541223" ) then   VF33 = 1 ;
if dxcode=  "H5410" then    VF34 = 1 ;
if dxcode in ( "H541151", "H541215" ) then   VF35 = 1 ;
if dxcode in ( "H541141", "H541214" ) then   VF36 = 1 ;
if dxcode in ( "H541131", "H541213" ) then   VF37 = 1 ;
```

© 2025 Blaylock B et al. JAMA Network Open.

```
  if dxcode=  "H5450" then    VF40 = 1 ;
  if dxcode=  "H542X22" then    VF41 = 1 ;
  if dxcode=  "H5450" then    VF42 = 1 ;
  if dxcode in ( "H542X12" "H542X21" ) then   VF43 = 1 ;
  if dxcode=  "H548" then    VF44 = 1 ;
  if dxcode=  "H5440" then    VF47 = 1 ;
  if dxcode in ( "H54415A", "H5442A5" ) then   VF48 = 1 ;
  if dxcode in ( "H54415A", "H5442A5" ) then   VF49 = 1 ;
  if dxcode=  "H5440" then    VF50 = 1 ;
  if dxcode in ( "H54414A", "H5442A4" ) then   VF51 = 1 ;
  if dxcode in ( "H5440", "H54414A", "H5442A4" ) then  VF52 = 1 ;
  if dxcode=  "H5440" then    VF53 = 1 ;
  if dxcode in ( "H54413A", "H5442A3" ) then   VF54 = 1 ;
  if dxcode in ( "H54413A", "H5442A3" ) then   VF55 = 1 ;
  if dxcode=  "F0630" then    MF1 = 1 ;
  if dxcode in ( "F061", "F53" ) then   MF2 = 1 ;
  if dxcode=  "F068" then    MF3 = 1 ;
  if dxcode=  "G3184" then    MF4 = 1 ;
  if dxcode=  "R41840" then    MF7 = 1 ;
  if dxcode=  "R41841" then    MF8 = 1 ;
  if dxcode=  "R41842" then    MF9 = 1 ;
  if dxcode=  "R41843" then    MF10 = 1 ;
  if dxcode=  "R41844" then    MF11 = 1 ;
  if dxcode=  "R4189" then    MF12 = 1 ;
  if dxcode=  "Z5189" then    MF14 = 1 ;
  if dxcode=  "Z5189" then    MF15 = 1 ;
  if dxcode in ( "F060", "F068" ) then   MF17 = 1 ;
  if dxcode=  "F068" then    MF18 = 1 ;
  if dxcode=  "F0789" then    MF19 = 1 ;
  if dxcode=  "F09" then    MF20 = 1 ;
  if dxcode=  "G94" then    MF21 = 1 ;
  if dxcode=  "G3189" then    MF23 = 1 ;
  if dxcode=  "G319" then    MF24 = 1 ;
  if dxcode=  "F0281" then    MF27 = 1 ;
  if dxcode=  "F0391" then    MF30 = 1 ;
  if dxcode=  "G309" then    MF31 = 1 ;
  if dxcode=  "G3109" then    MF33 = 1 ;
 end;
end;

drop i dxcode &DXVARLIST;
run;

*** Sort claims by ID;
proc sort data=claims; by &ID; run;

*** Find claim date for each functional system;
data systems;
set claims;
by &ID &CLAIMSTARTDATE;

*** identifies the last (or only) ID variable;
%LET IDvar = %SCAN(&ID,-1);
```

© 2025 Blaylock B et al. JAMA Network Open.

```
*** identifies subjects that have ANY claims in the window;
retain anyclaims;
if first.&IDvar then anyclaims = 0;
if inwindow then anyclaims = 1;

%DO i=1 %TO 166;
 %LET syst = %SCAN(&fs_items,&i);

 if first.&IDvar then do; &syst._date=.; end;
 retain &syst._date;

  if &syst. then do;
  if inwindow and &syst._date=. then &syst._date = &CLAIMSTARTDATE;
    format &syst._date mmddyy10.;
 end;

%END;

 if last.&IDvar then output;
 drop &CLAIMSTARTDATE inwindow;
run;

data &OUTDATA;
set systems;

%DO i=1 %TO 166;
%LET syst = %SCAN(&fs_items,&i);
 &syst. = (&syst._date>.);
%END;

* initial fs to 0 *;
length &fs. 3.;
array _fs &fs.;
do over _fs;
 _fs=0;
end;

* count fs grade *;
/*PF*/
array pfg1 (*) PF1; do i=1 to dim(pfg1); if pfg1(i)=1 then PF=1; end;
drop i;
array pfg2 (*) PF2; do i=1 to dim(pfg2); if pfg2(i)=1 then PF=2; end;
drop i;
array pfg3 (*) PF3 PF4 PF6; do i=1 to dim(pfg3); if pfg3(i)=1 then PF=3;
end; drop i;
array pfg4 (*) PF8 PF9 PF10 PF12 PF13 PF14 PF15 PF16 PF17; do i=1 to
dim(pfg4); if pfg4(i)=1 then PF=4; end; drop i;
array pfg5 (*) PF18 PF20 PF21 PF22 PF24 PF25 PF26 PF28 PF29 PF30 PF31;
do i=1 to dim(pfg5); if pfg5(i)=1 then PF=5; end; drop i;
array pfg6 (*) PF32 PF33 PF34; do i=1 to dim(pfg6); if pfg6(i)=1 then
PF=6; end; drop i;

/*CF*/
array cfg1 (*) CF1; do i=1 to dim(cfg1); if cfg1(i)=1 then CF=1; end;
```

© 2025 Blaylock B et al. JAMA Network Open.

```
drop i;
array cfg2 (*) CF2; do i=1 to dim(cfg2); if cfg2(i)=1 then CF=2; end;
drop i;
/*array cfg3 (*) ; do i=1 to dim(cfg3); if cfg3(i)=1 then CF=3; end;
drop i;*/
array cfg4 (*) CF4 CF5; do i=1 to dim(cfg4); if cfg4(i)=1 then CF=4;
end; drop i;
array cfg5 (*) CF6; do i=1 to dim(cfg5); if cfg5(i)=1 then CF=5; end;
drop i;
/*array cfg6 (*) ; do i=1 to dim(cfg6); if cfg6(i)=1 then CF=6; end;
drop i;*/

/*BF*/
array bfg1 (*) BF1; do i=1 to dim(bfg1); if bfg1(i)=1 then BF=1; end;
drop i;
array bfg2 (*) BF3; do i=1 to dim(bfg2); if bfg2(i)=1 then BF=2; end;
drop i;
array bfg3 (*) BF4 BF5 BF6 BF9 BF10 BF11 BF12 BF13 BF14 BF15 BF17 BF18
BF21 BF22 BF25 BF26
   BF27 BF28 BF29 BF30 BF31; do i=1 to dim(bfg3); if bfg3(i)=1 then
BF=3; end; drop i;
array bfg4 (*) BF33 BF34 BF35 BF36 BF39 BF40 BF41 BF42; do i=1 to
dim(bfg4); if bfg4(i)=1 then BF=4; end; drop i;
array bfg5 (*) BF43 BF46 BF47 BF48 BF49 BF50 BF51 BF52 BF53; do i=1 to
dim(bfg5); if bfg5(i)=1 then BF=5; end; drop i;
/*array bfg6 (*) ; do i=1 to dim(bfg6); if bfg6(i)=1 then BF=6; end;
drop i;*/

/*SF*/
array sfg1 (*) SF1; do i=1 to dim(sfg1); if sfg1(i)=1 then SF=1; end;
drop i;
array sfg2 (*) SF2; do i=1 to dim(sfg2); if sfg2(i)=1 then SF=2; end;
drop i;
array sfg3 (*) SF3; do i=1 to dim(sfg3); if sfg3(i)=1 then SF=3; end;
drop i;
array sfg4 (*) SF4; do i=1 to dim(sfg4); if sfg4(i)=1 then SF=4; end;
drop i;
/*array sfg5 (*) ; do i=1 to dim(sfg5); if sfg5(i)=1 then SF=5; end;
drop i;*/
array sfg6 (*) SF6; do i=1 to dim(sfg6); if sfg6(i)=1 then SF=6; end;
drop i;

/*BBF*/
array bbfg1 (*) BBF2 BBF3; do i=1 to dim(bbfg1); if bbfg1(i)=1 then
BBF=1; end; drop i;
array bbfg2 (*) BBF4 BBF5 BBF6 BBF7; do i=1 to dim(bbfg2); if bbfg2(i)=1
then BBF=2; end; drop i;
array bbfg3 (*) BBF9 BBF10 BBF11 BBF12 BBF13 BBF14; do i=1 to
dim(bbfg3); if bbfg3(i)=1 then BBF=3; end; drop i;
array bbfg4 (*) BBF15; do i=1 to dim(bbfg4); if bbfg4(i)=1 then BBF=4;
end; drop i;
array bbfg5 (*) BBF17 BBF18 BBF19 BBF20 BBF21 BBF22 BBF23; do i=1 to
dim(bbfg5); if bbfg5(i)=1 then BBF=5; end; drop i;
array bbfg6 (*) BBF25; do i=1 to dim(bbfg6); if bbfg6(i)=1 then BBF=6;
```

© 2025 Blaylock B et al. JAMA Network Open.

```
end; drop i;

/*VF*/
array vfg1 (*) VF1 VF2; do i=1 to dim(vfg1); if vfg1(i)=1 then VF=1;
end; drop i;
array vfg2 (*) VF3 VF4; do i=1 to dim(vfg2); if vfg2(i)=1 then VF=2;
end; drop i;
array vfg3 (*) VF5 VF6 VF7; do i=1 to dim(vfg3); if vfg3(i)=1 then VF=3;
end; drop i;
array vfg4 (*) VF8; do i=1 to dim(vfg4); if vfg4(i)=1 then VF=4; end;
drop i;
array vfg5 (*) VF11 VF12 VF13 VF14 VF15 VF16; do i=1 to dim(vfg5); if
vfg5(i)=1 then VF=5; end; drop i;
array vfg6 (*) VF20 VF21 VF22 VF23 VF24 VF25 VF26 VF27 VF30 VF31 VF32
VF33 VF34 VF35
   VF36 VF37 VF40 VF41 VF42 VF43 VF44 VF47 VF48
   VF49 VF50 VF51 VF52 VF53 VF54 VF55; do i=1 to dim(vfg6); if vfg6(i)=1
then VF=6; end; drop i;

/*MF*/
array mfg1 (*) MF1; do i=1 to dim(mfg1); if mfg1(i)=1 then MF=1; end;
drop i;
array mfg2 (*) MF2 MF3 MF4 MF7 MF8 MF9 MF10 MF11 MF12; do i=1 to
dim(mfg2); if mfg2(i)=1 then MF=2; end; drop i;
array mfg3 (*) MF14 MF15; do i=1 to dim(mfg3); if mfg3(i)=1 then MF=3;
end; drop i;
array mfg4 (*) MF17 MF18 MF19 MF20 MF21 MF23 MF24; do i=1 to dim(mfg4);
if mfg4(i)=1 then MF=4; end; drop i;
array mfg5 (*) MF27 MF30 MF31 MF33; do i=1 to dim(mfg5); if mfg5(i)=1
then MF=5; end; drop i;
/*array mfg6 (*) ; do i=1 to dim(mfg6); if mfg6(i)=1 then MF=6; end;
drop i;*/


array fs (*) PF CF BF SF BBF VF MF ;

fsg1=0; do i=1 to dim(fs); if fs(i)=1 then fsg1=fsg1+1; end; drop i;
fsg2=0; do i=1 to dim(fs); if fs(i)=2 then fsg2=fsg2+1; end; drop i;
fsg3=0; do i=1 to dim(fs); if fs(i)=3 then fsg3=fsg3+1; end; drop i;
fsg4=0; do i=1 to dim(fs); if fs(i)=4 then fsg4=fsg4+1; end; drop i;
fsg5=0; do i=1 to dim(fs); if fs(i)=5 then fsg5=fsg5+1; end; drop i;
fsg6=0; do i=1 to dim(fs); if fs(i)=6 then fsg6=fsg6+1; end; drop i;


* according to Truong (2021) Table 1 to assign EDSS score *;
/*All grade 0 in all functional system (FS) scores*/
/*Excludes cerebral function grade 1 */
if (fsg1=0 and fsg2=0 and fsg3=0 and fsg4=0 and fsg5=0 and fsg6=0) OR
 (fsg1=1 and cf1=1 and fsg2=0 and fsg3=0 and fsg4=0 and fsg5=0 and
fsg6=0) then edss=0 ;

/*One FS grade 1*/
/*Excludes cerebral function grade 1 */
if (fsg1=1 and cf1=0 and fsg2=0 and fsg3=0 and fsg4=0 and fsg5=0 and
```

© 2025 Blaylock B et al. JAMA Network Open.

```
fsg6=0) OR
 (fsg1=2 and cf1=1 and fsg2=0 and fsg3=0 and fsg4=0 and fsg5=0 and
fsg6=0) then edss=1.0 ;

/*More than one FS grade 1*/
/*Excludes cerebral function grade 1 */
if (fsg1>1 and cf1=0 and fsg2=0 and fsg3=0 and fsg4=0 and fsg5=0 and
fsg6=0) OR
 (fsg1>2 and cf1=1 and fsg2=0 and fsg3=0 and fsg4=0 and fsg5=0 and
fsg6=0) then edss=1.5 ;

/*One FS grade 2, others 0 or 1*/
if (fsg2>=1 and fsg3=0 and fsg4=0 and fsg5=0 and fsg6=0) then edss=2;

/*Two FS grade 2, others 0 or 1*/
if (fsg2>=2 and fsg3=0 and fsg4=0 and fsg5=0 and fsg6=0) then edss=2.5;

/*(One FS grade 3, others 0 or 1) or (three or four FS grade 2, others 0
or 1)*/
if (fsg3>=1 and fsg2=0 and fsg4=0 and fsg5=0 and fsg6=0) OR
 (fsg2>=3 and fsg2=0 and fsg4=0 and fsg5=0 and fsg6=0) then edss=3.0;

/*(One FS grade 3 and one or two FS grade 2) or (two FS grade 3 and
others 0 or 1) or (five grade 2 and others 0 or 1)*/
if (fsg3>=1 and fsg2>=1 and fsg4=0 and fsg5=0 and fsg6=0) OR
 (fsg3>=2 and fsg2=0 and fsg4=0 and fsg5=0 and fsg6=0) OR
 (fsg2>=5 and fsg3=0 and fsg4=0 and fsg5=0 and fsg6=0) then edss=3.5;

/*One FS grade 4 and others 0 or 1*/
if (fsg4>=1 and fsg2=0 and fsg3=0 and fsg5=0 and fsg6=0) then edss=4.0;

/*One FS grade 4 (others <= 3)*/
if (fsg4>=1 and fsg5=0 and fsg6=0) then edss=4.5;

/*(One grade 5 alone, others 0 or 1); or (two grade 4 and others < 3)*/
if (fsg5>=1 and fsg2=0 and fsg3=0 and fsg4=0 and fsg6=0) OR
 (fsg4>=2 and fsg3=0 and fsg5=0 and fsg6=0) then edss=5.0;

/*One FS grade 5+; others FS grades <= 3*/
if (fsg5+fsg6>=1 and fsg4=0) then edss=5.5;

/*(Three FS grade 3+; others FS grades <= 3) or (two FS grade 4+; others
FS grades <= 3)*/
if (fsg3+fsg4+fsg5+fsg6>=3) OR (fsg4+fsg5+fsg6>=2) then edss=6.0;

/*Three FS grade 3+ with pyramidal function grade 4; others FS grades <
3*/
if (fsg3+fsg4+fsg5+fsg6>=3 and PF>=4) then edss=6.5;

/*Two FS grade 4+ with pyramidal function grade 4+; others FS grades <
4*/
if (fsg4+fsg5+fsg6>=2 and PF>=4) then edss=7.0;

/*Three FS grade 4+ with pyramidal function grade 4+; others FS grades <
```

© 2025 Blaylock B et al. JAMA Network Open.

```
4*/
if (fsg4+fsg5+fsg6>=3 and PF>=4) then edss=7.5;

/*Four FS grade 4+ with pyramidal function grade 6; others FS grades <
4*/
if (fsg4+fsg5+fsg6>=4 and PF>=6) then edss=8.0;

/*Five FS grade 4+ with pyramidal function grade 6; others FS grades <
4*/
if (fsg4+fsg5+fsg6>=5 and PF>=6) then edss=8.5;

/*Six FS grade 4+ with pyramidal function grade 6; others FS grades <
4*/
if (fsg4+fsg5+fsg6>=6 and PF>=6) then edss=9.0;

/*All FS grade 4+ with pyramidal function grade 6*/
if (fsg4+fsg5+fsg6>=7 and PF>=6) then edss=9.5;

/*if death_ms=1 then edss=10.0; */

label
edss="EDSS score"
fsg1="Functional system grade 1"
fsg2="Functional system grade 2"
fsg3="Functional system grade 3"
fsg4="Functional system grade 4"
fsg5="Functional system grade 5"
fsg6="Functional system grade 6"
;

run;
%mend;
```

© 2025 Blaylock B et al. JAMA Network Open.
