# Supplementary material for: Formulary Restrictions and Relapse Episodes in Persons With Relapsing-Remitting Multiple Sclerosis
Source: JAMA Netw Open. 2025 Aug 1;8(8):e2525155. doi: 10.1001/jamanetworkopen.2025.25155 (PMC12317357; doi:10.1001/jamanetworkopen.2025.25155)
Supplement: Supplement 3. — Data Sharing Statement [file jamanetwopen-e2525155-s003.pdf]

Data Sharing Statement

Blaylock. Formulary Restrictions and Relapse Episodes in Persons With Relapsing-Remitting Multiple Sclerosis. *JAMA Netw Open*. Published August 01, 2025.
doi:10.1001/jamanetworkopen.2025.25155

Data
Data available: No

Additional Information
Explanation for why data not available: The Center for Medicare and Medicaid Services (CMS) public use file (PUF) Part D formularies 2019-2024 are publicly available and can be accessed via CMS (https://data.cms.gov/). Drug information by National Drug Code (NDC) can be accessed via the Food and Drug Administration (FDA) (https://dps.fda.gov/ndc). The 100% Medicare sample was accessed via the CMS virtual research data center (VRDC) and is not publicly available.
